# KDM5B promotes SMAD4 loss-driven drug resistance through activating DLG1/YAP to induce lipid accumulation in pancreatic ductal adenocarcinoma

**DOI:** 10.1038/s41420-024-02020-4

**Published:** 2024-05-24

**Authors:** Yumin Wang, Shiqian Liu, Yan Wang, Baibei Li, Jiaming Liang, Yu Chen, Bo Tang, Shuiping Yu, Hongquan Wang

**Affiliations:** 1 Department of Hepatobiliary Surgery, The First Affiliated Hospital of Guangxi Medical University, Nanning, 530021 Guangxi P. R. China; 2 Pharmaceutical College Guangxi Medical University, Nanning, 530021 Guangxi P. R. China; 3 Hunan Provincial Key Laboratory of Hepatobiliary Disease Research & Division of Hepato-Biliary-Pancreatic Surgery, Department of Surgery, The Second Xiangya Hospital of Central South University, Changsha, 410011 P. R. China

**Keywords:** Pancreatic cancer, Pancreatic cancer

## Abstract

Inactivated suppressor of mothers against decapentaplegic homolog (SMAD) 4 significantly affects cancer development in pancreatic ductal adenocarcinoma (PDAC). However, the contribution of smad4 loss to drug resistance in PDAC is largely undetermined. In the present study, we reported that the loss of SMAD4 endows PDAC cells the ability to drug resistance through upregulating histone lysine demethylase, Lysine-Specific Demethylase 5B (KDM5B, also known as JARID1B or PLU1). Upregulated KDM5B was found in PDAC, associated with poor prognosis and recurrence of PDAC patients. Upregulated KDM5B promotes PDAC tumor malignancy, i.e. cancer cells stemness and drug resistance in vitro and in vivo, while KDM5B knockout exerts opposite effects. Mechanistically, loss of Smad4-mediated upregulation of KDM5B promotes drug resistance through inhibiting the discs-large homolog 1 (DLG1), thereby facilitating nuclear translocation of YAP to induce de novo lipogenesis. Moreover, m^6^A demethylase FTO is involved in the upregulation of KDM5B by maintaining KDM5B mRNA stability. Collectively, the present study suggested FTO-mediated KDM5B stabilization in the context of loss of Smad4 activate DLG1/YAP1 pathway to promote tumorigenesis by reprogramming lipid accumulation in PDAC. Our study confirmed that the KDM5B-DLG1-YAP1 pathway axis plays a crucial role in the genesis and progression of PDAC, and KDM5B was expected to become a target for the treatment of PDAC.

**Figure:**
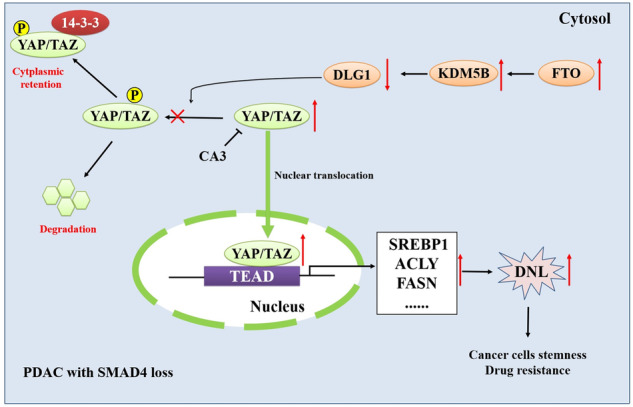
The schematic diagram of KDM5B-DLG1-YAP pathway axis in regulating drug resistance of PDAC to gemcitabine (GEM). In the context of SMAD4 loss PDAC cells, FTO-mediated stabilization and upregulation of KDM5B promotes drug resistance through directly targeting DLG1 to promote YAP1 translocation to nucleus to induce de novo lipogenesis (DNL).

The schematic diagram of KDM5B-DLG1-YAP pathway axis in regulating drug resistance of PDAC to gemcitabine (GEM). In the context of SMAD4 loss PDAC cells, FTO-mediated stabilization and upregulation of KDM5B promotes drug resistance through directly targeting DLG1 to promote YAP1 translocation to nucleus to induce de novo lipogenesis (DNL).

## Introduction

Pancreatic ductal adenocarcinoma (PDAC) representing the most common histological subtype of pancreatic cancer, is one of the most lethal human cancers and is projected to become the second deadliest cancer by 2030, and currently the overall 5-year survival rate is less than 7% [1, 2]. PDAC easily evades current treatment strategies for rapid progression once fully developed, and this aggressiveness result in a disastrous outcome for patients. Therapeutic interventions are largely ineffective. To date, surgery remains the only curative option for pancreatic cancer, only 20% of patients with PDAC present with localized tumors that are resected through surgery. PDAC has its aggressive nature, late diagnosis, and lack of effective therapeutic options for advanced disease, leading to a poor prognosis. These issues highlight the urgent need to further better understand the pathophysiology of PDAC in order to uncover novel vulnerabilities of the cancer cells [3].

The vast majority of PDAC harbors oncogenic mutations in specific genes, suggesting the pivotal roles of these mutations in the tumorigenesis of PDAC [4–6]. Whole-genome sequencing analysis uncovered that SMAD4 were among the most common mutated genes important for PDAC progression [7]. Notably, SMAD4 inactivation occur in around 50%–60% of patients with PDAC, indicating that SMAD4 functions as a specific tumour suppressor in PDAC [8]. It is well established that Smad4 play a role in activating transforming growth factor-β (TGF-β) signalling pathway and translocating to the nucleus as a transcriptional cofactor to facilitate the transcription of TGF-β-responsive genes in cancer cells [9], thereby regulating a variety of critical processes, including tumor development [10] and immunotherapy [11]. Recent studies have revealed important roles for smad4 deficiency as a tumour suppressor in the genesis of colon cancer [12–14], lung cancer [15], and breast cancer [16]. Although emerging evidence has revealed a key role for smad4 deficiency in driving PDAC [17–20], the molecular mechanisms underlying the contribution of smad4 loss to tumor malignancy in PDAC is less well defined.

Epigenetic mechanisms including histone modifications that affect gene transcription, are pivotal to the initiation and progression of cancers [21–23]. Epigenetic factors are also involved in the progression of pancreatic cancer [24]. Therefore, it is of fundamental importance to screen other factors, including epigenetic marks that could influence pancreatic carcinogenesis so as to identify novel therapeutic targets or biomarkers [25]. Mounting evidences suggests the role for epigenetic dysregulation in the initiation and progression of PDAC [26]. Histone lysine methylation, an important epigenetic mechanism associated gene expression profiles, is a reversible process tightly controlled by histone methyltransferases and demethylases to regulate biological functions in cells, have been demonstrated to be critical in numerous cancer types [6, 25, 27–30]. However, the contribution of other Histone methylation mediators to the progression of PDAC remains to be fully elucidated.

The histone lysine demethylase, lysine-specific demethylase 5B that catalyzes the demethylation of histone 3 lysine 4 (H3K4), is a critical regulator of the H3K4-methylome during early mouse embryonic pre-implantation stage development [31]. KDM5B is a jmjc domain-containing histone demethylase that belongs to KDM5 family. KDM5B represses the transcriptional function of genes through erasing the methyl group from H3K4me2/3, which performs wide regulatory effects on chromatin structure. Recent evidence indicates that KDM5B is frequently found upregulated in various human cancers functioning as an oncogene and associates with human cancers closely [32–34]. However, the functional and prognostic roles of KDM5B in the context of loss of Smad4 in PDAC have not been well clarified so far. The contribution of KDM5B to the tumorigenesis of PDAC remains to be fully elucidated. Furthermore, it is still largely unknown how mechanistically KDM5B contributes to PDAC progression. Thus, there is an immediate need to identify new targets for the treatment of PDAC.

Here, we demonstrate that KDM5B promotes PDAC cancer cells stemness and drug resistance. Loss of SMAD4-mediated upregulation of KDM5B promotes drug resistance to gemcitabine (GEM) through inhibiting DLG1, thereby facilitating nuclear translocation of YAP to induce de novo lipogenesis. Moreover, m^6^A demethylase FTO participated in the upregulation of KDM5B by maintaining KDM5B mRNA stability. Taken together, the present study suggested FTO-mediated KDM5B stabilization in the context of loss of SMAD4 activate DLG1/YAP1 pathway to promote tumorigenesis by reprogramming lipid accumulation in PDAC, highlighting a therapeutic strategy for SMAD4-negative PDAC through targeting KDM5B.

## Results

### KDM5B is upregulated in gemcitabine-treated PDX pancreatic cancer with SMAD4 loss and correlates with poor prognosis

**Fig. 1:**
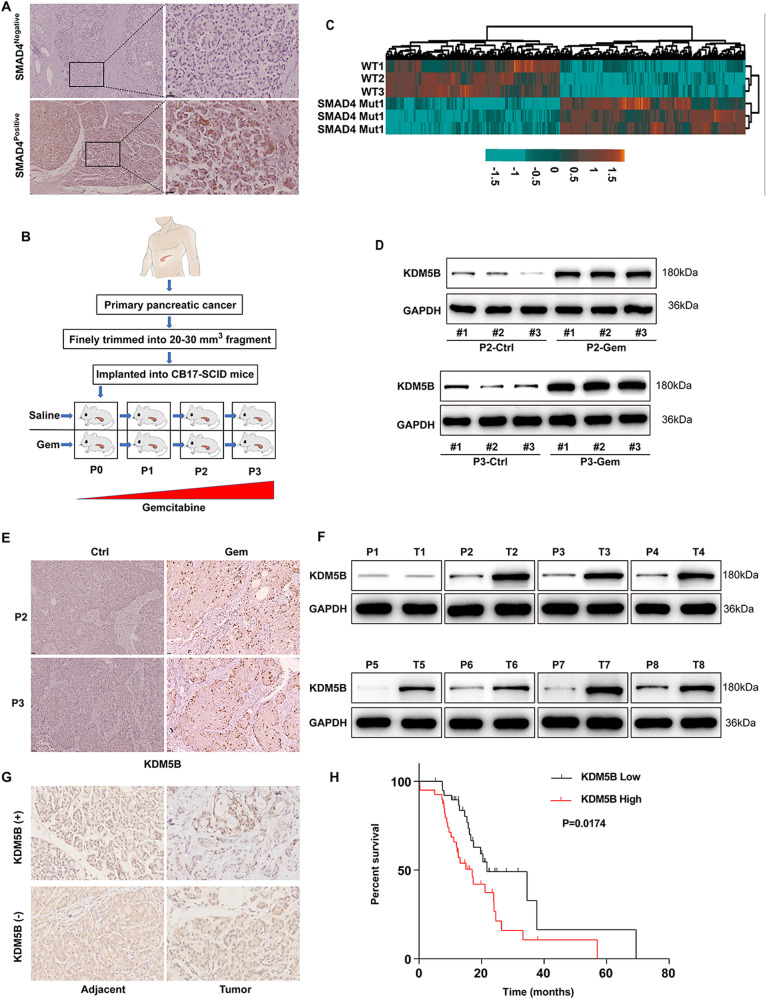
KDM5B is overexpressed in PDAC with SMAD4 loss. **A** Immunohistochemistry (IHC) staining for SMAD4 in surgically resected primary pancreatic cancer tissue. **B** Schematic protocol of patient-derived xenograft (PDX) approach challenged by gemcitabine (GEM). **C** The heat map shows the differential expression of RNAs in GEM-treated and control P3-PDX mice. **D** Immunoblotting of KDM5B in cells isolated from PDX mice of 2 or 3 passages with or without GEM treatment. GAPDH was included as a loading control. **E** Representative images of IHC staining for KDM5B in tumor tissue from PDX mice of 2 passages with or without GEM treatment. Immunoblotting (**F**) and IHC staining (**G**) for KDM5B in sections from human PDAC or paracarcinoma tissue. GAPDH was included as a loading control. **H** Kaplan-Meier analysis of survival time based on the KDM5B expression in 70 PDAC patients.

Previous studies have shown that SMAD4 loss correlates with clinical outcome and resistance to chemotherapy. To figure out the underling mechanism, we established a gemcitabine (GEM)-treated PDX SMAD4 mutations pancreatic cancer mouse model. We first surgically resected primary pancreatic cancer tissue, IHC assay was used to show the SMAD4 mutations in tumor tissues (Fig. 1A). The pancreatic cancer tissue was finely trimmed and directly transplanted into CB17-SCID mice, which were randomized and treated with either saline (vehicle) or GEM every generation (Fig. 1B). In order to characterize the global changes in SMAD4-dependent transcriptome, we carried out a genome-wide RNA sequencing (RNA-seq). RNA-seq results revealed 801 upregulated genes and 732 downregulated genes (Log |FC|≥1, *P* < 0.05) were identified in P3-PDX treated with control or gemcitabine (Fig. 1C). KDM5B stood out among the most significantly differentially expressed genes (Fig. S1A). KDM5B protein levels are consistently found to be significantly higher in GEM-treated PDXs, as measured by immunoblotting (Fig. 1D), and quantifying immunostaining (Fig. 1E).

Through the Cancer Genome Atlas (TCGA) database, we found that KDM5B was upregulated in PDACs screened in the PDAC tissues (179 cases) and normal tissues (171 cases) (Fig. S1B). To determine whether KDM5B expression is correlated with the development and progression of PDAC, 91 pairs of tumors and non-cancerous pancreatic tissues were subjected for detection of KDM5B mRNA by RT-PCR (Fig. S1C) and protein by western blot (Fig. 1F and Fig. S1D), KDM5B was elevated in PDACs. This observation was corroborated by the IHC results, showing an obviously upregulated expression of KDM5B protein (Fig. 1G). Together, these data suggest that KDM5B is overexpressed in GEM-treated PDX pancreatic cancer with SMAD4 loss.

We then investigated the prognostic value of KDM5B. Clinicopathologic variables were evaluated for association with expression of KDM5B (Table 1), the expression levels of KDM5B were robustly correlated with tumor size (*P* = 0.018), TNM staging (*P* = 0.023), HbA1C (*P* = 0.043), and TG (*P* = 0.035), TC (*P* = 0.028). Importantly, the Kaplan-Meier survival analysis showed that PDAC patients with high KDM5B expression had shorter overall survival (*P* = 0.0174) (*n* = 65 Fig. 1H). The results were consistent with results from, Kaplan-meier Plotter and TCGA database (Fig. S1F, G). These results indicated that KDM5B is highly expressed in PDAC and correlated with a worse prognosis.

**Table 1:** Relationship between KDM5B and clinicopathological parameters in 65 PDAC patients.

Variables	All cases	KDM5B expression		*P*
		Low (*n* = 32)	High (*n* = 33)	
Age (years)				
<50	35	15	20	0.267
≥50	30	17	13	
Gender				
Male		20	23	0.540
Female		12	10	
Alcohol history				
No		25	27	0.710
Yes		7	6	
Smoking				
No		14	9	0.165
Yes		18	24	
Tumor size (cm)				
<4		20	12	0.049
≥4		13	21	
TNM stage				
I–II		19	10	0.026
III–IV		14	23	
Lymph node invasion				
Absent		26	15	0.005
Present		7	18	
Metastasis				
No		28	16	0.002
Yes		5	17	

### KDM5B promotes PDAC cells stemness and proliferation

To gain an insight into the function of KDM5B in PDAC with SMAD4 loss, we examined the effect of KDM5B on PDAC in vitro and in vivo. Firstly, we analyzed the endogenous protein (Supporting Fig. S2A) levels of KDM5B in PDAC cell lines. PDAC cell lines with SMAD4 loss was BxPC-3, CFPAC-1, Capan-1 [17]. PDAC cell lines BxPC-3 and CFPAC-1 were transfected with lentiviral vectors encoding human shRNAs KDM5B inserts, Capan-1 with KDM5B inserts. Representative WB and RT-PCR showing the transfection efficiency (Supporting Fig. S2B–E).

**Fig. 2:**
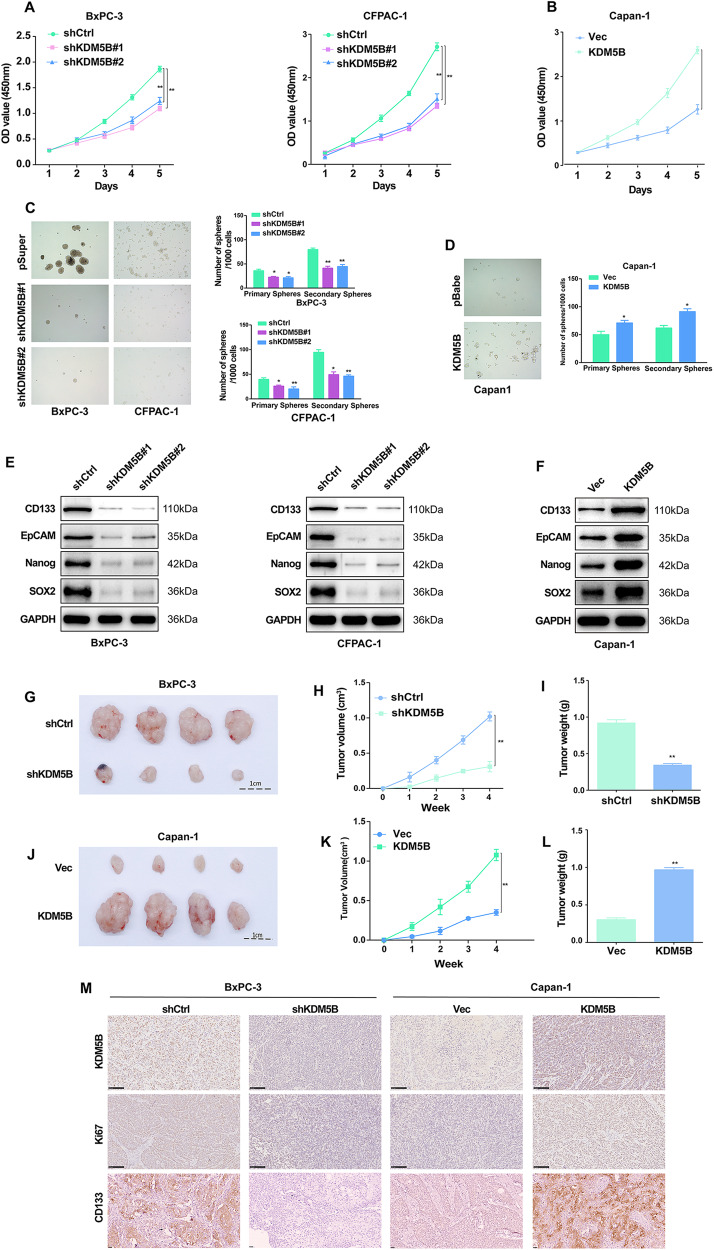
KDM5B Promotes PDAC Cells Stemness and proliferation. Cell proliferation analysis using CCK8 in KDM5B silencing BxPC-3 and CFPAC-1 cell (**A**) or Capan-1 cells with KDM5B overexpression (**B**). **C** Representative phase contrast images of tumorspheres formed by BxPC-3 and CFPAC-1 cells transduced with KDM5B shRNA constructs or a nontarget shRNA. **D** Representative phase contrast images of tumorspheres formed by Capan-1 cells transduced with KDM5B constructs or Control. The expression levels of CSC markers (CD133, EpCAM, NANOG and SOX2) were examined in shKDM5B-transfected BxPC-3 and CFPAC-1 cells (**E**) and KDM5B overexpression plasmid-transfected Capan-1 cells (**F**) by immunoblotting. **G**–**I** Tumor formation in nude mice injected with shKDM5B-transfected BxPC-3 cells (5 × 10^4^ cells/mouse). The xenograft tumor volume and weight was monitored for 4 weeks. **J**–**L** Tumor formation in nude mice injected with KDM5B overexpression Capan-1 cells (5 × 10^4^ cells per mouse). The xenograft tumor volume and weight was monitored for 40 days. **M** The expression level of KDM5B, Ki67, and CD133 were tested by IHC in different groups.

Silencing KDM5B dramatically suppressed proliferation of PDAC cells (Fig. 2A), whereas overexpression of KDM5B promoted cell proliferation (Fig. 2B). In PDAC cells, silencing KDM5B reduced spheroid formation ability compared with control (Fig. 2C). Conversely, KDM5B overexpression promotes the spheroid formation ability in Capan-1 cells (Fig. 2D). We also examined the effect of KDM5B on the expression of stemness markers. KDM5B silencing markedly reduced the protein expression of representative stemness-associated genes, including CD133, EpCAM, NANOG and SOX2 in PDAC cells (Fig. 2E and Fig. S2G). While, KDM5B overexpression significantly upregulated the expression of these stemness markers (Fig. 2F and Fig. S2G).

Consistent with the above results, KDM5B knockdown reduced tumorigenesis in vivo, showing that BXPC-3 cells with KDM5B knockdown displayed reduced tumor growth rate, size and Ki67, CD133 positive cells, compared to the control group, respectively (Fig. 2G–I, M). Overexpression of KDM5B accelerated the tumor growth of increased tumor growth and increased the number of Ki67, CD133 positive cells (Fig. 2J–M). These findings indicated that KDM5B promotes PDAC cells stemness and proliferation.

### KDM5B suppresses the chemosensitivity of PDAC cells to GEM

**Fig. 3:**
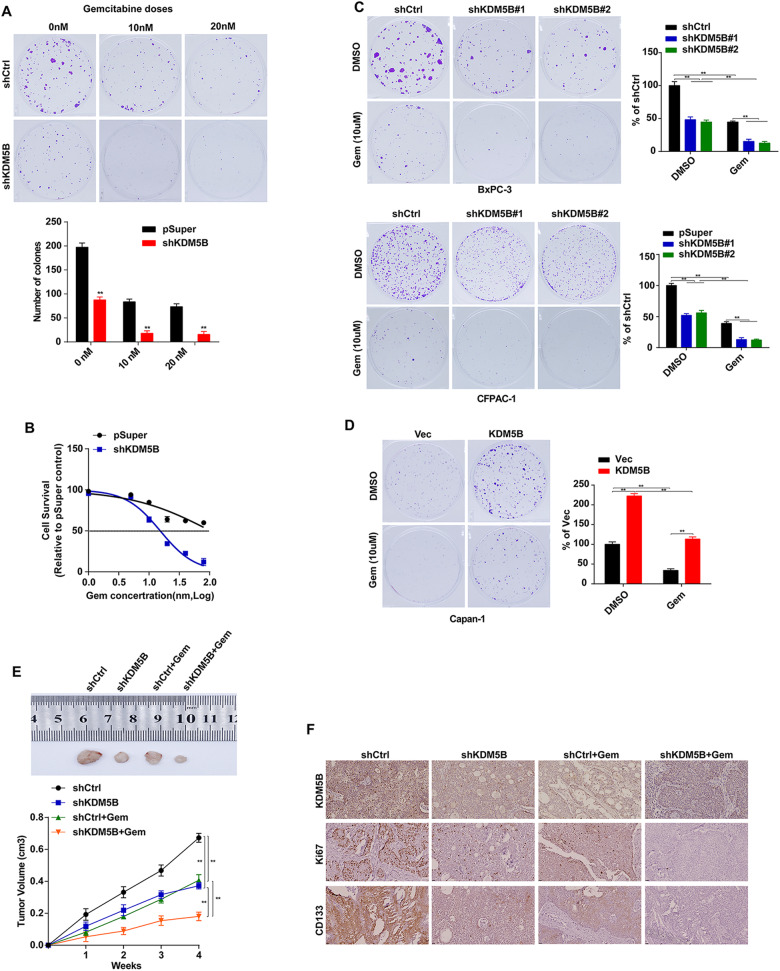
KDM5B suppresses the chemosensitivity of PDAC cells. **A** Colony formation of BxPC-3 cells transfected with control (upper panel) or shKDM5B (bottom panel) in the present of indicated doses of GEM. **B** Dose response curves of GEM in KDM5B silencing BxPC-3 cells. **C** Colony formation of KDM5B silencing CFPAC-1 and BxPC-3 cells after GEM treatment. **D** Colony formation of KDM5B overexpression Capan-1 cells after GEM treatment. **E** Representative images of xenograft tumors formed by in KDM5B silencing BxPC-3 cells in GEM-treated nude mice. Tumor growth curves of BxPC-3 cells transfected with shCtrl or shKDM5B in GEM-treated nude mice. **F** IHC was used to assess the levels of KDM5B, Ki67, and CD133 in different groups as described in **E**.

Cancer stemness are an important cause of tumor recurrence and drug resistance. Then, we further assess the effect of KDM5B on sensitivity of PDAC to GEM. PDAC cells were exposed to a series of concentrations of GEM, colony-formation was performed. Knockdown of KDM5B sensitized BXPC-3 cells to GEM, while KDM5B overexpression prompted resistance in Capan-1 cells (Fig. 3A–D). To further investigate whether the expression of KDM5B affects chemosensitivity in vivo, KDM5B deficiency evidently decreased tumor growth rate in GEM treated nude mice xenografts, meanwhile, the combination of shKDM5B with GEM treatment had stronger inhibitory effect on tumor growth comparing with any individual treatment (Fig. 3E). In addition, consistent with the tumor growth rate, the combination of shKDM5B with GEM led to less CD133-positive cell number and ki67-positive cell number than other group (Fig. 3F). Together, these data suggest that KDM5B depletion sensitizes the cancer cells to chemotherapeutic agents and decreases drug resistance.

### KDM5B promotes de novo lipogenesis in PDAC cells

**Fig. 4:**
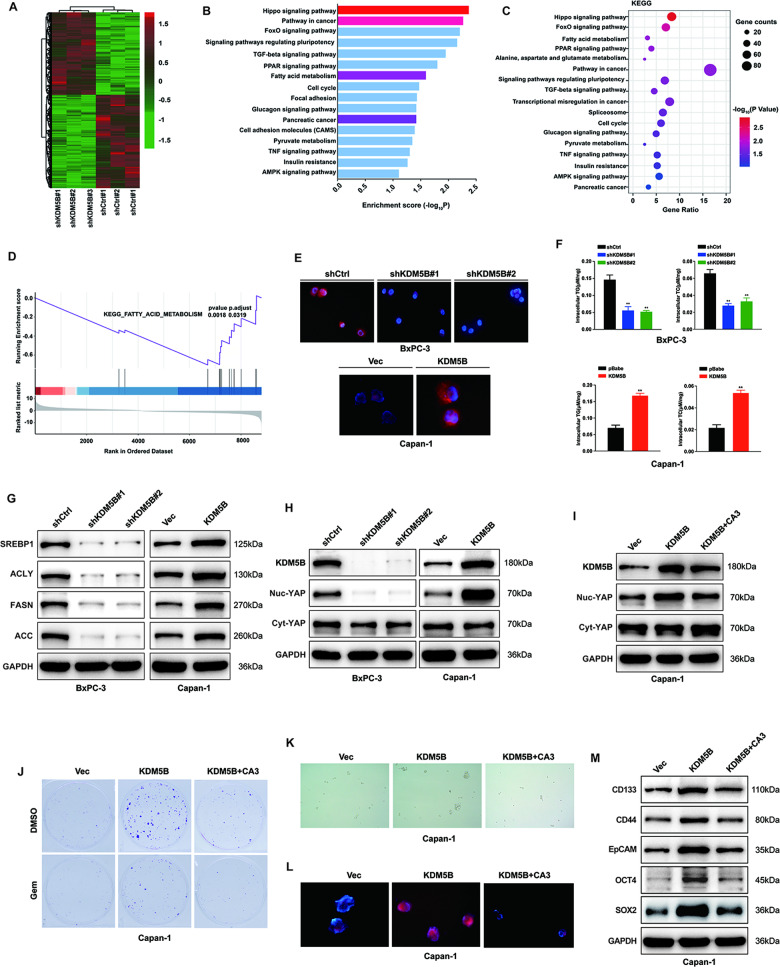
KDM5B enhance de novo lipogenesis in PDAC. **A** The heatmap summarizes the differentially expressed genes in KDM5B WT and KDM5B silencing BxPC-3 cells. **B** KEGG enrichment analysis of KDM5B-regulated genes. **C** KEGG pathway enrichment analysis of target genes by a bubble chart. **D** Enrichment of an fatty acid metabolism gene expression signature in GSEA analysis of genes altered as described as described in **A**–**C**. **E** Nile red staining assay for levels of neutral lipids in the indicated cells. DAPI (blue) was used to stain the nucleus. **F** The quantitative detection of triglyceride and cholesterol in KDM5B silencing BxPC-3 cells and KDM5B overexpressed Capan-1 cells. **G** Western blot analyses of SREBP1, ACLY, FASN and ACC1 in KDM5B silencing BxPC-3 cells and KDM5B overexpressed Capan-1 cells. **H** The cell lysates from BxPC-3 cells transfected with shKDM5B or Capan-1 cells transfected with KDM5B overexpression plasmid were fractionated into Cytosol/Nucleus fractions for immunoblotting. **I** Western blot to determine the YAP protein levels in Cytosol/Nucleus fractions in Capan-1 cells transfected with KDM5B overexpressed Capan-1 cells in the presence or absence of CA3 (an inhibitor of YAP) challenge. **J** Colony formation of KDM5B overexpressed Capan-1 cells after GEM treatment in the presence or absence of CA3 challenge. **K** Representative phase contrast images of tumorspheres formed in the indicated cells as described in **I**. **L** Nile red staining assay for levels of neutral lipids in the indicated cells as described in **I**. DAPI (blue) was used to stain the nucleus. **M** Immunoblotting to detect stemness marker expression in the indicated cells as described in **I**.

To explore the potential mechanisms of KDM5B regulating malignant behavior of PDAC, we performed an RNA-seq in BXPC-3 cells transfected with either shKDM5B or a negative control shRNA (shCtrl). The KEGG pathway enrichment analysis revealed that the differentially expressed gene (|LogFC|≥1, P < 0.05, Fig. 4A) sets were significantly related to fatty acid synthesis signaling pathway and Hippo signaling pathway (Fig. 4B, C). GSEA analysis indicated the differentially expressed gene sets also is related to lipid metabolism (Fig. 4D). Next we evaluate whether KDM5B regulates de novo lipogenesis in PDAC cells, the content of lipids was measured in KDM5B expression manipulated PDAC cells. The results showed a significant decrease in lipid accumulation in BxPC-3 cells transfected with shKDM5B by Nile red staining (Fig. 4E). Accordinigly, KDM5B overexpression in Capan-1 cells significantly increased the intensity of neutral lipid stained by Nile red staining (Fig. 4E). Consistently, KDM5B overexpression or knockdown led to increased or decreased elevated cellular triglyceride (TG) and TC levels (Fig. 4F). Consistent with decreased lipid accumulation, knockdown of KDM5B decreased the expression of sterol regulatory element binding protein 1 (SREBP1), ATP citrate lyase (ACLY), fatty acid synthase (FASN) and Acetyl-CoA Carboxylase 1 (ACC1) in KDM5B silencing BxPC-3 cells (Fig. 4G). In accordance with increased lipid accumulation, KDM5B overexpression upregulates the expression of SREBP1, ACLY, FASN and ACC1 in KDM5B overexpressed Capan-1 cells (Fig. 4G). These data suggest that KDM5B promote de novo lipogenesis in PDAC cells.

Accumulating studies reported that Hippo signaling pathway plays important roles in the cancer stemness maintainance, chemoresistance, lipid metabolism and tumor development [35]. We found that silencing KDM5B inhibits the Hippo signaling pathway in PDAC cells, evidenced by YAP nuclear localization were decreased after KDM5B knockdown BxPC-3 cells, consistent with canonical Hippo signaling (Fig. 4H). Overexpression of KDM5B in Capan-1 cells led to increased YAP nuclear localization (Fig. 4H). To confirm KDM5B-mediated metabolic regulation of stemness and chemosensitivity is channeled through Hippo-YAP pathway, we used CA3 (an inhibitor of YAP) in the KDM5B-overexpressed PDAC cells. YAP1 signal transduction inhibitor CA3 reversed the KDM5B overexpression-mediated increased YAP translocation to nucleus (Fig. 4I). CA3 reversed KDM5B overexpression-mediated increased chemosensitivity to GEM (Fig. 4J), increased spheroid formation ability (Fig. 4K), elevated cellular triglyceride (TG) and TC levels (Fig. 4L and Fig. S3B, C), and upregulated stemness marker expression (Fig. 4M). Taken together, these data suggest that KDM5B regulates stemness-related chemosensitivity and lipid metabolism by activating Hippo-YAP pathway.

### DLG1 is a mediator for KDM5B-induced malignant phenotype in PDAC cells via Hippo/YAP pathway

**Fig. 5:**
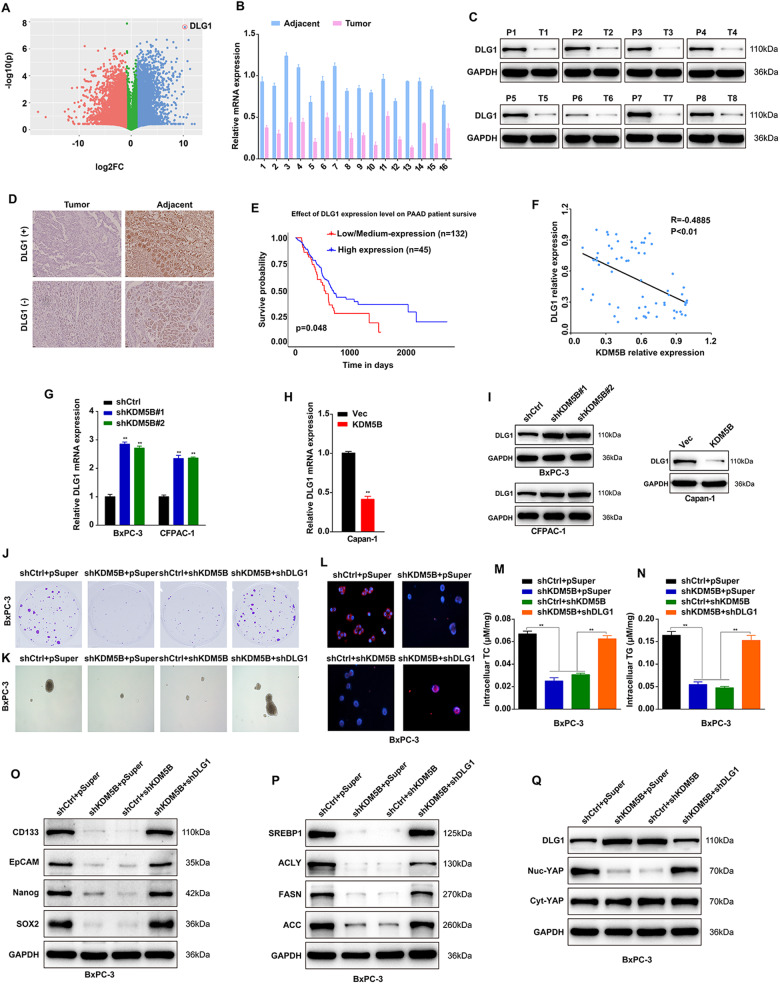
DLG1 is a mediator for KDM5B-induced malignant phenotype in PDAC cells via Hippo pathway. **A** Volcano plot of differentially expressed genes between BxPC-3 cells shNC and shKDM5B cells as determined by RNA-seq. **B** Comparison of DLG1 expression in cancer tissues and paired pericarcinomatous normal tissues of 16 patients with PDAC. **C** Immunoblotting for DLG1 in sections from human PDAC or paracarcinoma tissue. GAPDH was included as a loading control. **D** IHC staining for DLG1 in sections from human PDAC or paracarcinoma tissue. **E** Kaplan-Meier analysis of survival time in PDAC patients based on the high (Blue; *n* = 45) or low (Red; *n* = 132) DLG1 expression. **F** Correlation between mRNA levels of DLG1 and KDM5B in 65 PDAC tissues were analysed by Scatter plot. **G**–**I** RT-qPCR analysis an western blot detects the expression of DLG1 in KDM5B silencing BxPC-3 and CFPAC-1 cell or Capan-1 cells with KDM5B overexpression. BxPC-3 cells transfected with indicated construct and then analyze clone formation ability (**J**), cell sphere number (**K**), neutral lipids by Nile red staining (**L**), and contents of cholesterol (**M**) and triglyceride (**N**). Immunoblotting of lysates from BxPC-3 cells as described in **J**. Expression of stemness marker (**O**), de novo lipogenesis (**P**), and YAP protein levels in Cytosol/Nucleus fractions (**Q**) were analyized.

To uncover the mechanism underlying KDM5B regulates Hippo-YAP pathway, we analyze the RNA-seq data with KDM5B depletion. A total of 1547 genes were upregulated whereas 1806 genes were downregulated, with DLG1 was the most significantly one (Fig. 5A). DLG1 is a key member of the membrane-associated guanylate kinase (MAGUKs) family containing specific protein recognition domains including Src homology 3 (SH3), PDZ and homologous guanylate kinase (GuK) regions [36]. DLG1 plays a significant role in cell adhesion and tight junction through acting as key scaffolds at cell membranes for protein complexes [36, 37]. We investigated the genome-wide distribution of KDM5B binding sites in PDAC cells using ChIP-Seq. Then, we surveyed the overlapped gene sets between the RNA-seq data and the ChIP-seq data and found that 160 upregulated and 249 downregulated genes were included in the set of KDM5B target gene. Interestingly, DLG1 was found in the set of 83 upregulated target genes. Emerging studies have revealed that the DLG is involved in Hippo-YAP pathway in cancers. Therefore, we selected DLG1 for subsequent analysis. To exam the levels of expression of DLG1 in 72 pairs of tumors and non-cancerous pancreatic tissues, we determined DLG1 expression level with RT-qPCR assay, western blot assay and IHC assay. The DLG1 expression level was less abundant in pancreatic cancer tissues compared with para-cancerous tissues (Fig. 5B–D). The Kaplan-Meier survival analysis in TCGA showed that PDAC patients with less DLG1 expression had shorter overall survival (*P* = 0.048) (Fig. 5E). Additionally, a strong negative correlation between DLG1 mRNA levels and the expression of KDM5B was observedin PDAC tumor tissue (Fig. 5F). We then assess whether KDM5B regulate DLG1 by western blot and RT-PCR, and showing that silencing KDM5B significantly upregulates, whereas overexpression of KDM5B downregulates the expression of DLG1 level (Fig. 5G–I), implying that KDM5B may regulate DLG1 expression at the transcriptional level.

To ascertain whether DLG1 is a major contributor to the function of KDM5B in PDAC tumorigenesis stemness, chemosensitivity and lipid metabolism, we transfected KDM5B-silenced BxPC-3 cells and KDM5B-overexpressed Capan-1 cells with DLG1 shRNA plasmid or DLG1 overexpression plasmid. DLG1 knockdown restored the KDM5B depletion induced changes in proliferation (Fig. S4A), colony formation (Fig. 5J and Fig. S4B), spheroid formation ability (Fig. 5K), lipid content (Fig. 5L), levels of intracellular TG and TC (Fig. 5M, N), stemness marker expression (Fig. 5O), lipid metabolism marker expression (Fig. 5P and Fig. S4C) in BxPC-3 cells. As expected, DLG1 knockdown restored the KDM5B depletion-mediated decreased YAP1 translocation to nucleus (Fig. 5Q). Consistent with our aforementioned experiments, we identified overexpression of KDM5B effectively suppressed the Hippo-YAP pathway. By contrast, DLG1 overexpression rescued the KDM5B overexpression-induced changes in Capan-1 cells (Fig. S4B–K). Conversely, we examined the expression of nucleus YAP (Nuc-YAP), and cytosol YAP (Cyc-YAP), overexpression of DLG1 abolished the KDM5B-induced YAP1 translocation to nucleus (Fig. S4L). Together, these data suggest that KDM5B promotes stemness, chemosensitivity and lipid metabolism in PDAC cells through downregulating DLG1, thereby facilitating YAP translocation to nucleus.

### KDM5B as a potential therapeutic target for PDAC

**Fig. 6:**
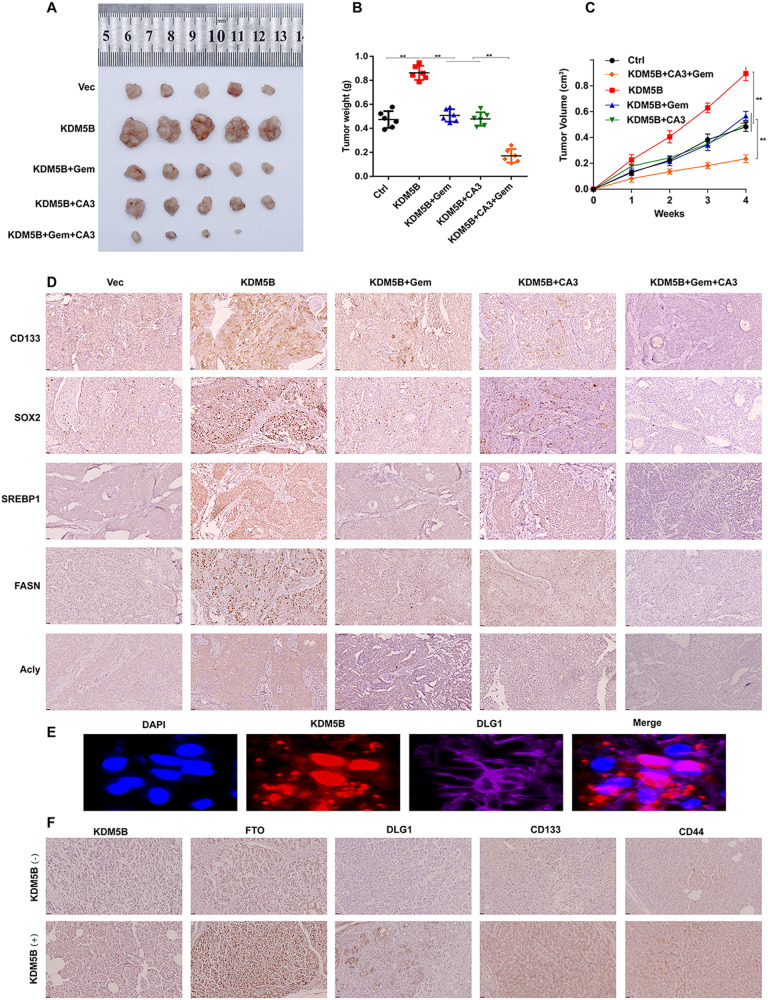
KDM5B as a potential therapeutic target for PDAC progression and resistance. **A** Representative images of xenograft tumors formed by Capan-1 cells transfected with or without KDM5B in GEM treated nude mice in the presence or absence of CA3 challenge. Tumor weights (**B**) and volume (**C**) of mouse xenografts as described in **A**. **D** Typical images of IHC staining for CD133, SOX2, SREBP1, FASN and ACLY in tumor sections from PDAC tissues as described in **A**. **E** A immunofluorescence assay was performed to detect the localization of KDM5B and DLG1 in BxPC-3 cells. **F** Typical images of IHC staining for indicated proteins in xenografts tumor with or without KDM5B knockdown.

To investigate the role of KDM5B in the growth of PDAC in vivo, we performed cell-based xenograft studies. Capan-1 cells with stable overexpression of KDM5B (KDM5B) or empty vector (Vec) were subcutaneously inoculated into the dorsal flanks of nude mice (n = 5 per group). Overexpression of KDM5B remarkably accelerated the tumor growth of Capan-1 cells and became resistant to gemcitabine (Fig. 6A–C). In contrast, the combination of GEM with CA3 reduced tumor growth and restored sensitivity to GEM therapy in KDM5B-overexpression tumors (Fig. 6A–C). Consistent with these changes, IF and IHC confirmed the expression of KDM5B, and markers of stemness, lipid metabolism and Hippo pathway in xenografts(Fig. 6D–F). Collectively, these data suggest that KDM5B is a potential target for the treatment of PDAC and drug resistance.

### m^6^A modification mediated by FTO upregulates KDM5B

**Fig. 7:**
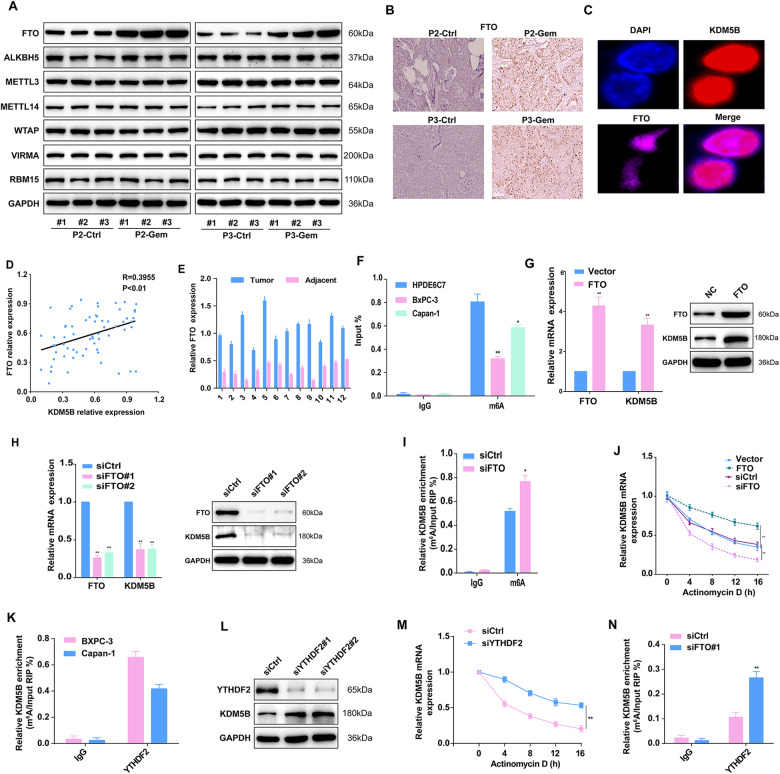
m^6^A modification induced by FTO upregulates KDM5B. **A** Immunoblotting was used to detect the expression of indicated m^6^A demethylases and methylases in cells isolated from PDX mice of 2 and 3 passages treated with or without GEM. **B** Representative IHC staining images for FTO in tumor tissue from PDX treated with or without GEM. **C** A immunofluorescence assay was performed to detect the localization of FTO and KDM5B in BxPC-3 cells. **D** Scatter plot analysis of correlation between mRNA levels of FTO and KDM5B in 65 PDAC tissues. **E** Comparison of FTO expression in PDAC tissues of 12 patients with paired pericarcinomatous normal tissues. **F** MeRIP-qPCR results showed that the m^6^A enrichment of KDM5B was higher in BxPC-3 and Capan-1 cells than in HPDE6C7 cells. Effect of FTO overexpression in Capan-1 cells (**G**) and knockdown in BxPC-3 cells (**H**) on the expression of KDM5B. **I** Effect of FTO silencing the degree of m^6^A enrichment of KDM5B. **J** Effect of FTO silencing and overexpression on KDM5B stability in the presence of actinomycin. **K** RIP assay confirms the binding between YTHDF2 and KDM5B using anti-YTHDF2 antibodies in BxPC-3 and Capan-1 cells. **L** Protein level of KDM5B in PDAC cells was affected by the m^6^A binding proteins YTHDF2. **M** Effect of YTHDF2 silencing and overexpression on KDM5B stability in the presence of actinomycin. **N** Knockdown of FTO increased the m^6^A methylation in KDM5B mRNA by the YTHDF2-RIP analysis.

Subsequently, we explored the mechanism underlying the upregulation of KDM5B. The dysregulation of m^6^A has been linked to a number of diseases including multiple cancers. Given the distribution of m^6^A peaks along KDM5B in between P3-PDX treated with control or GEM, we hypothesized that the m^6^A mechanism might also contribute to the oncogenic effect of KDM5B in PDAC. To test whether KDM5B is modified with m^6^A, we performed an RNA-seq assay of P3-PDX treated with control or gemcitabine, showing that FTO was one of the top upregulated genes (Fig. S5A). Consistent with this, our western blot analysis suggests that FTO protein levels were upregulated in GEM-treated PDXs (Fig. 7A and Fig. S5B, C), which was corroborated by IHC assay (Fig. 7B). The Co-immunofluorescence results confirmed the colocalization of KDM5B with FTO in PDAC (Fig. 7C). Interestingly, expression of FTO is positively correlated with the level of KDM5B in PDAC (Fig. 7D). We found that FTO levels were significantly upregulated in PDAC tissues than in corresponding para-cancerous tissues (Fig. 7E). Publicly available TCGA and GEPIA database further confirmed these data (Fig. S5D, E).

To assess whether KDM5B was regulated by FTO-mediated m^6^A modification, we detected the KDM5B and its m^6^A level in clinical PDAC samples. As expected, the level of KDM5B were higher in PDAC cancer tissues than in normal adjacent tissues, in contrast, the m^6^A level of KDM5B were lower. m^6^A RNA IP (RIP) combined with qRT-PCR revealed that m^6^A was significantly less enriched in BXPC-3 and Capan-1 cells than in normal HPDE6C7 cells (Fig. 7F). Furthermore, FTO overexpression increases the mRNA and protein levels of KDM5B (Fig. 7G), while silencing FTO decreases the mRNA and protein levels of KDM5B (Fig. 7H). The MeRIP-qPCR results showed that silencing of FTO increased the m^6^A modification of KDM5B in PDAC cells (Fig. 7I). FTO depletion decreased the stability of KDM5B, whereas FTO overexpression produced the opposite result in the present of actinomycin D (an agent that blocks the de novo synthesis of RNA) (Fig. 7J). YTHDF2 is implicated in mRNA degradation and has been found to prevent m^6^A modification of FTO protein through binding to the m^6^A site in the nucleus. A direct interaction between YTHDF2 and KDM5B mRNA was confirmed by RIP assay in PDAC cells (Fig. 7K). YTHDF2 silencing in PDAC cells significantly increased the KDM5B level (Fig. 7L). YTHDF2 silencing promotes KDM5B stability After in the presence of actinomycin D (Fig. 7M). Furthermore, the interaction between YTHDF2 and KDM5B was remarkably inhibited after FTO depletion (Fig. 7N). These data suggested that the m^6^A site was essential for FTO-mediated KDM5B expression.

## Discussion

Pancreatic cancer is one of the most malignant tumors, with extremely poor prognosis. With the development of clinical diagnosing technology and increasing therapeutic efficacy, there has been a decrease in morbidity and mortality, yet a large proportion of patients are found to be in advanced stages, losing the opportunity for radical surgery, especially in the rapid progression. SMAD4 inactivation occur in around 50%–60% of patients with PDAC, indicating that SMAD4 functions as a specific tumour suppressor in PDAC [8]. Although emerging evidence has revealed a key role for SMAD4 deficiency in driving PDAC [17–20], the molecular mechanisms underlying the contribution of SMAD4 loss to tumor malignancy in PDAC is less well defined. Here, we revealed a novel role of KDM5B drive drug resistance to GEM via activating DLG1/hippo-YAP to induce lipid accumulation to mediate malignancy in PDAC in the context of SMAD4 loss.

KDM5B is a demethylase that has been shown to play a carcinogenic role in a variety of tumors, such as glioma, liver cancer, gastric cancer, colon cancer and laryngeal cancer [34, 38–42]. However, few studies have been conducted in PDAC, and the mechanism involved in the development of pancreatic cancer remains to be further elucidated. We revealed that upregulated KDM5B was observed in patients with PDAC bearing SMAD4 loss, and correlates with poor prognosis. We found that KDM5B enhances cancer cell proliferation and cancer cells stemness. These findings substantiate previous indications that KDM5B stimulates cancer cells stemness [43–45].

Cancer cells stemness contributes to drug resistance in cancer. Our present data suggest that KDM5B promotes drug resistance of PDAC cell to GEM. These findings substantiate previous studies that indicates that KDM5B enhances cancer drug resistance in neuroblastoma Cells [44, 46], glioblastoma [47], hepatocellular carcinoma [45], melanoma [48], and gastric cancer [49]. However, the machinery underlying the KDM5B enhances cancer drug resistance is obscure and are still being elucidated. We further study have revealed that KDM5B promote de novo lipogenesis in PDAC cells. Study found that HIPPO-YAP pathway play role in the genesis of cancers [50] and mediate lipogenesis reprogramming promotes hepatocellular carcinoma progression [51]. And we for the first time have shown that Hippo-YAP pathway accounts for KDM5B -mediated promotion of de novo lipogenesis in PDAC. KDM5B promotes Hippo-YAP translocation to nuclear to upregulates enzymes for de novo lipogenesis.

The discs-large homolog 1(DLG) belongs to the modular proteins Membrane Associated Guanylate Kinases (MAGUKs) and plays a crucial role in the formation of cell-cell junctions, cell polarity and tissue morphogenesis [52].DLG1 is a scaffolding protein that play crucial roles in the control of important cellular processes, like the control of cell polarity, tissue growth, differentiation, cell migration [53, 54]. In particular, the loss of cell polarity has been considered as a basic step in tumorigenesis. DLG1 has historically been characterized as a tumor suppressor. DLG1 has historically been characterized as a tumor suppressor [55, 56]. However, recently years, DLG1 has been found to be expressed differently among malignant tumors and to play a different role in the progression of tumors and sometimes even opposite role [57–59]. High expression of DLG1 was found to be associated with tumor invasiveness in colon cancer [36], while some scholars have found that DLG1 played an inhibitory role in liver cancer and inhibited its metastasis [60].So far, the role of DLG1 in pancreatic cancer and its related mechanism have not been reported. In this study, we found that DLG1 was significantly downregulated in PDAC cancer tissues with SMAD4 loss. We for the first time confirmed that KDM5B negatively regulates the expression DLG1. DLG1 function as a mediator for KDM5B-induced malignant phenotype in PDAC with SMAD4 loss via Hippo/YAP pathway. And KDM5B promotes stemness, chemosensitivity and lipid metabolism in PDAC cells through downregulating DLG1 to facilitate YAP1 translocation to nucleus.

N6-methyladenosine (m^6^A) modifications are dynamic and reversible posttranscriptional RNA modifications that are mediated by m^6^A regulators, i.e., methyltransferases (“writers”), demethylases (“erasers”), and m^6^A-binding proteins (“readers”) [61]. m^6^A modifications play a role in tumorigenesis and progression of multiple human malignancies including cancer drug resistance [62]. Mounting studies have shown that m^6^A methylation modulates oncogene or tumor suppressor expression levels in cancers [63, 64]. So we wonder whether KDM5B is regulated by m^6^A in PDAC. We found that FTO protein levels were upregulated in PDAC. The expression of FTO is positively correlated with the level of KDM5B in PDAC. Increased FTO promotes demethylation of KDM5B in PDAC cells. Silencing FTO decreases the levels of KDM5B and increase its m^6^A modification. These studies indicates that FTO acts as an m^6^A modulator and plays a critical role in maintaining the high level of KDM5B in PDAC. YTHDF2 is implicated in mRNA degradation and has been found to prevent m^6^A modification of FTO protein through binding to the m^6^A site in the nucleus. We also found that the m^6^A of KDM5B can be read by YTHDF2, which negatively regulates KDM5B. These data suggested that the m^6^A site was essential for FTO-mediated KDM5B expression under the help of YTHDF2.

In conclusion, we first identified a novel KDM5B-DLG1-YAP pathway axis in regulating drug resistance of PDAC to gemcitabine (GEM) in the context of SMAD4 loss PDAC cells. In the context of SMAD4 loss PDAC cells, FTO-mediated stabilization and upregulation of KDM5B promotes drug resistance through directly inhibiting DLG1, thereby promoting YAP1 translocation to nucleus to induce de novo lipogenesis (DNL).

## Materials and methods

### Human tissue specimens and cell lines

A total of 106 paired, paraffin-embedded primary specimens diagnosed with PC according to their clinical pathological characteristics were included in the study. The patients were operated at the first affiliated hospital of Guangxi Medical University (GXMU) between 2009 and 2018. 91 pairs of fresh PC tissues were obtained from the first affiliated hospital of Guangxi Medical University (GXMU) were stored at −80 °C immediately after surgery. The study was approved by the Ethics Committee of the Provincial Clinical College of GXMU, Guangxi, China. We obtained informed consent from all patients.

Human pancreatic cell (HPDE6C7) and various human pancreatic cancer cell lines (AsPC-1, PANC-1, SW1990, BxPC-3, MIA PaCa-2, CFPAC-1, Capan-1) were purchased from the American Type Culture Collection (ATCC; Manassas, VA, USA). Once cell lines were recovered, we cultured all cells mentioned above in Dulbecco’s Modified Eagle’s Medium (DMEM) containing 10% fetal bovine serum (FBS) and 1% antibiotic/antifungal solution (Biowest, Nuaillé, France).

### Immunohistochemistry

91 paired, paraffin-embedded primary specimens were stained for KDM5B by immunohistochemistry (IHC), as described previously [65]. The sections were incubated with anti-KDM5B (dilution ratio, 1:1000; No. ab181089 Abcam, Cambridge, UK) at 4°C overnight followed by secondary anti-rabbit HRP-conjugated antibody. The expression changes of KDM5B were evaluated blindly by two professional pathologists. Cancer cells were counted under the microscope as previously described [65].

### Quantitative reverse-transcriptase polymerase chain reaction (qRT-PCR)

Fast Start Universal SYBR Green Master Mix (Roche Diagnostics GmbH, Mannheim, Germany) was used for qRT-PCR. Total RNA was extracted using Trizol reagent (Invitrogen), and complementary DNA (cDNA) was synthesized using SuperScript II Reverse Transcriptase (Invitrogen). Quantitative reverse transcription-PCR (qRT-PCR) and data collection were performed with an ABI PRISM 7900HT sequence detection system.

### Western blotting

Homogenized tissues or cells were lysed in ice-cold radioimmunoprecipitation assay buffer containing protease inhibitors. The bicinchoninic acid (BCA) method was used for determination. After separation on the sodium dodecyl sulfate-polyacrylamide gel electrophoresis (SDS-PAGE) gels, we transferred the proteins to a polyvinylidene fluoride (PVDF) membrane (Bio-Rad Laboratories, Hercules, CA, USA) followed by the membrane was incubated with primary antibody at 4°C overnight. After incubating the membrane for 2 h in secondary antibody, we photographed it and visualized reaction bands using ECL detection reagents.

### Cell migration and invasion assay

Transwell chambers (pore size, 8 μm; BD Biosciences, Franklin Lakes, NJ, USA) were used to detect cell migration and cell invasion. For both types of assays, cells (2 × 10^5^) in 200 μl of serum-free medium were plated into the upper chambers and 500 μl medium containing serum was added to the bottom chambers. After 24 h incubation, cells that had migrated to the lower surface of the membrane were fixed with paraformaldehyde and then stained with crystal violet to allow visualization. The difference with the matrigel invasion assays, the trans well insert was not coated with Matrigel in Motility assays.

### Cell proliferation assays

Cells were seeded into 96-well plates (1 × 10^3^ cells/well) 24 h after transfection. Plates were incubated in a humid incubator at 37 °C for 1, 2, 3, 4, 5, and 6 days. Then 20 μL MTT at a concentration of 5 mg/ml in phosphate-buffered saline (PBS) was added into each well. The plates were incubated at 37 °C for a further 4 h, after which we removed the medium and added 150 μL dimethyl sulfoxide (DMSO) to each well. The absorbance was read at 490 nm by the Synergy HIM microplate reader (BioTek Instruments, Inc., Winooski, VT, USA).

### Plate colony-forming and holoclonal, and sphere-forming assays

Clonal, clonogenic, and sphere formation assays were performed as described previously [66].

### Animal experimentation

BABL/c nude mice (average age, 6 weeks old) were acquired from Shanghai Laboratory Animal Center (SLAC) Co., Ltd., Shanghai, China. All animal protocols were performed in accordance with National Institute of Health Guide for the Care and Use of Laboratory Animals with the approval of the GXMU Ethics Review Committee. To assess in vivo tumor growth, 2 × 10^6^ cells were injected subcutaneously into each mouse and tumors were measured weekly for 6 weeks, after which tumors were resected for weighed and volume.

### Chromatin immunoprecipitation-seq and ChIP-PCR

Chromatin immunoprecipitation (ChIP) kits were purchased from Millipore, and ChIP experiments were performed as previous described [65]. Immunoprecipitated DNA was analyzed on an ABI PRISM 7900HT sequence detection system.

### Microarray analysis

Total RNAs were extracted from the KDM5B-knockdown and control cells using an RNeasy Mini Kit (QIAGEN, Hilden, Germany) per the manufacturer’s protocol. The data were initially normalized by robust multiarray average (RMA) normalization algorithms in expression console software (Affymetrix). Raw reads were treated with a custom Perl script to remove adapters and reads for quality control. Significant differential expression of genes was selected with a P-value cutoff of 0.05 and the genes up- and downregulated≥4-fold. Gene Ontology (GO; http://www.geneontology.org) and Kyoto Encyclopedia of Genes and Genomes (KEGG; http://www.genome.jp/kegg) analyses were performed for differentially expressed genes.

### Statistical analysis

Statistical analysis was carried out using the SPSS 22.0 software. Comparisons between different groups were calculated by Student t-test or one-way ANOVA, Mann–Whitney *U* test. The Kaplan-Meier was used to analysis of clinical prognosis was used Kaplan-Meier. The correlation analysis was used the Pearson’s correlation. Statistical significance was set as p < 0.05. Data were recorded as means ± SD.

### Supplementary information

Sup Fig and legends

Unprocessed WB images.pdf

## Data Availability

All data generated or analyzed during this study are included in this published article.
