# Supplementary material for: KDM5B promotes SMAD4 loss-driven drug resistance through activating DLG1/YAP to induce lipid accumulation in pancreatic ductal adenocarcinoma
Source: Cell Death Discov. 2024 May 24;10:252. doi: 10.1038/s41420-024-02020-4 (PMC11126577; doi:10.1038/s41420-024-02020-4)
Supplement: Supplementary file 1 — Sup Fig and legends [file 41420_2024_2020_MOESM1_ESM.doc]

**
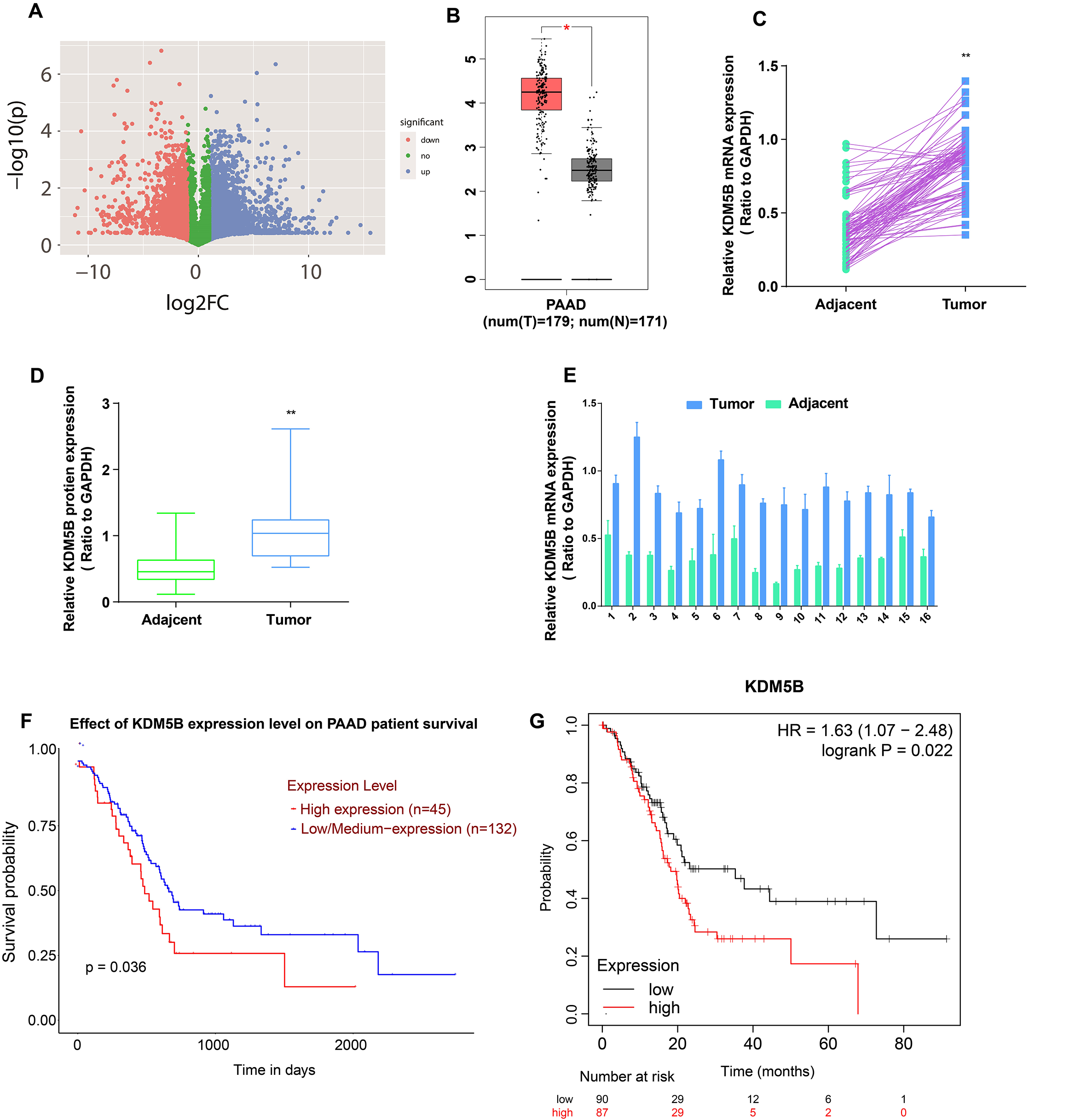
Fig 1S related to Fig 1. (**A)RNA-seq results revealed genes were identified in P3-PDX treated with control or gemcitabine. **(**B) Comparison of KDM5B expression between 179 PDAC tissues and 171 adjacent normal pancreatic tissues based on TCGA dataset. **(**C) RT-qPCR analysis of KDM5B expression in 72 pairs of PDAC tissues and adjacent normal tissues. **(**C) Immunoblotting for KDM5B in sections from human PDAC or paracarcinoma tissue. Comparison of KDM5B expression in cancer tissues of 16 HCC PDAC patients with paired pericarcinomatous normal tissues. (F) Kaplan-Meier analysis of survival time in PDAC patients based on the high (red; n=45) or low (black; n=132) KDM5B expression. (G) Kaplan-Meier analysis indicating recurrence probability of PDAC patients with high (red) or low (black) KDM5B expression.

**
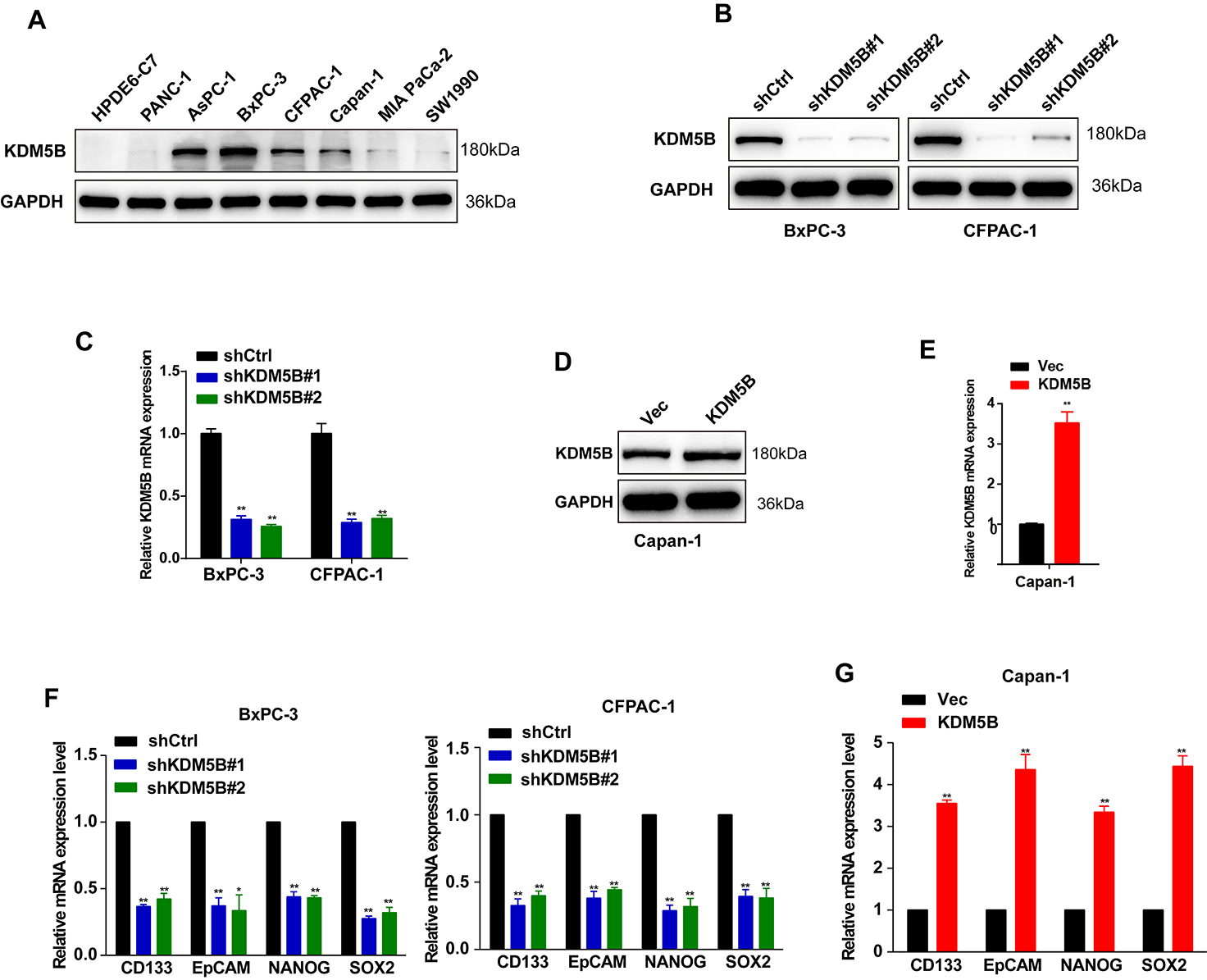
**

**Fig 2S related to Fig 2.** **KDM5B Promotes PDAC Cells Stemness and proliferation.** (A) Western blot analysis of the expression of KDM5B in the indicated human pancreatic cancer cell lines. GAPDH was used as a loading control. (B) Immunoblotting and (C) qRT-PCR to measure KDM5B expression in BxPC-3 and CFPAC-1 cells transfected with shCtr and/or shKDM5B. (D) Immunoblotting and (E) qRT-PCR to detect KDM5B expression in Capan-1 cells transfected with Vec and/or KDM5B. The expression levels of CSC markers, including CD133, EpCAM, NANOG and SOX2 were examined in (F) shKDM5B-transfected BxPC-3 and CFPAC-1 cells and (G) KDM5B overexpression plasmid-transfected Capan-1 cells by qRT-PCR .

**
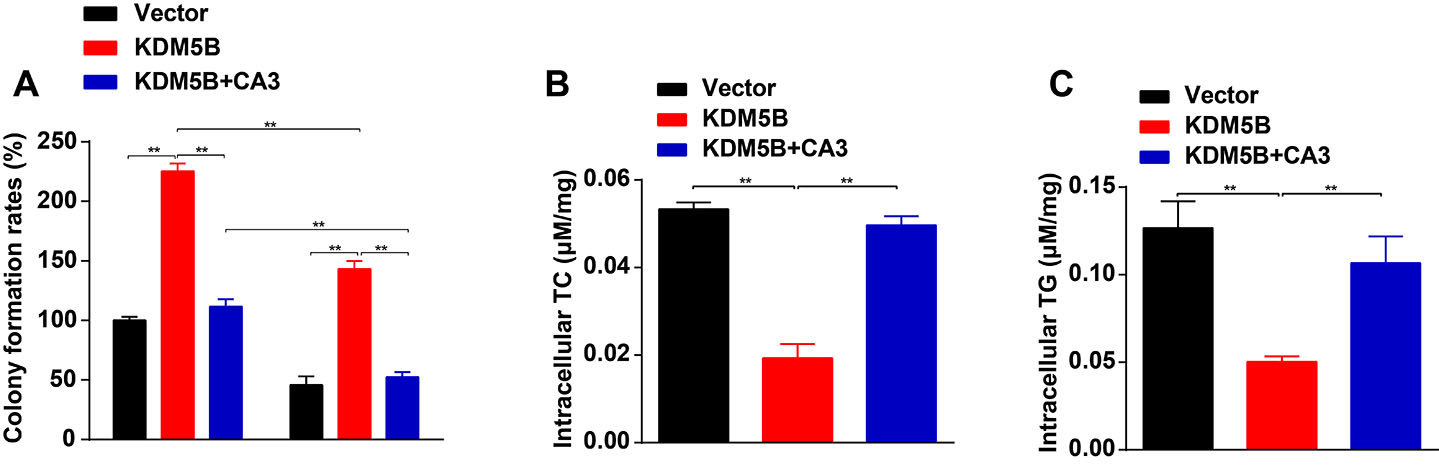
**

**Fig 3S related to Fig 4.** (A) Colony formation of KDM5B overexpressed Capan-1 after CA3 treatment. The quantitative detection of cholesterol (B) and triglyceride (C) in KDM5B overexpressed Capan-1 after CA3 treatment.

**
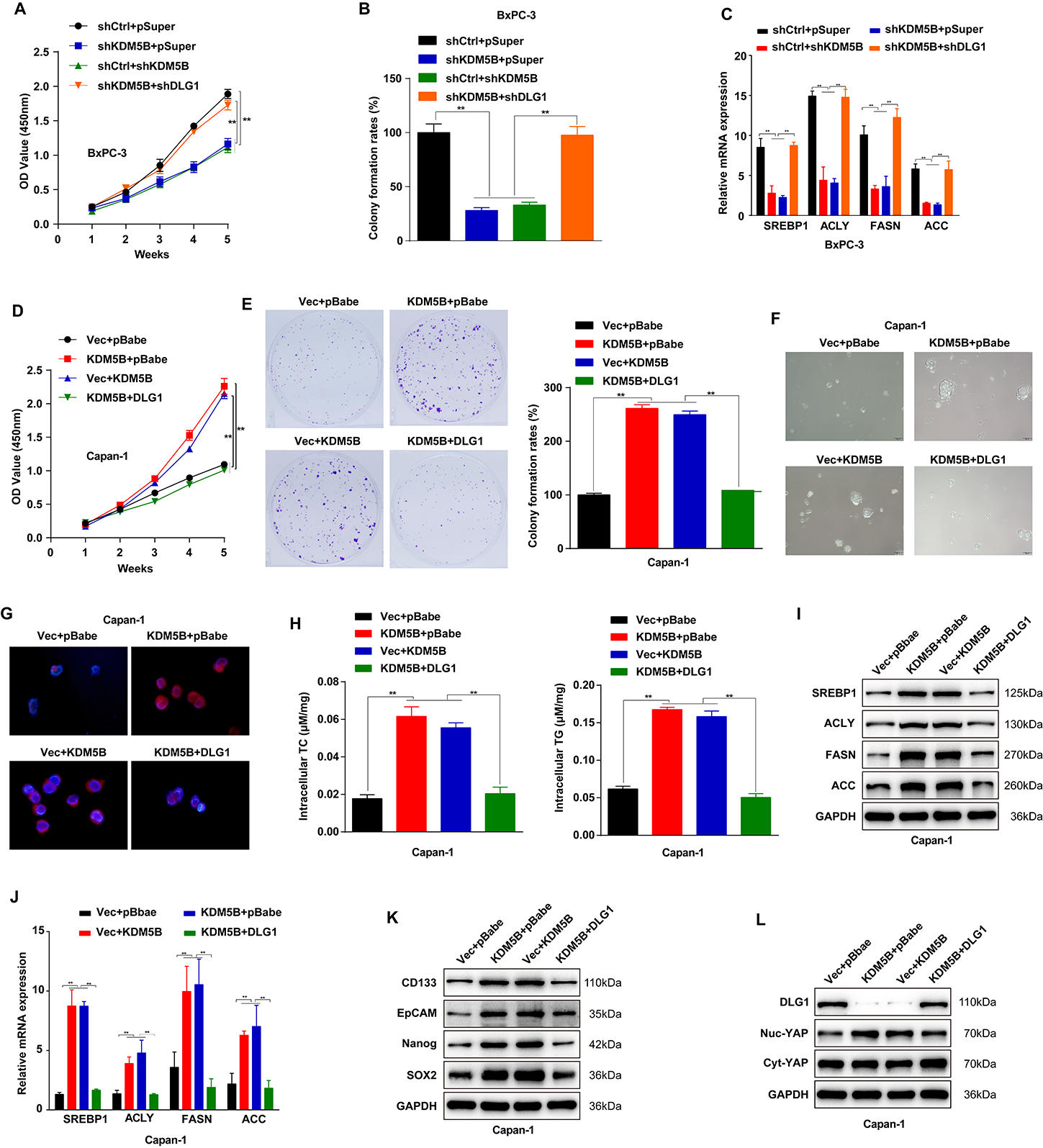
**

**Fig 4S related to Fig 5. DLG1 is a mediator for KDM5B-induced malignant phenotype in PDAC cells via Hippo pathway.** (A) Cell proliferation analysis using CCK8 in KDM5B silencing BxPC-3 cells transfected with or without shDLG1.(B) Cell proliferation analysis using CCK8 in KDM5B overexpressed Capan-1 cells transfected with or without DLG1.(C) RT-qPCR analysis detects the expression of de novo lipogenesis in the indicated cells as described in (A and B). Capan-1 cells transfected with indicated construct and then analyze Cell proliferation (D), clone formation ability (E), cell sphere number (F), neutral lipids by Oil red O staining assay(G), and contents of cholesterol and triglyceride (H). (I) Immunoblotting and (J) qRT-PCR to measure de novo lipogenesis enzymes in the indicated cells as described in (D-H). (K) Expression of stemness markerwere detected by immunoblotting of lysates cells as described in (D-J). (L) YAP protein levels in Cytosol/Nucleus fractions were analyized by immunoblotting in the indicated cells as described in (D-H).

**
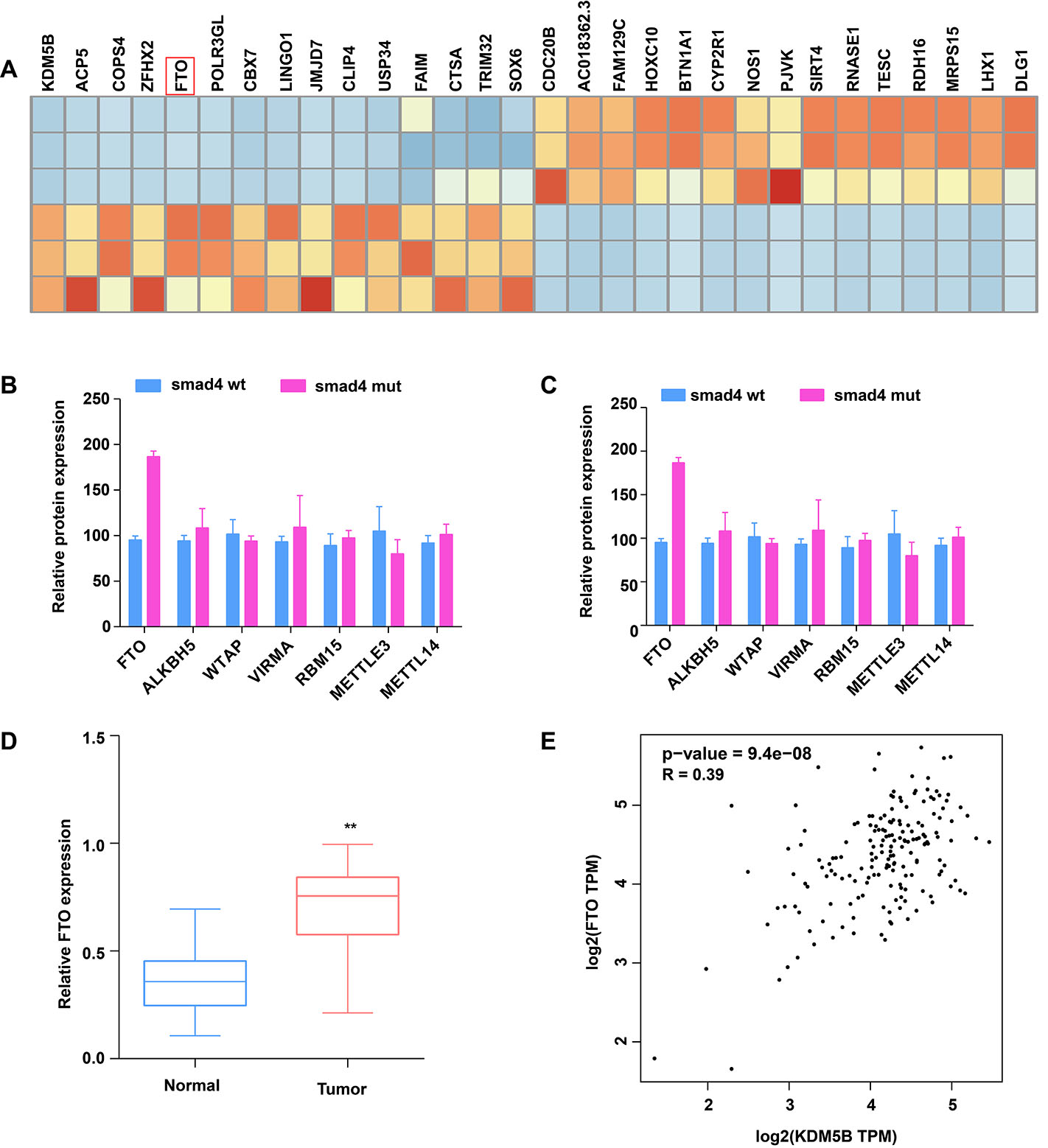
**

**Fig 5S related to Fig 7.** (A) RNA-seq assay showing part of the genes altered in P3-PDX treated with control or gemcitabine.Immunoblotting (B) and qRT-PCR (C) to detect the expression of m^6^A demethylases and methylases in PDAC with or without SMAD4 loss. (D) qRT-PCR for FTO in sections from human PDAC or paracarcinoma tissue. (E) Scatter plot analysis of correlation between mRNA levels of FTO and KDM5B in PDAC tissues.

**Supplementary Table S1: Cell lines**

| **Name** | **Source** | **Cat #** |
| --- | --- | --- |
| AsPC-1 | ATCC | CRL-1682™ |
| PANC-1 | ATCC | CRL-1469™ |
| SW-1990 | ATCC | CRL-2172™ |
| BxPC-3 | ATCC | CRL-1687™ |
| MIA PaCa-2 | ATCC | CRL-1420™ |
| CFPAC-1 | ATCC | CRL-1918™ |
| Capan-1 | ATCC | HTB-79™ |

**Supplementary Table S2: Primary antibodies for western blot**

| **Reagent or resource** | **Source** | **Cat #** |
| --- | --- | --- |
| Anti-KDM5B-antibody | Abcam | ab181089 |
| Anti-Smad4-antibody | Abcam | ab40759 |
| Anti-CD133-antibody | Abcam | ab19898 |
| Anti-Nanog-antibody | thermofisher | PA1-097 |
| Anti-SOX2-antibody | thermofisher | PA1-094 |
| Anti-EpCAM-antibody | Abcam | ab71916 |
| Anti-Ki67-antibody | Abcam | ab15580 |
| Anti-YAP-antibody | CST | 14074T |
| Anti-OCT4-antibody | Abcam | ab18976 |
| Anti-CD44 (156-3C11) -antibody | CST | 3570 |
| Anti-DLG1-antibody | proteintech | 29292-1-AP |
| Anti-FTO-antibody | CST | 14386 |
| Anti-ALKBH5-antibody | Affinity | DF2585 |
| Anti-METTL3-antibody | Santa Cruz Biotechnology | sc-518180 |
| Anti-METTL14-antibody | Abcam | ab309096 |
| Anti-WTAP-antibody | Abcam | ab195380 |
| Anti-VIRMA-antibody | Abcam | ab271136 |
| Anti-RBM15-antibody | Abcam | ab244374 |
| Anti-YTHDF2-antibody | Abcam | ab220163 |
| Anti-ACC-antibody | CST | 3676 |
| Anti-SREBP1-antibody | Abcam | ab28481 |
| Anti-FASN-antibody | Abcam | Ab128856 |
| Anti-ACLY-antibody | Abcam | ab40793 |
| Anti-GAPDH-antibody | proteintech | 60004-1-Ig |
