# Supplementary material for: KDM5B promotes SMAD4 loss-driven drug resistance through activating DLG1/YAP to induce lipid accumulation in pancreatic ductal adenocarcinoma
Source: Cell Death Discov. 2024 May 24;10:252. doi: 10.1038/s41420-024-02020-4 (PMC11126577; doi:10.1038/s41420-024-02020-4)
Supplement: Supplementary file 2 — Unprocessed WB images.pdf [file 41420_2024_2020_MOESM2_ESM.pdf]

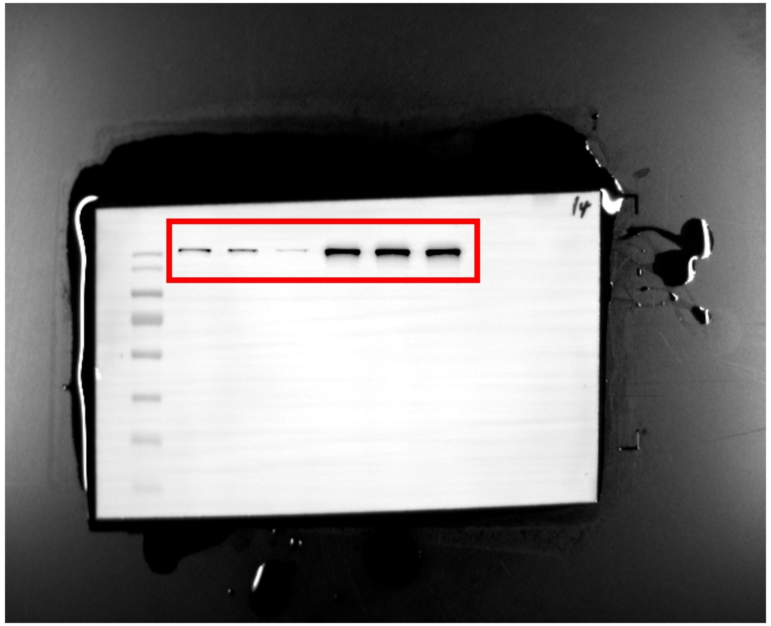

Fig1D-KDM5B-P2

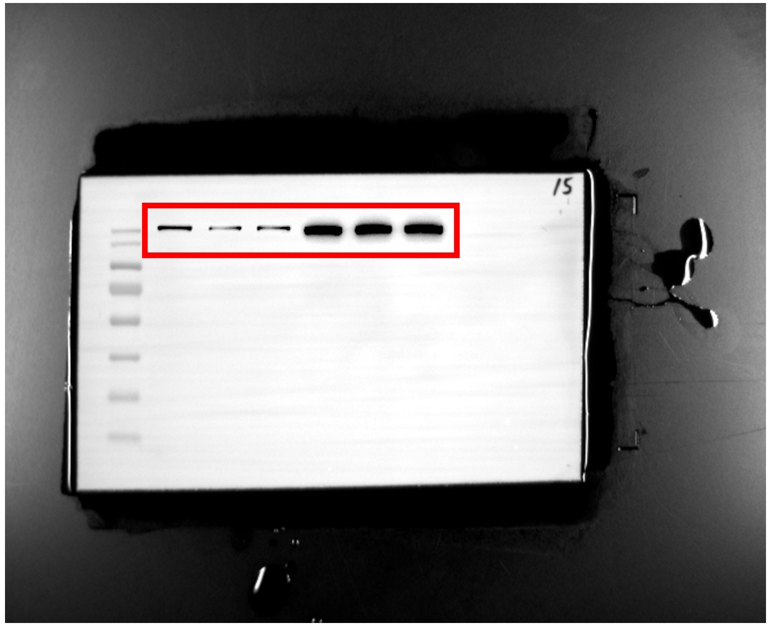

Fig1D-KDM5B-P3

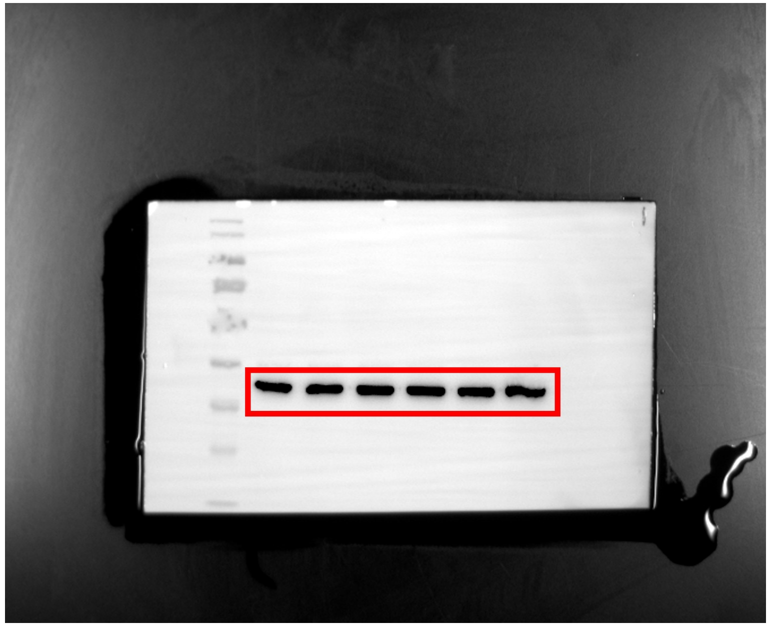

Fig1D-GAPDH-P2

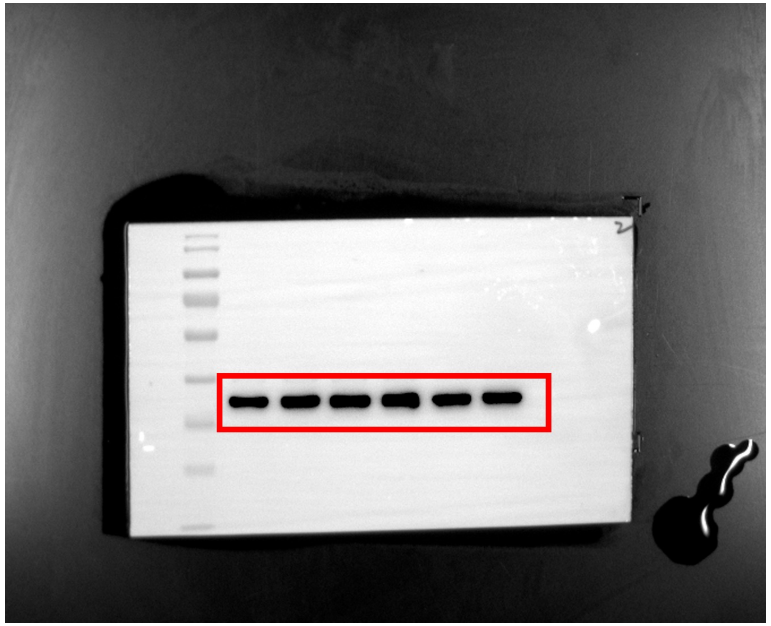

Fig1D-GAPDH-P3

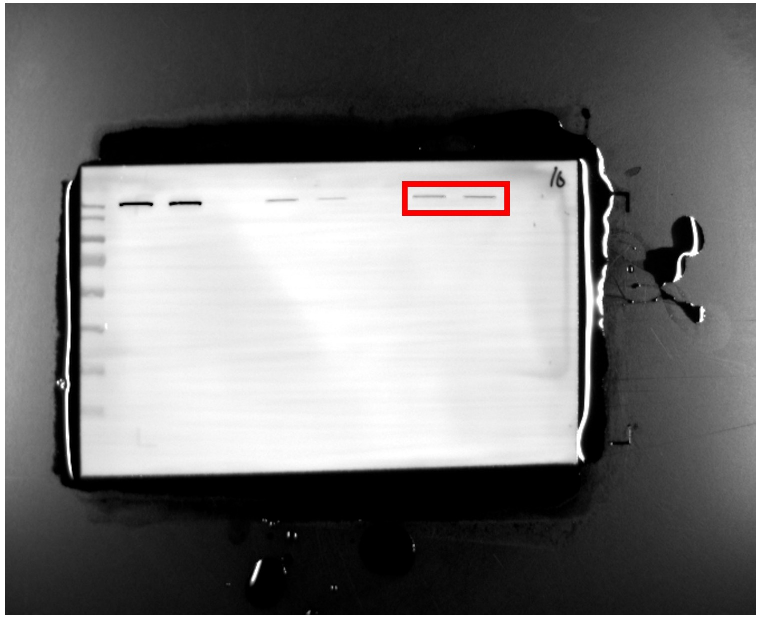
Fig1F-KDM5B-1

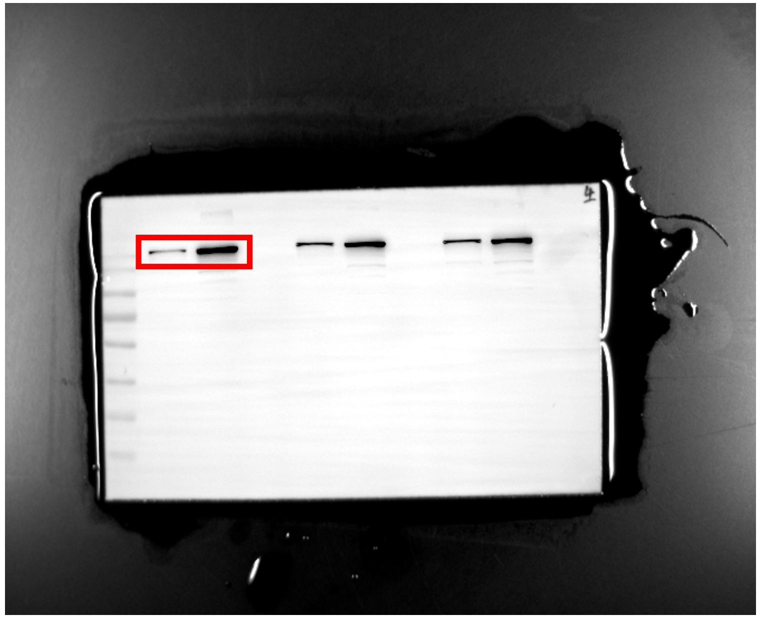
Fig1F-KDM5B-2

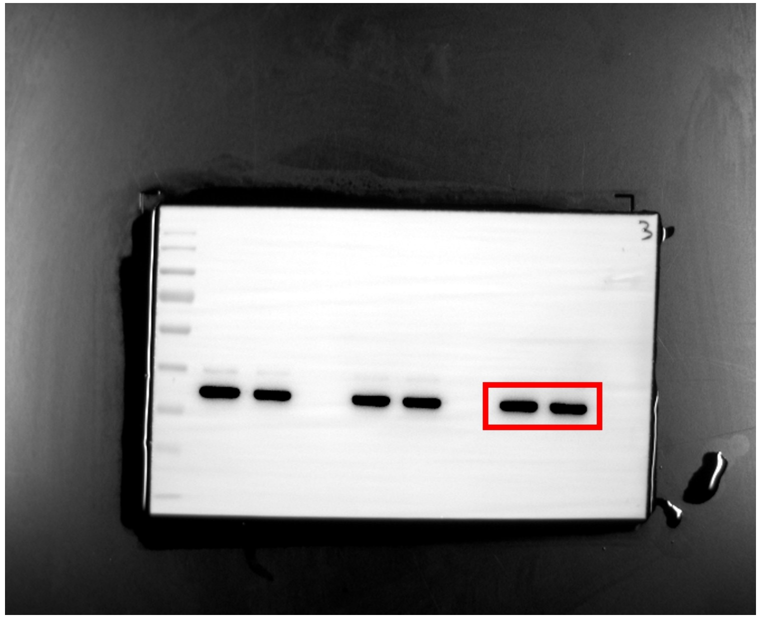
Fig1F-GAPDH-1

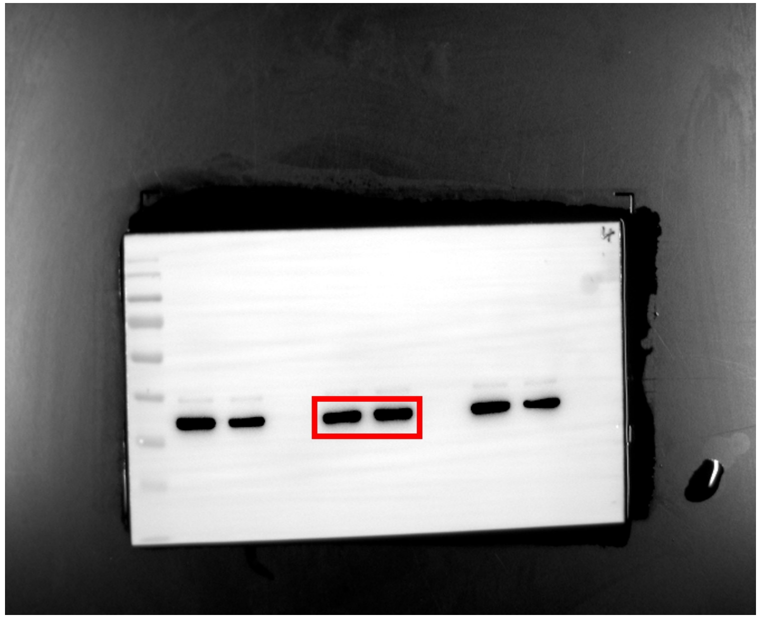
Fig1F-GAPDH-2

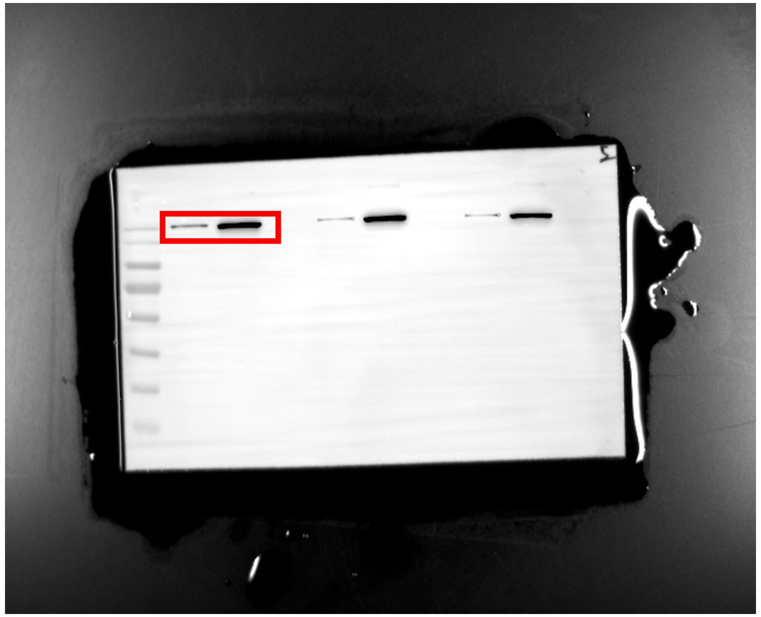

Fig1F-KDM5B-3

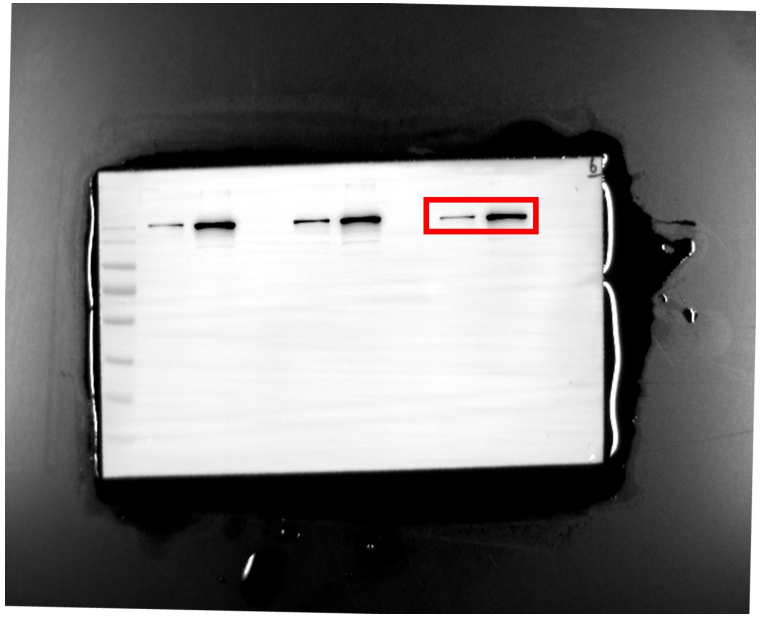

Fig1F-KDM5B-4

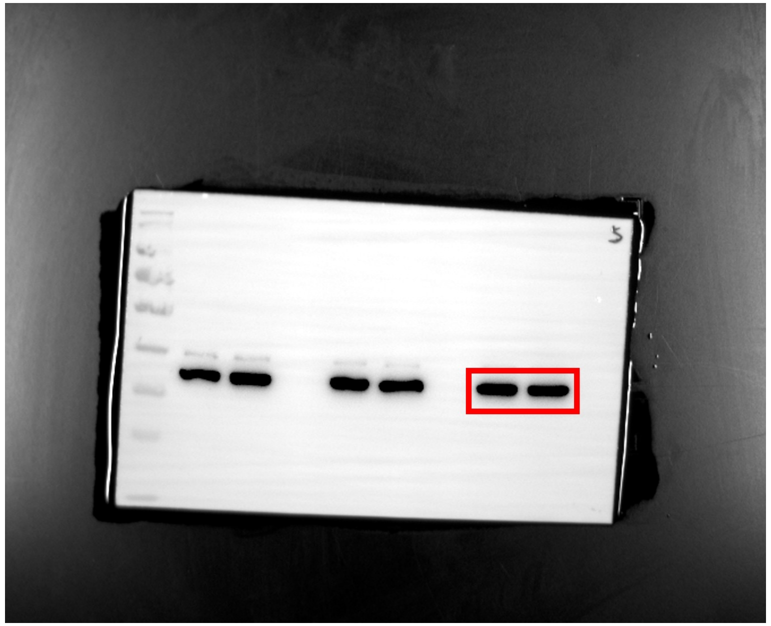

Fig1F-GAPDH-3

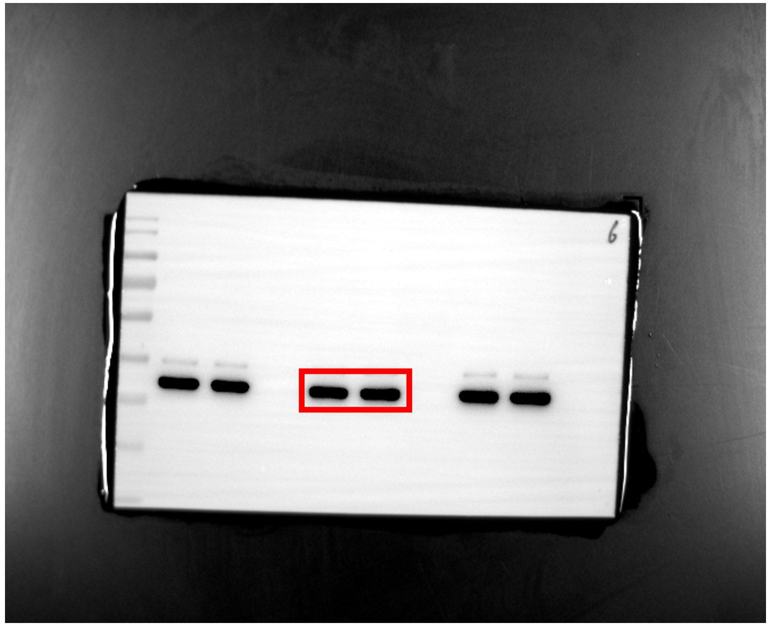

Fig1F-GAPDH-4

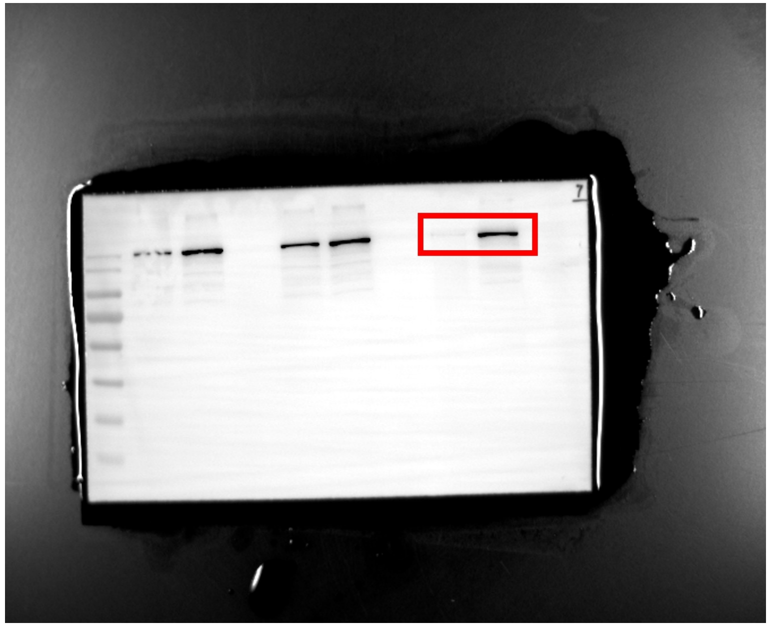

Fig1F-KDM5B-5

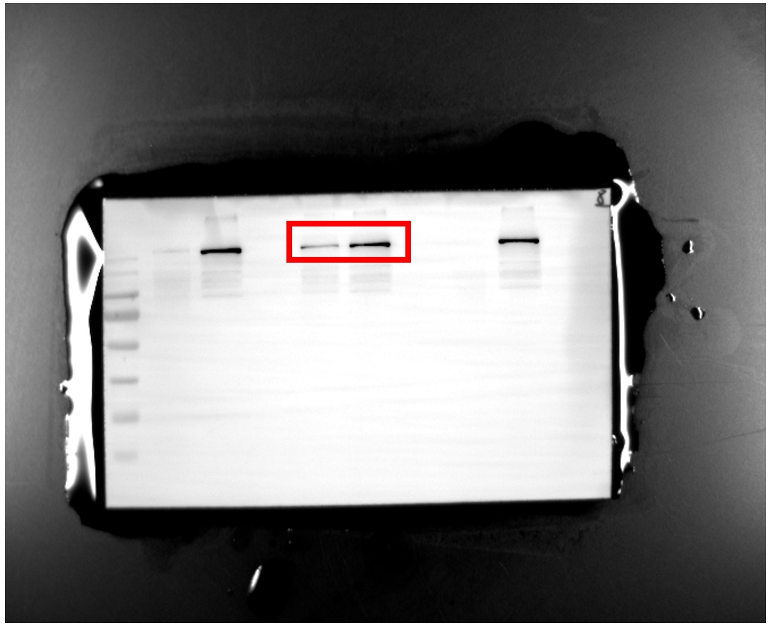

Fig1F-KDM5B-6

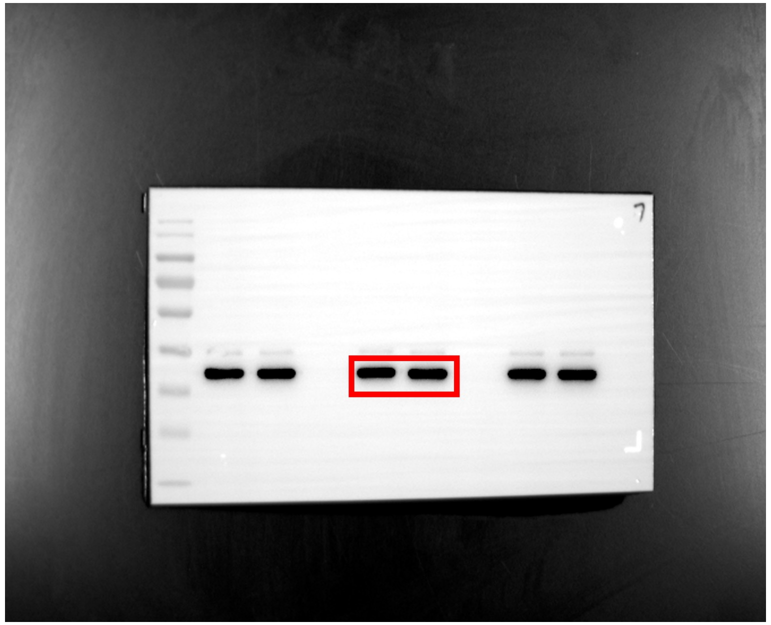

Fig1F-GAPDH-5

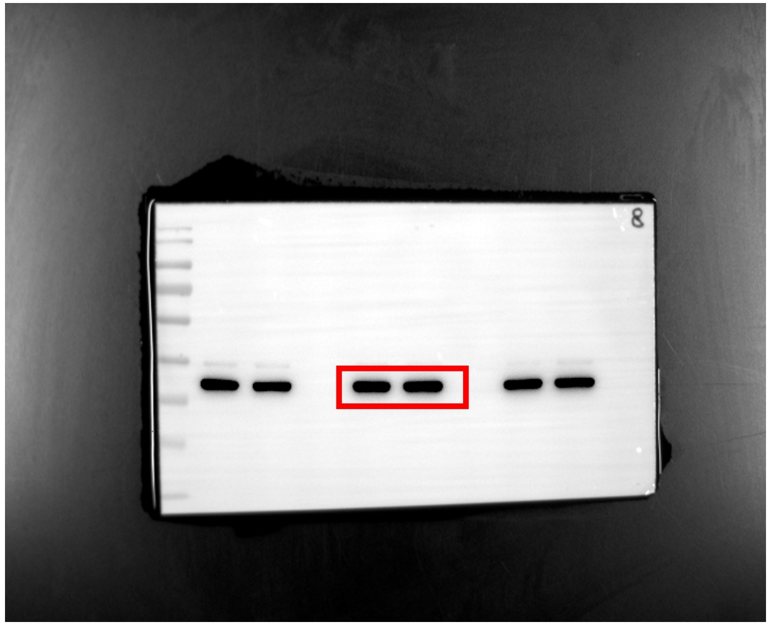

Fig1F-GAPDH-6

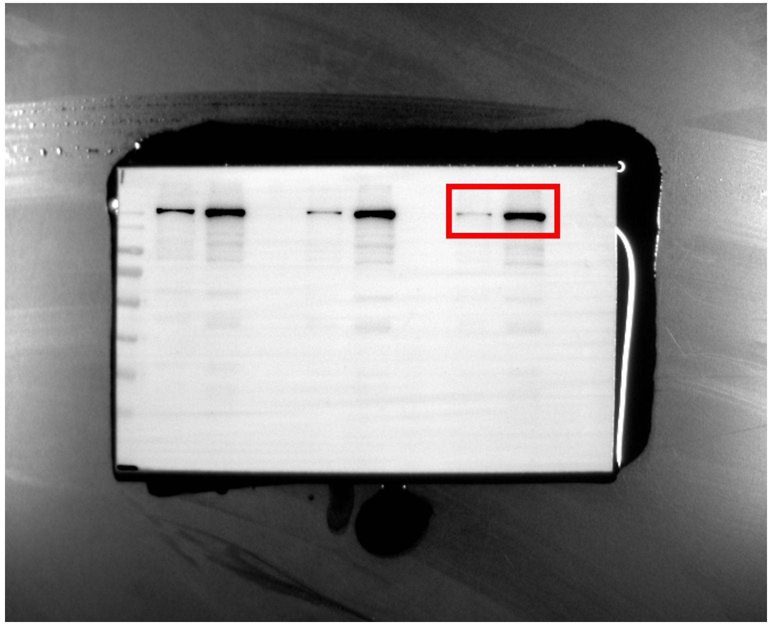

Fig1F-KDM5B-7

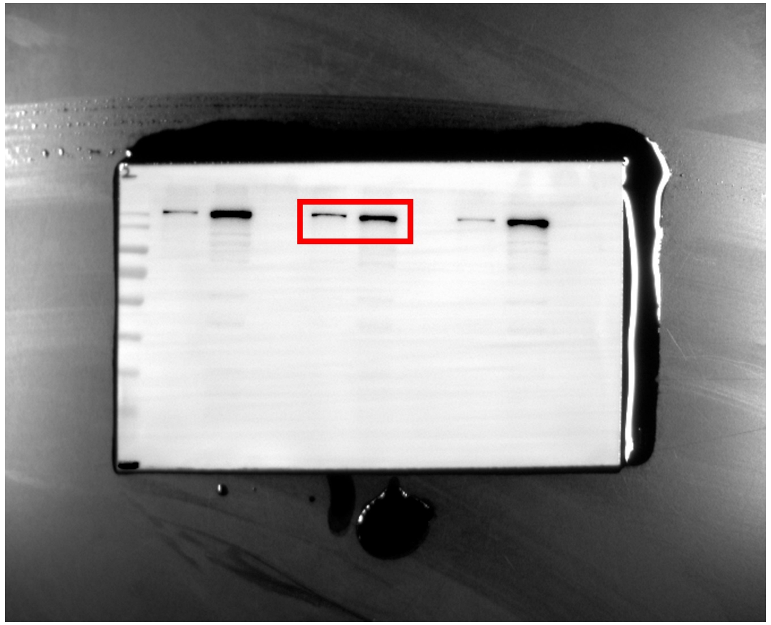

Fig1F-KDM5B-8

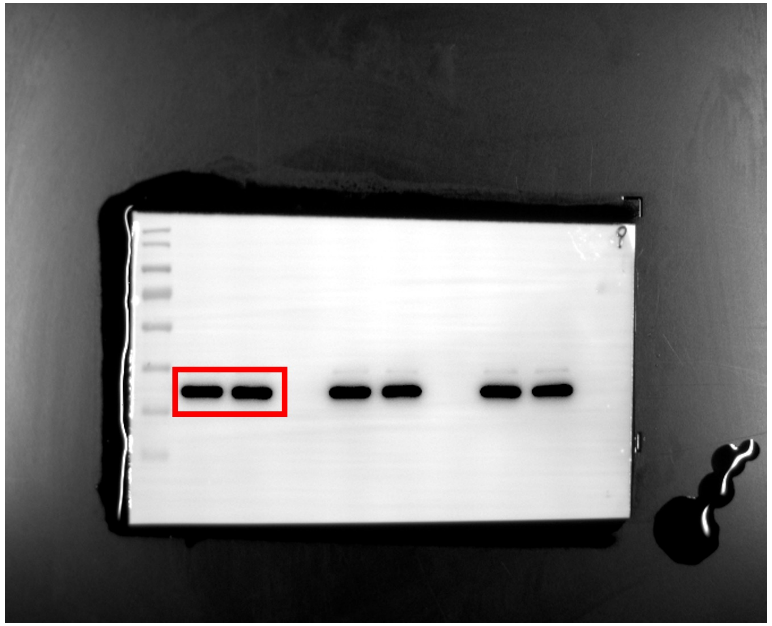

Fig1F-GAPDH-7

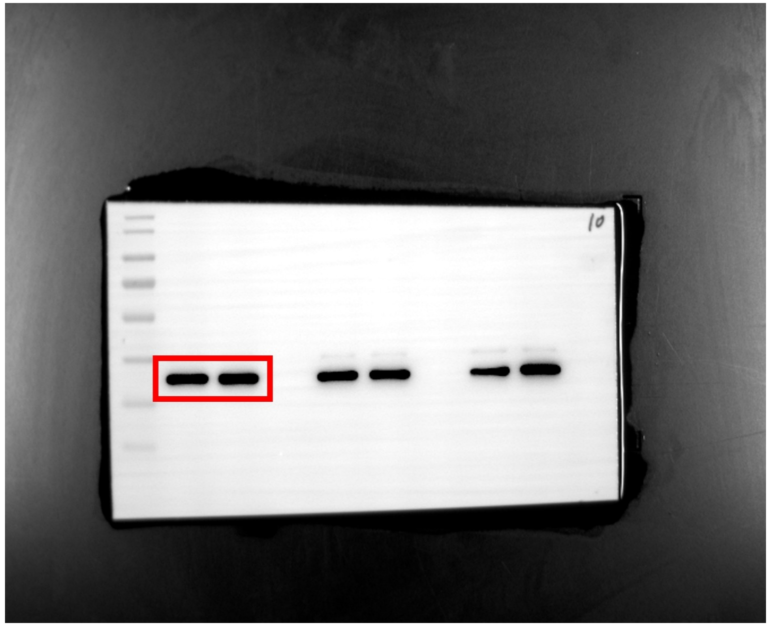

Fig1F-GAPDH-8

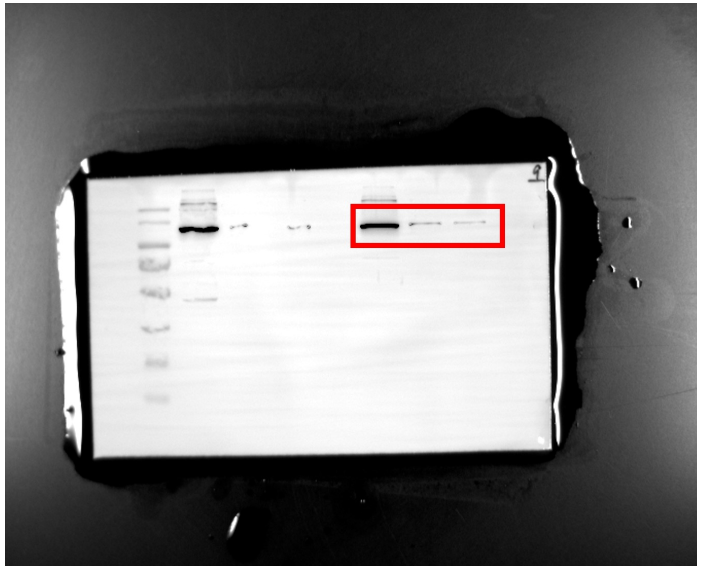

F2E-CD133-BxPC-3

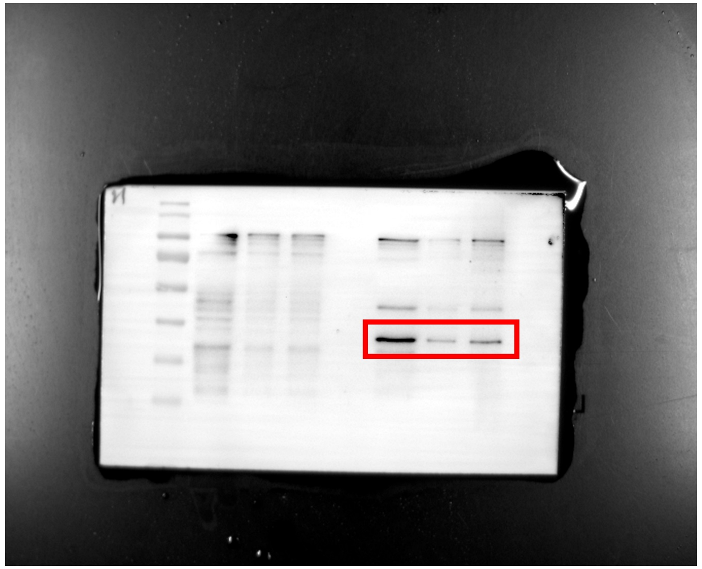

F2E-EpCAM-BxPC-3

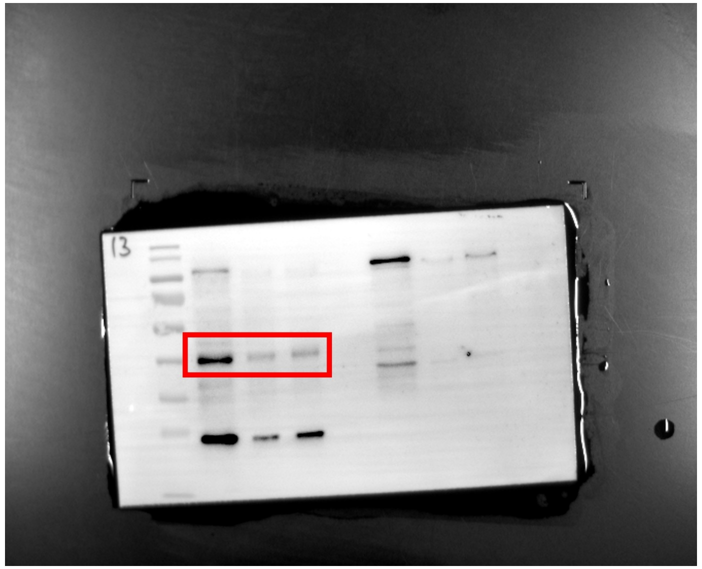

F2E-Nanog-BxPC-3

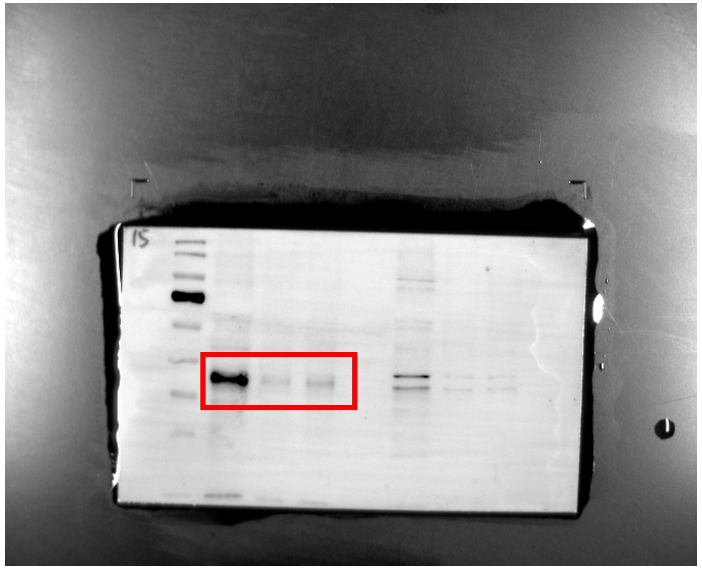

F2E-SOX2-BxPC-3

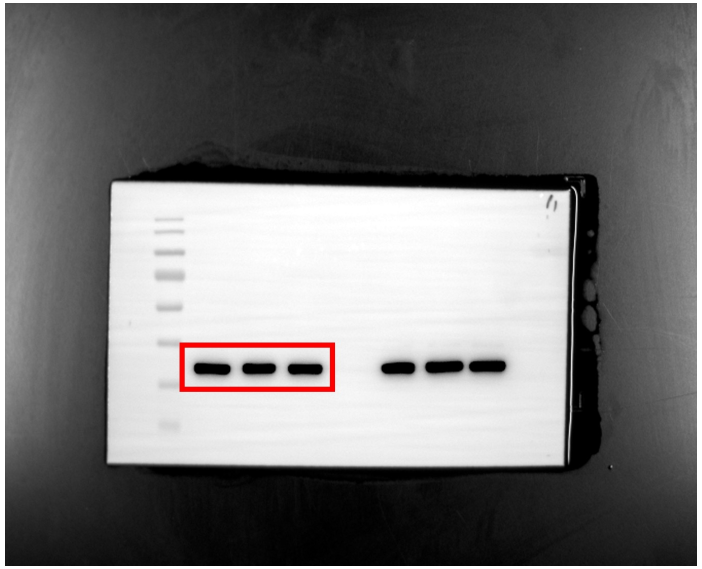

F2E-GAPDH-BxPC-3

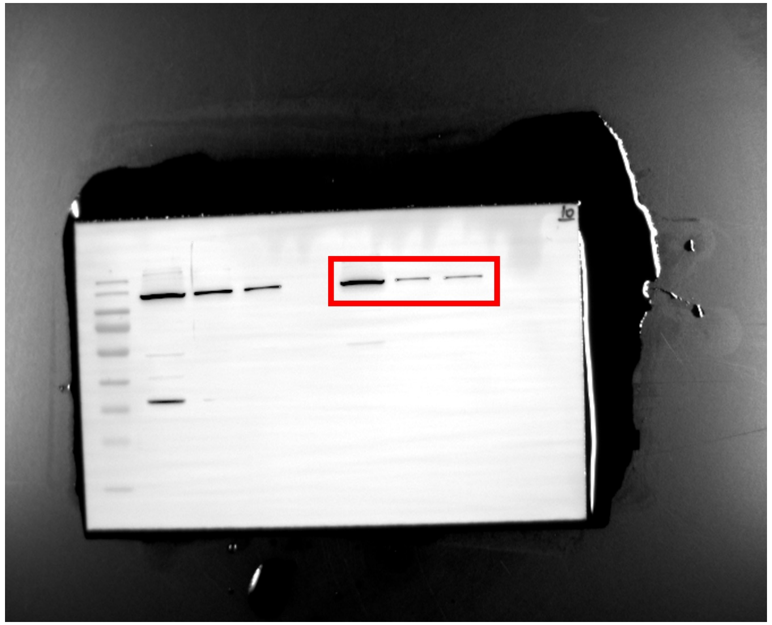

F2E-CD133-CFPAC-1

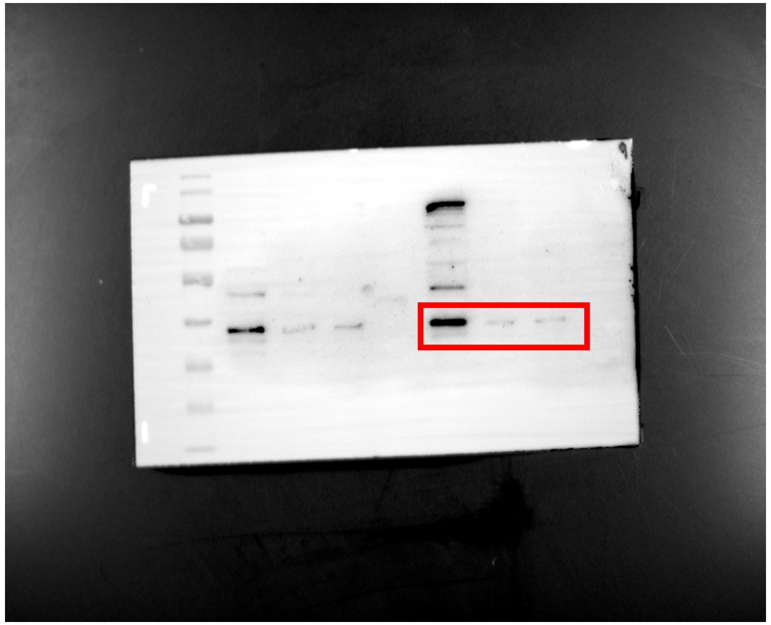

F2E-EpCAM-CFPAC-1

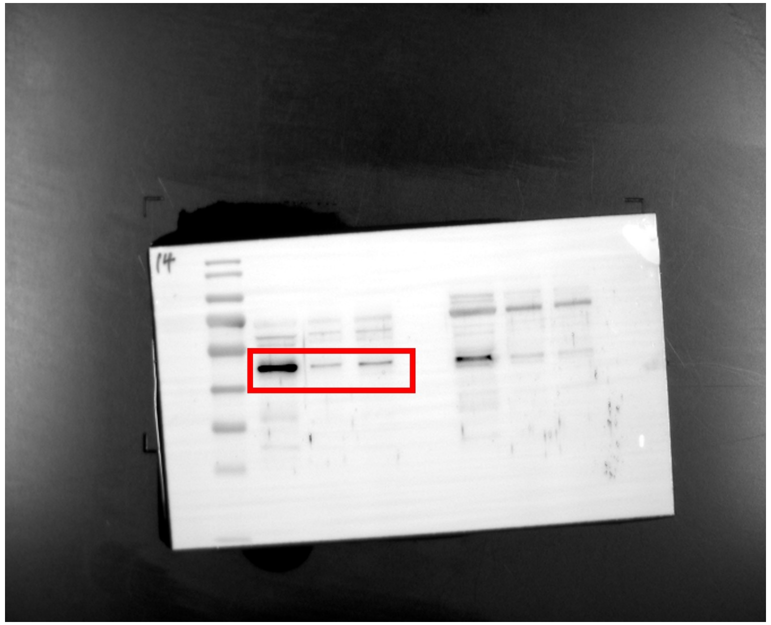

F2E-Nanog-CFPAC-1

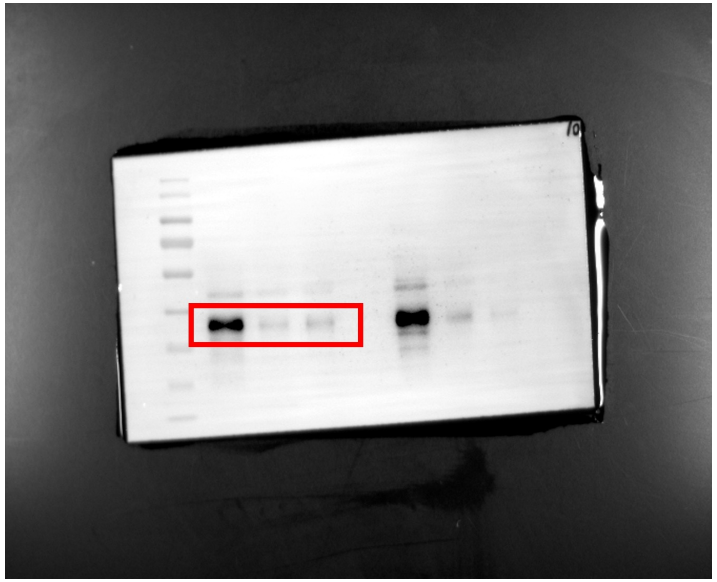

F2E-SOX2-CFPAC-1

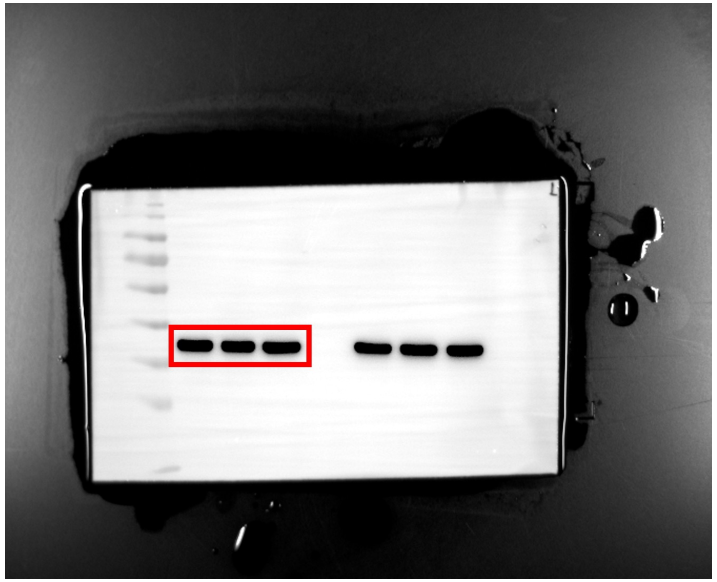

F2E-GAPDH-CFPAC-1

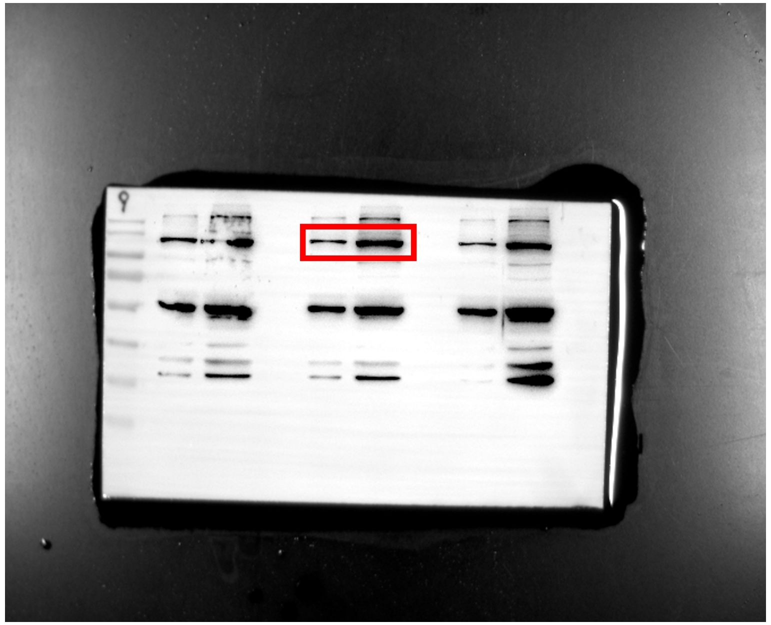

F2F-CD133-Capan-1

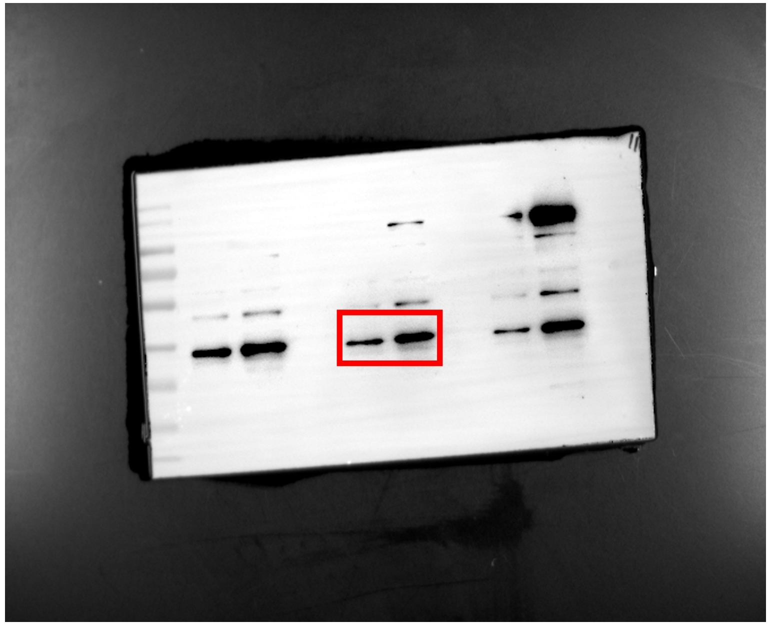

F2F-EpCAM-Capan-1

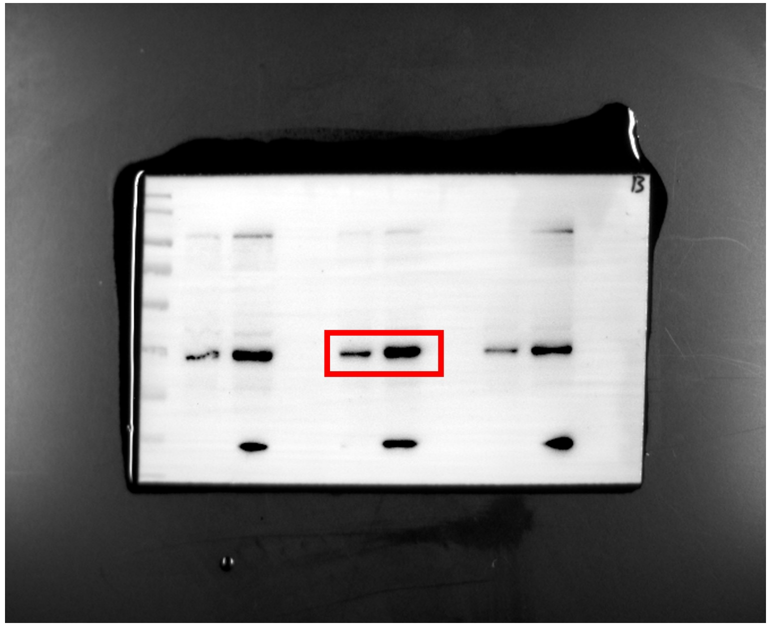

F2F-Nanog-Capan-1

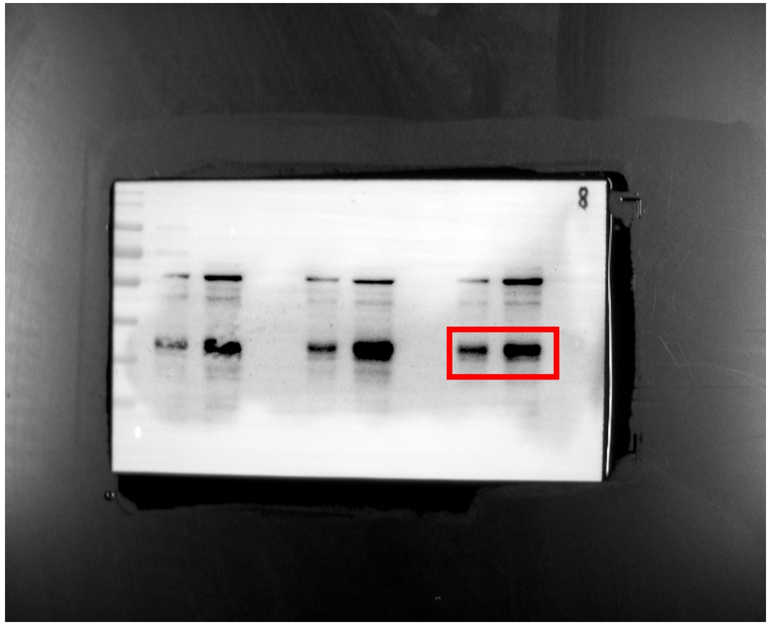

F2F-SOX2-Capan-1

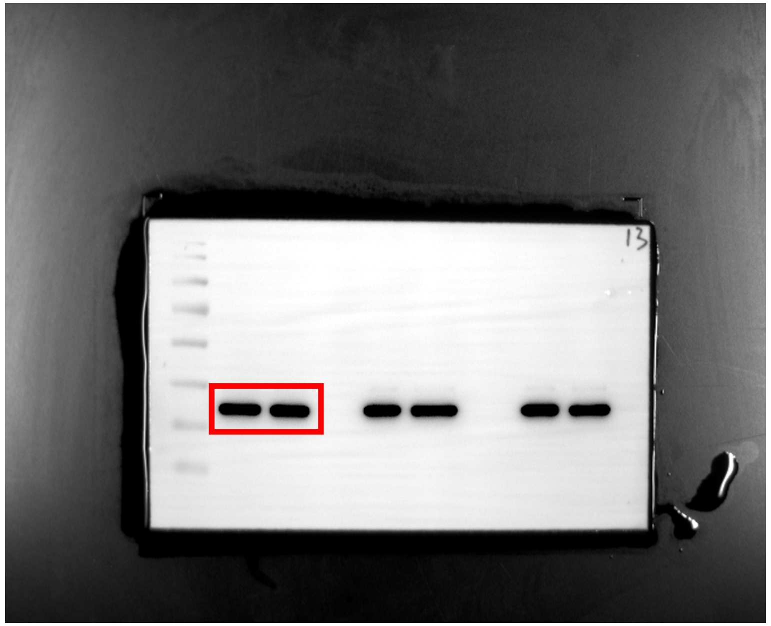

F2F-GAPDH-Capan-1

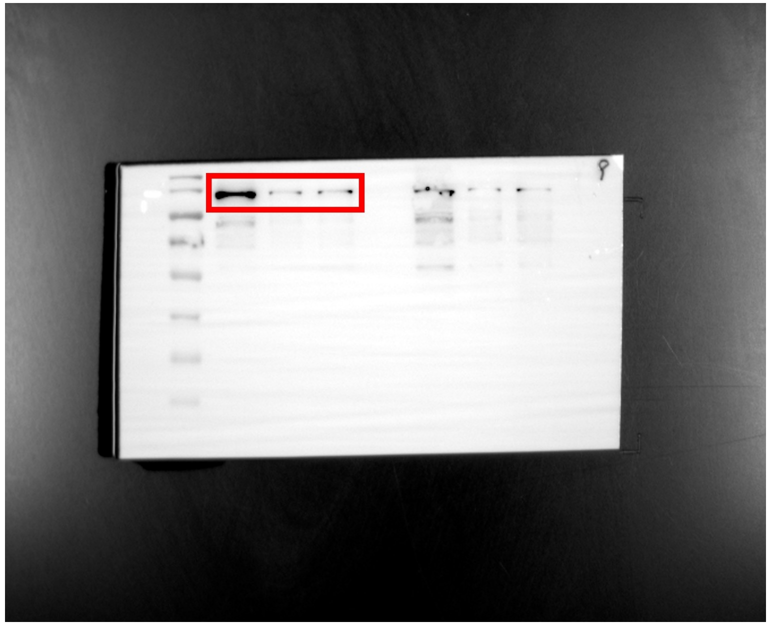

F4G-SREBP1-BxPC-3

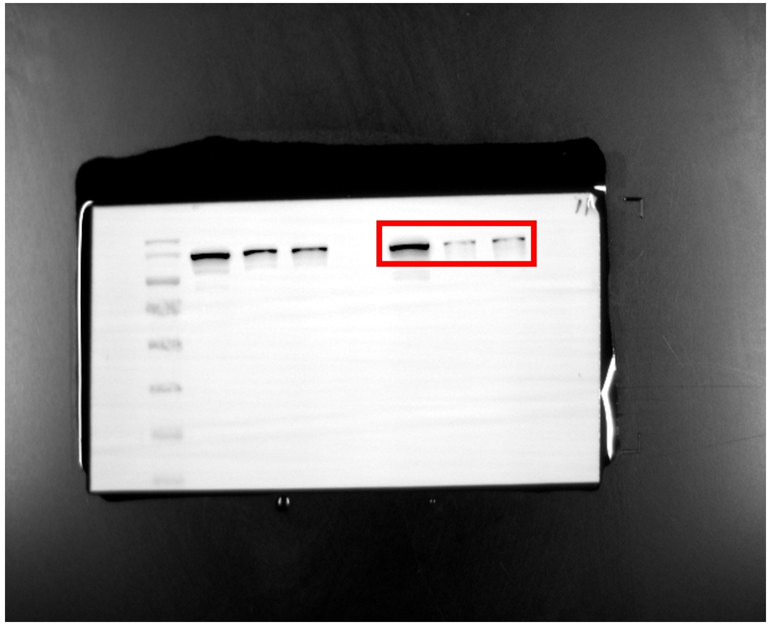

F4G-ACLY-BxPC-3

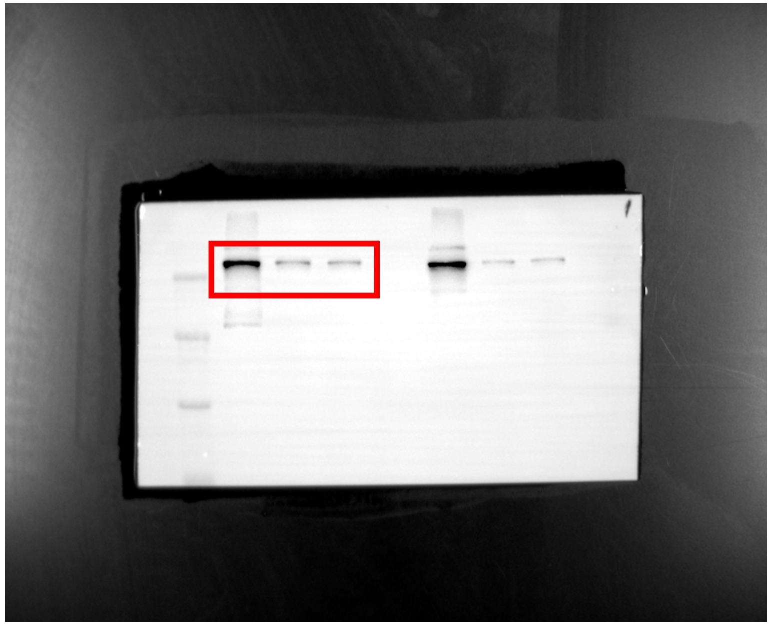

F4G-FASN-BxPC-3

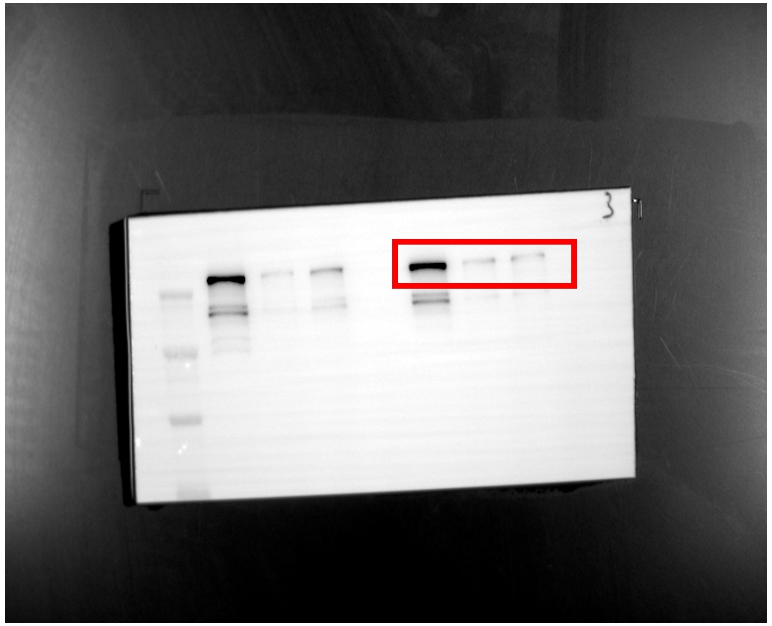

F4G-ACC-BxPC-3

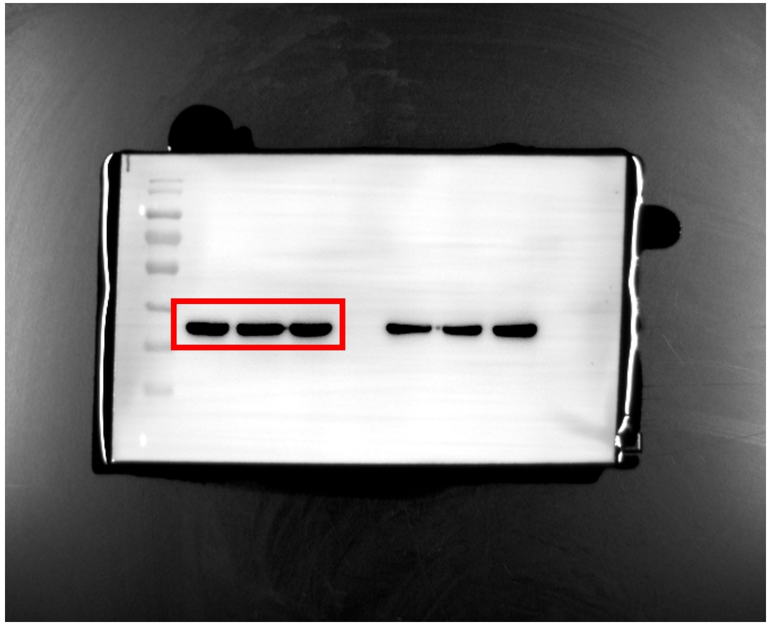

F4G-SREBP1-BxPC-3

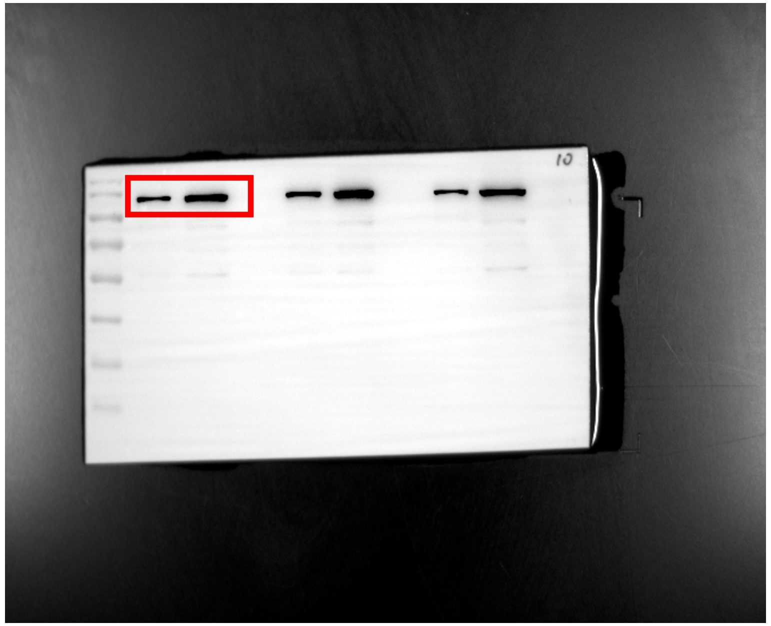

F4G-SREBP1-Capan-1

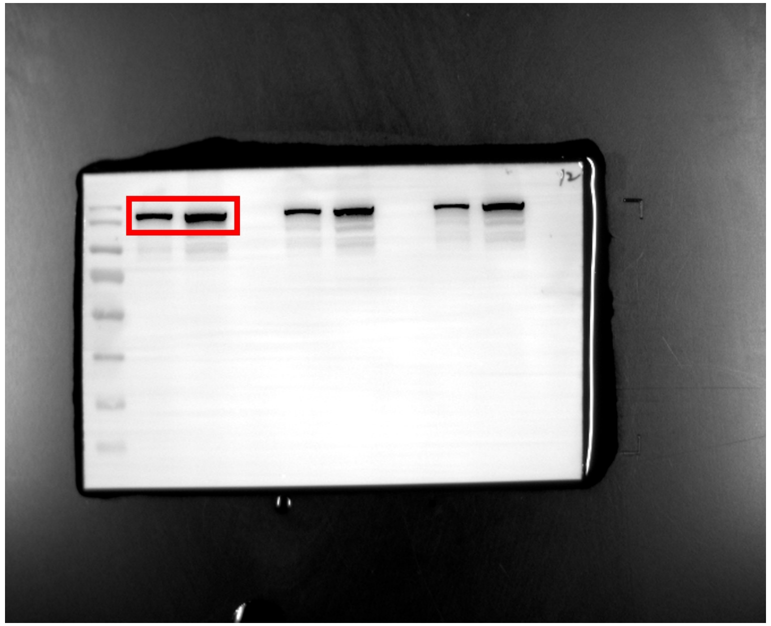

F4G-ACLY-Capan-1

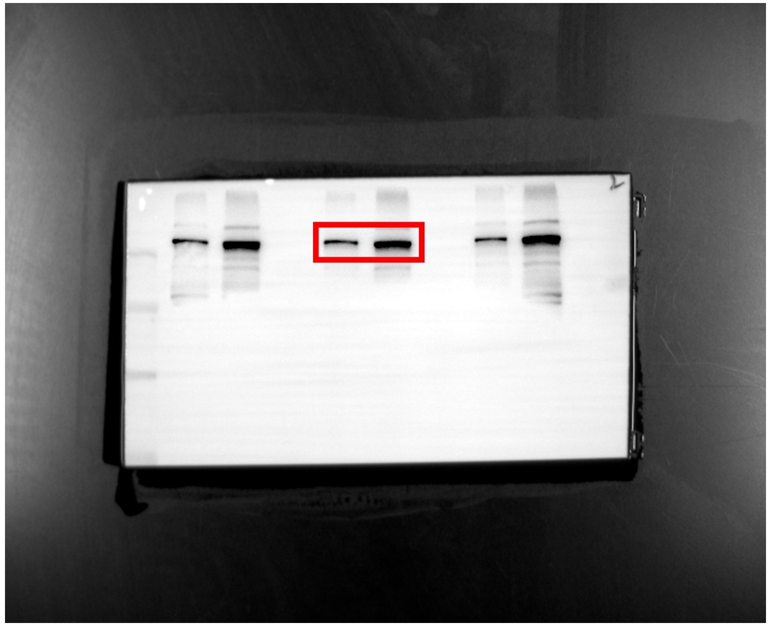

F4G-FASN-Capan-1

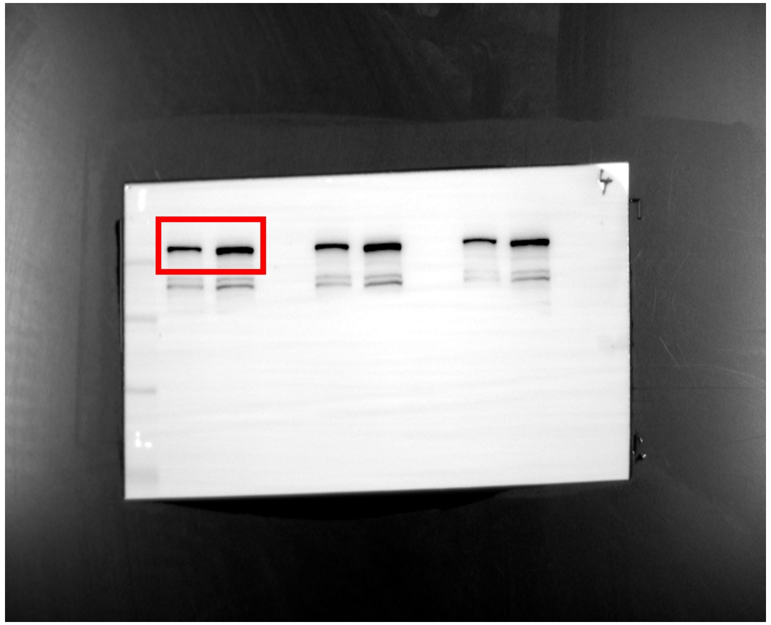

F4G-ACC-Capan-1

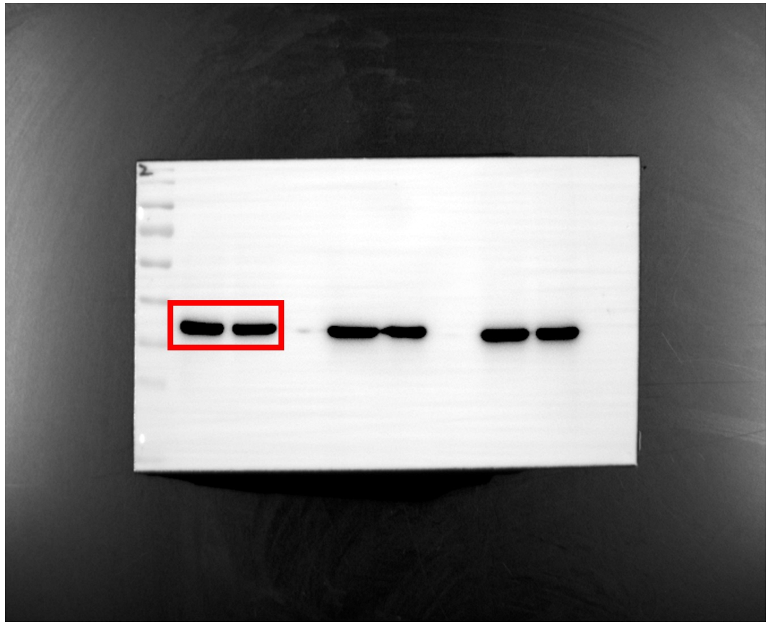

F4G-SREBP1-Capan-1

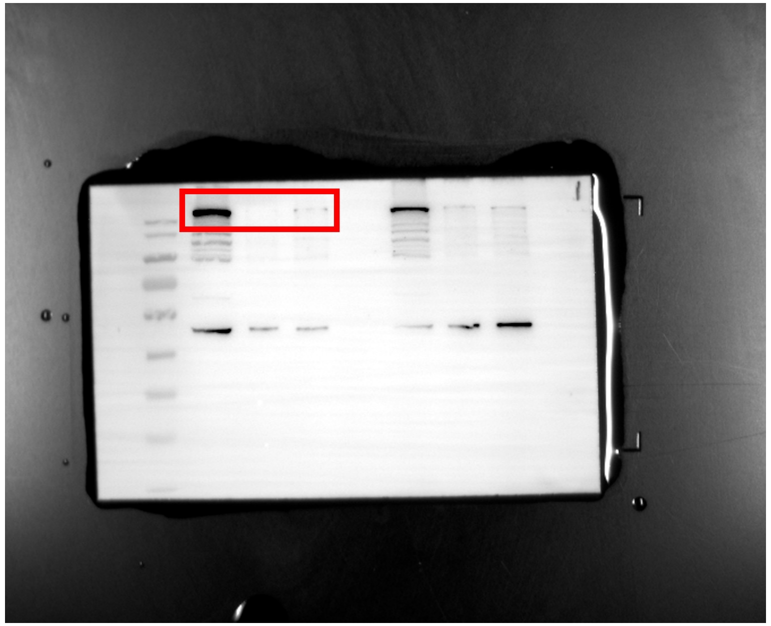

F4H-KDM5B-BxPC-3

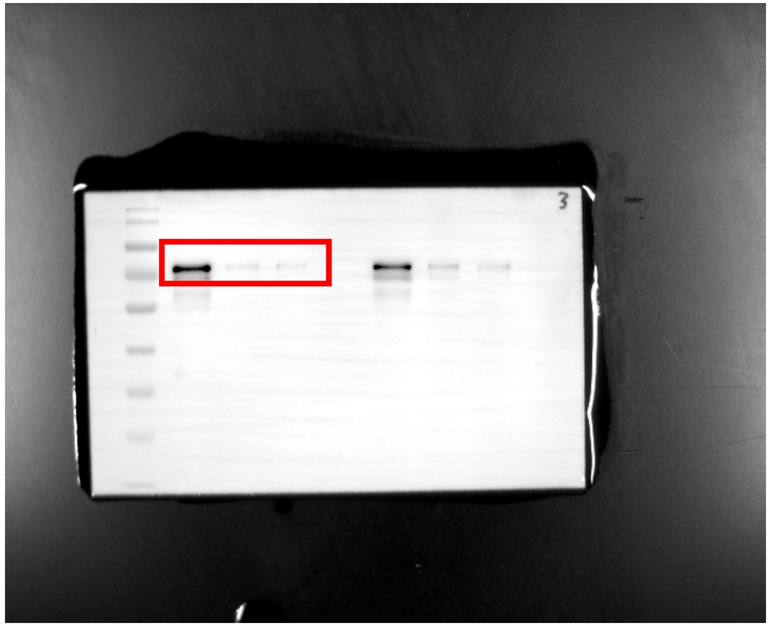

F4H-Nuc-YAP-BxPC-3

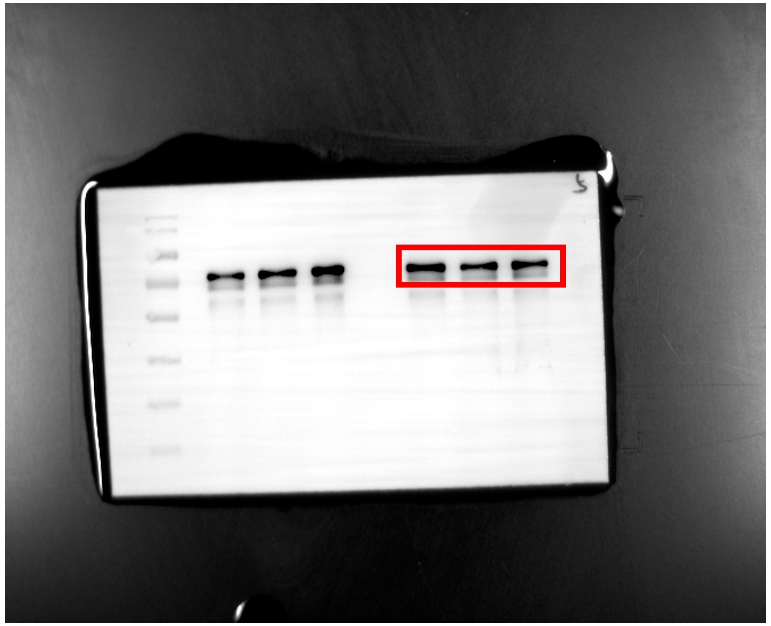

F4H-Cyt-YAP-BxPC-3

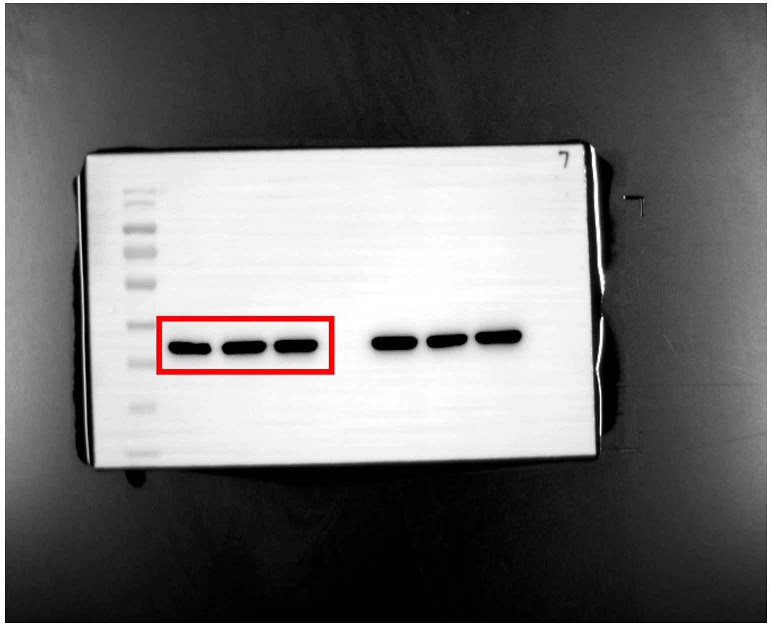

F4H-GAPDH-BxPC-3

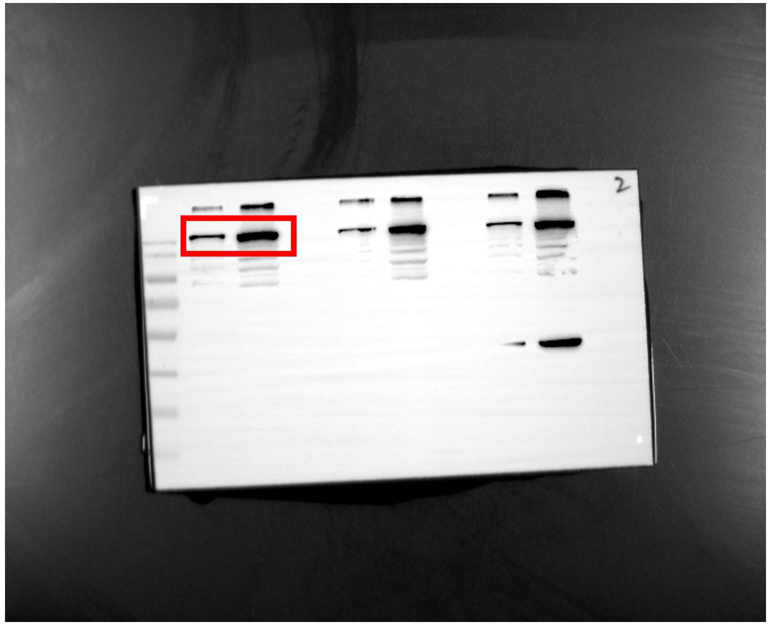
F4H-KDM5B-Capan-1

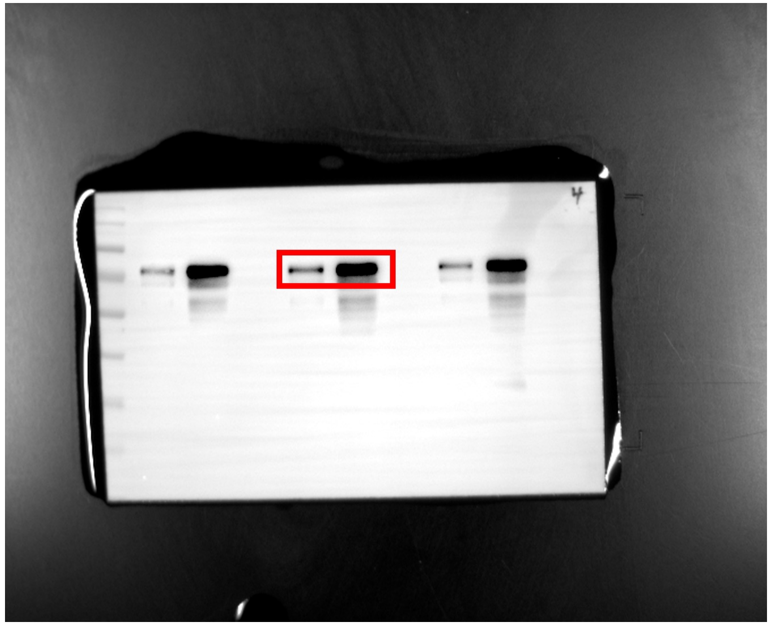
F4H-Nuc-YAP-Capan-1

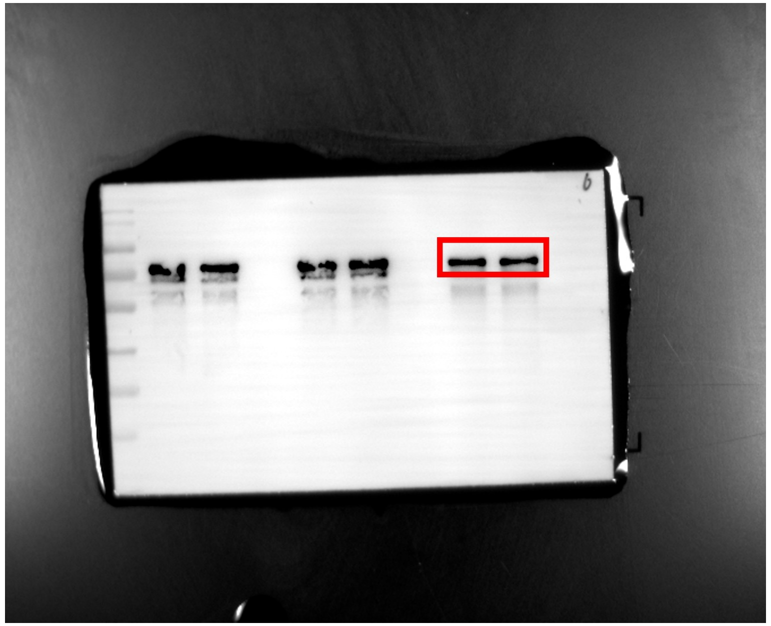
F4H-Cyt-YAP-Capan-1

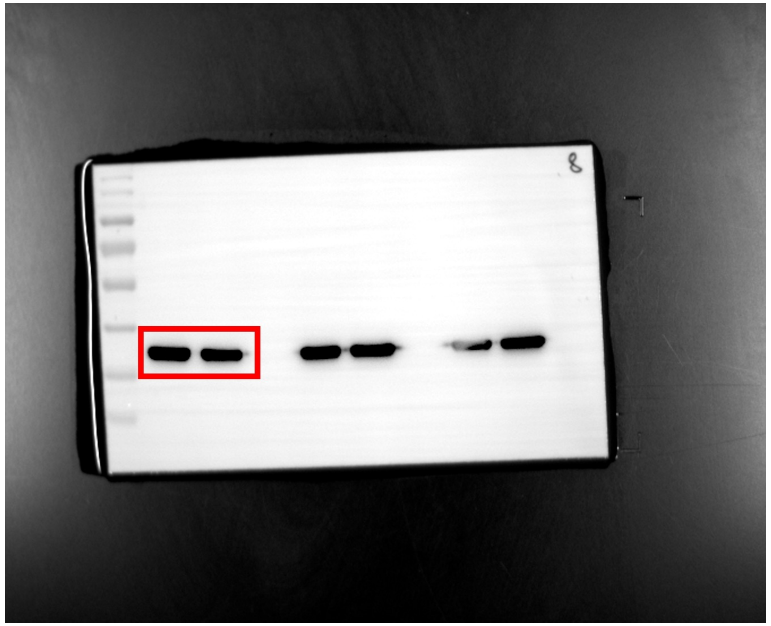
F4H-GAPDH-Capan-1

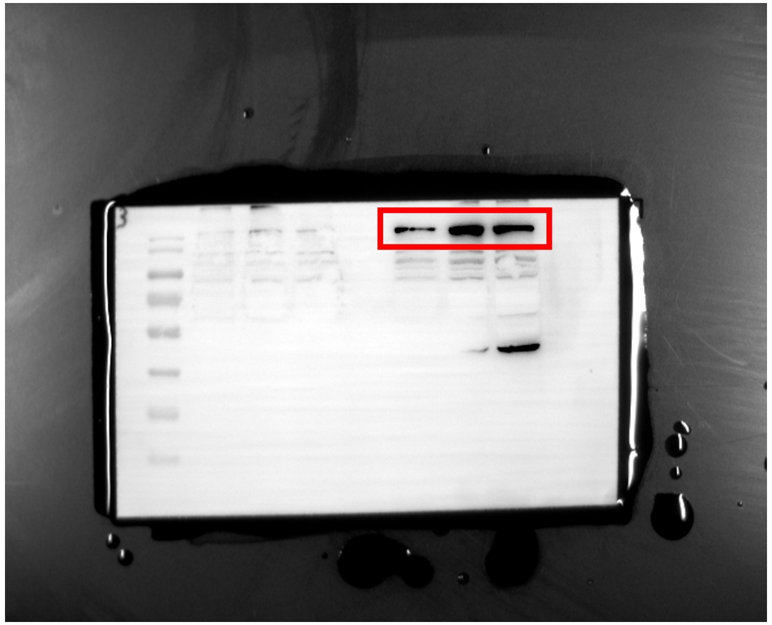

F4I-KDM5B

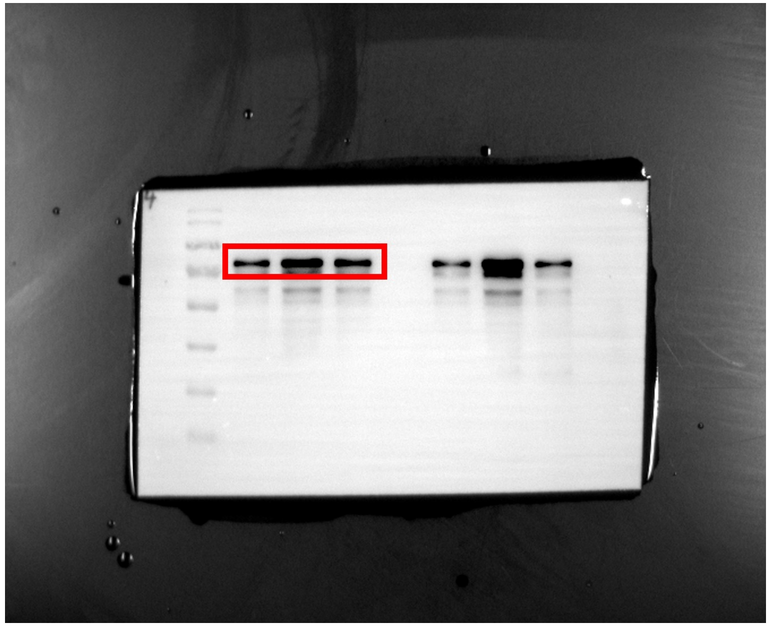

F4I-Nuc-YAP

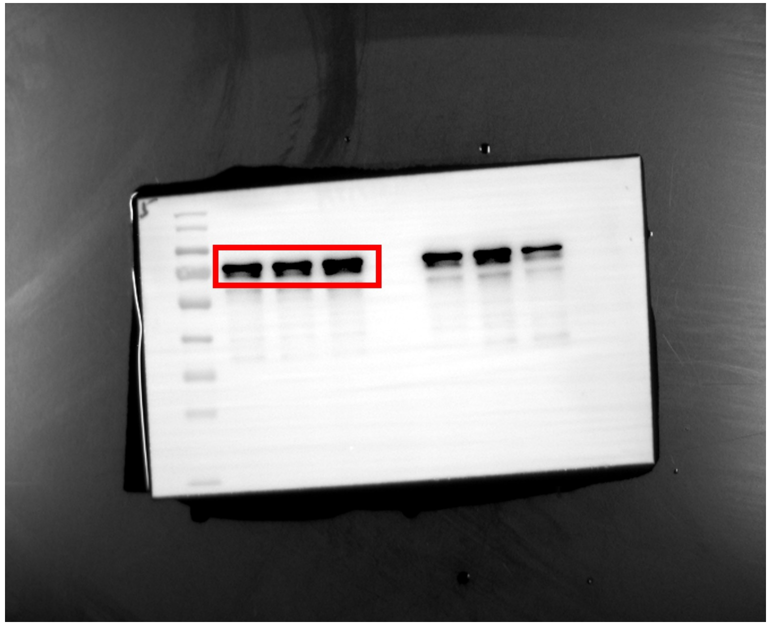

F4I-Cyt-YAP

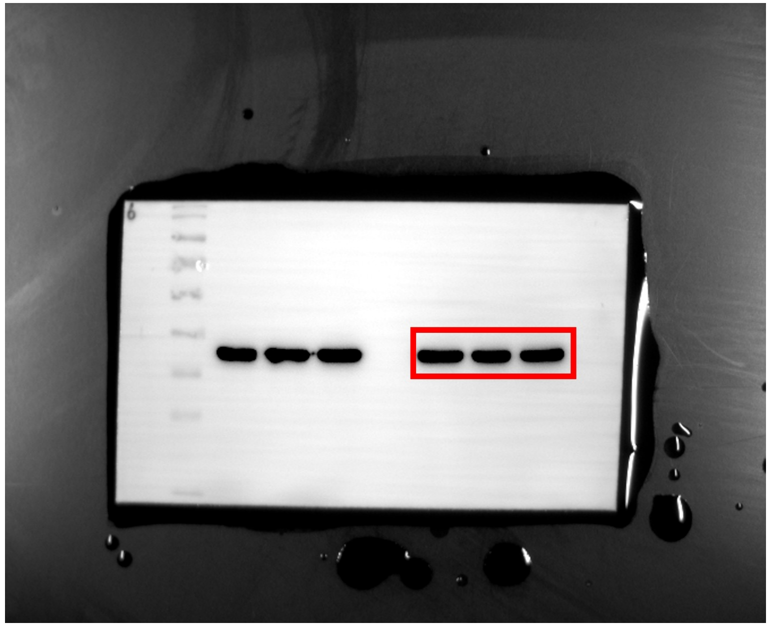

F4I-GAPDH

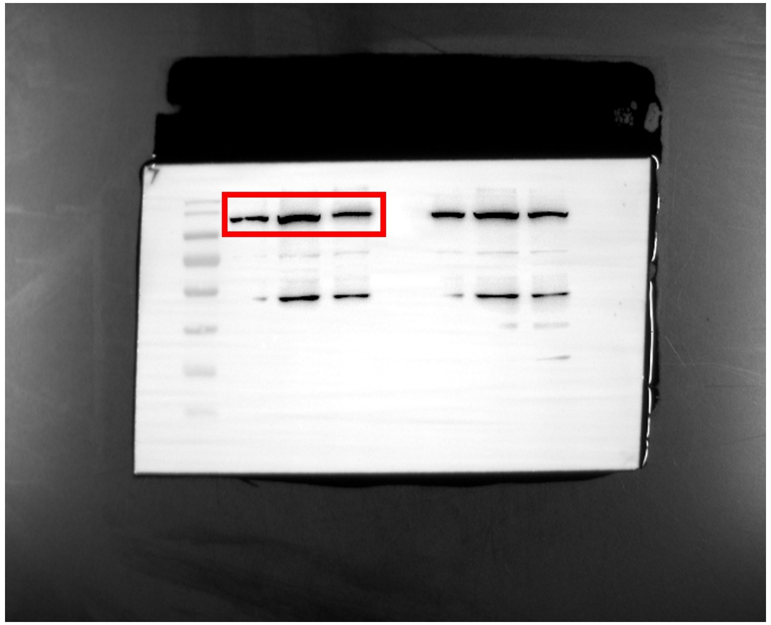
Fig4 M-CD133

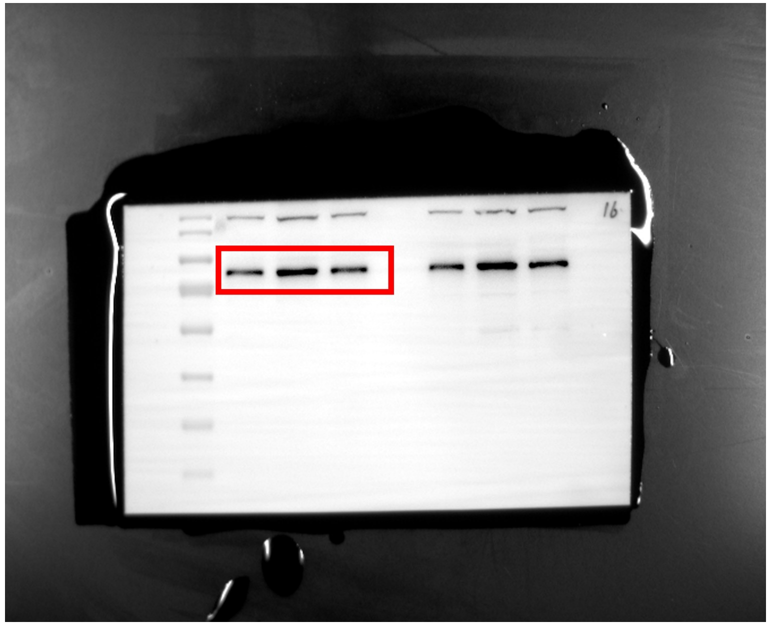
Fig4 M-CD44

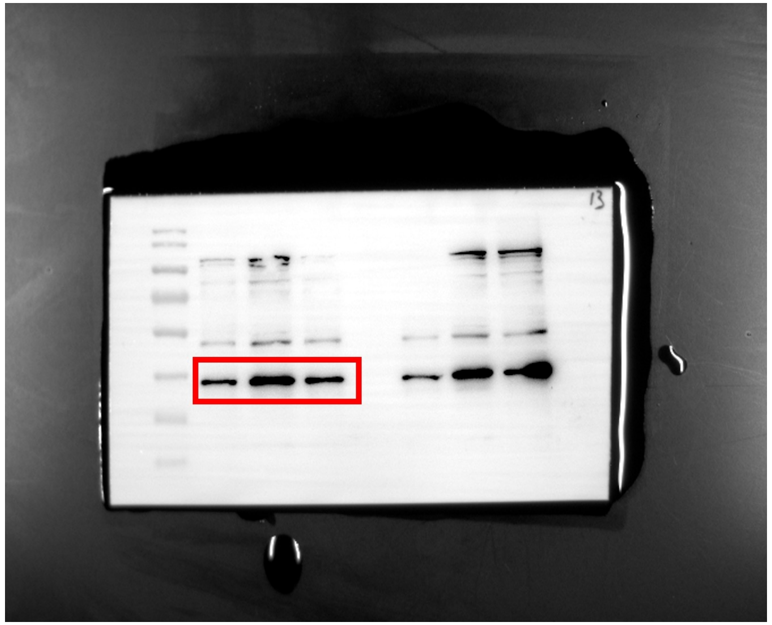
Fig4 M-EpCAM

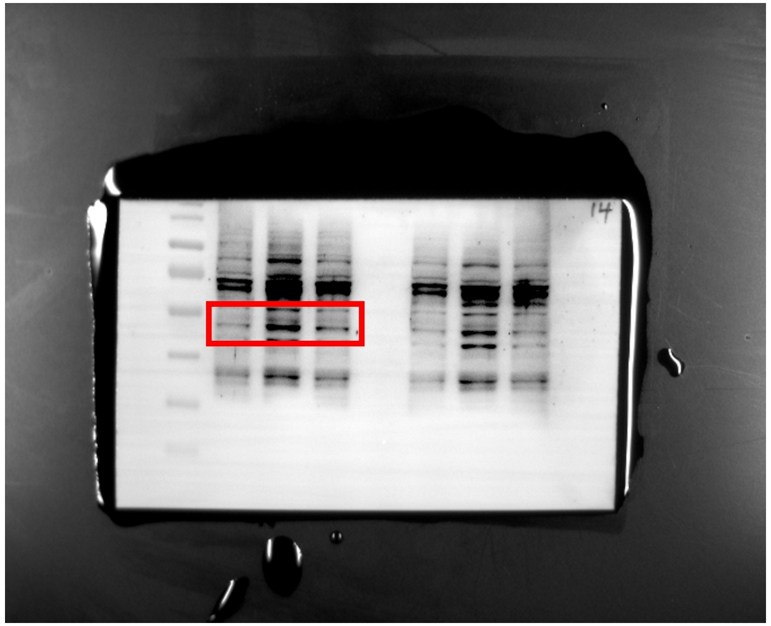
Fig4 M-OCT4

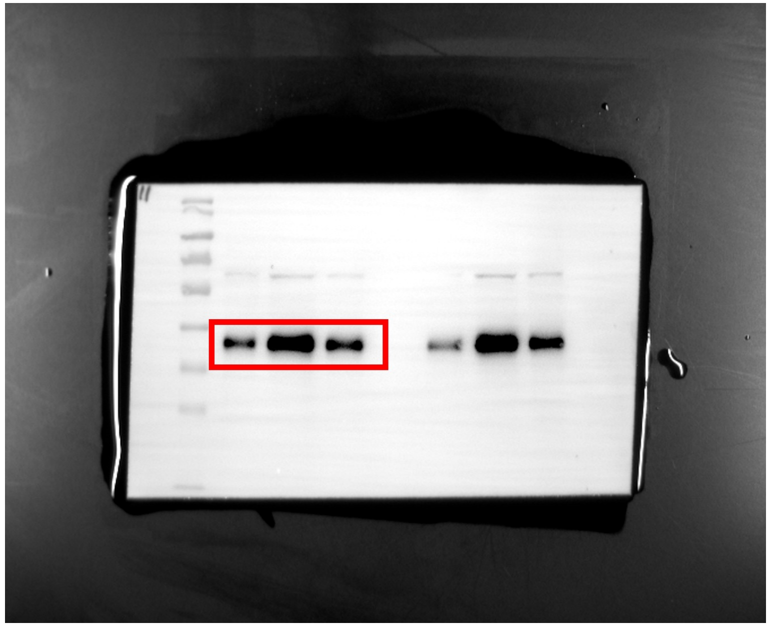
Fig4 M-SOX2

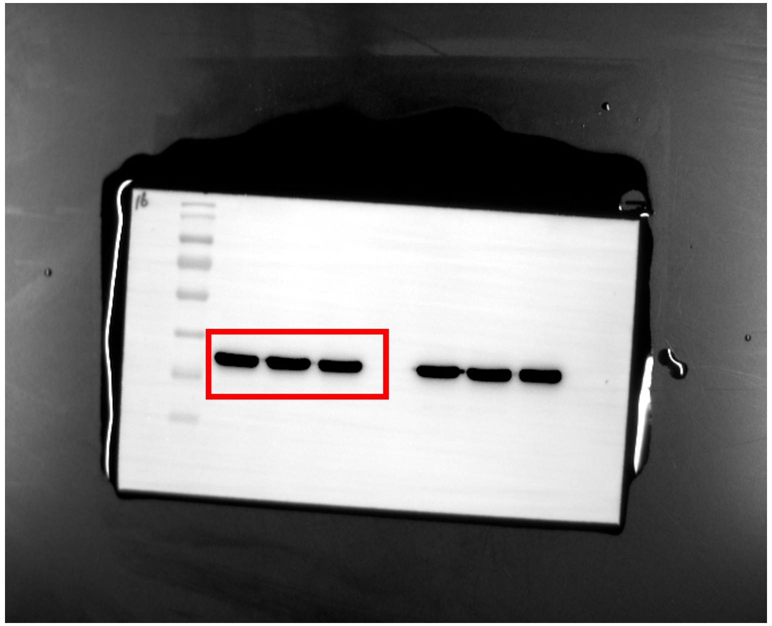
Fig4 M-GAPDH

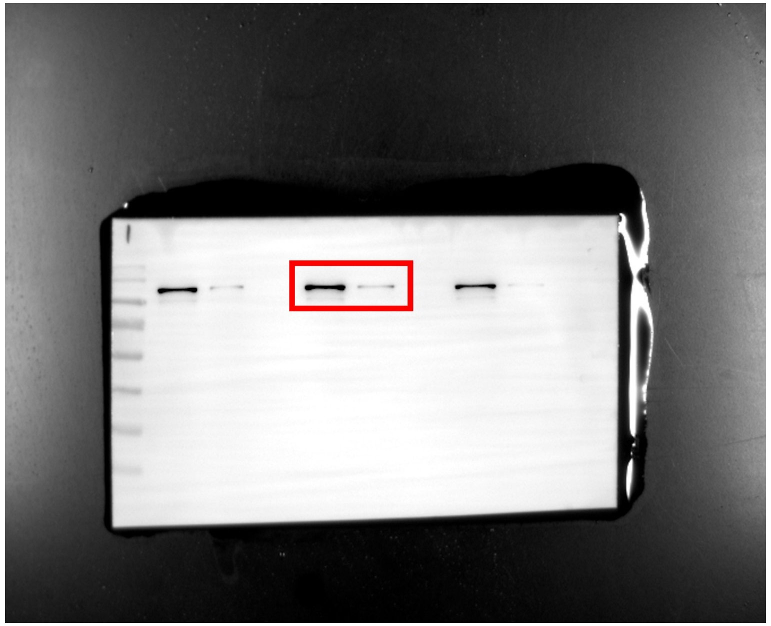

Fig5C-DLG1-1

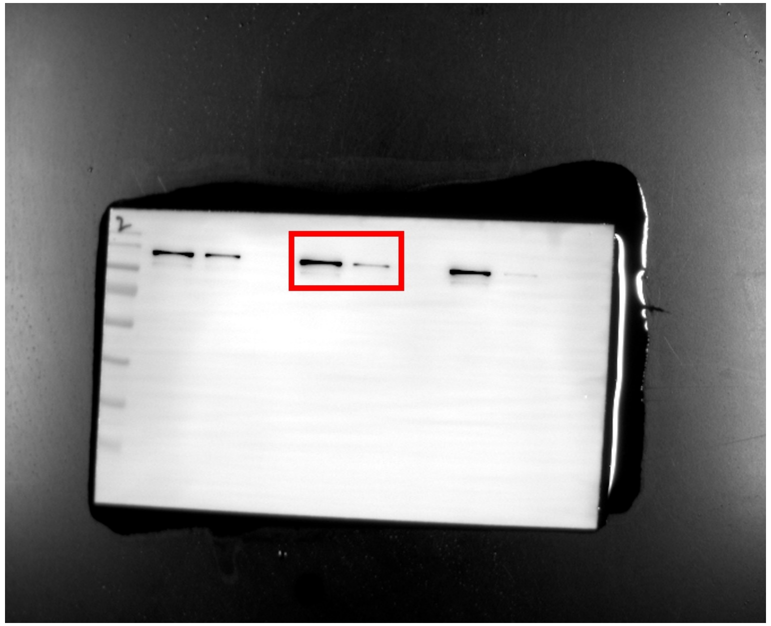

Fig5C-DLG1-2

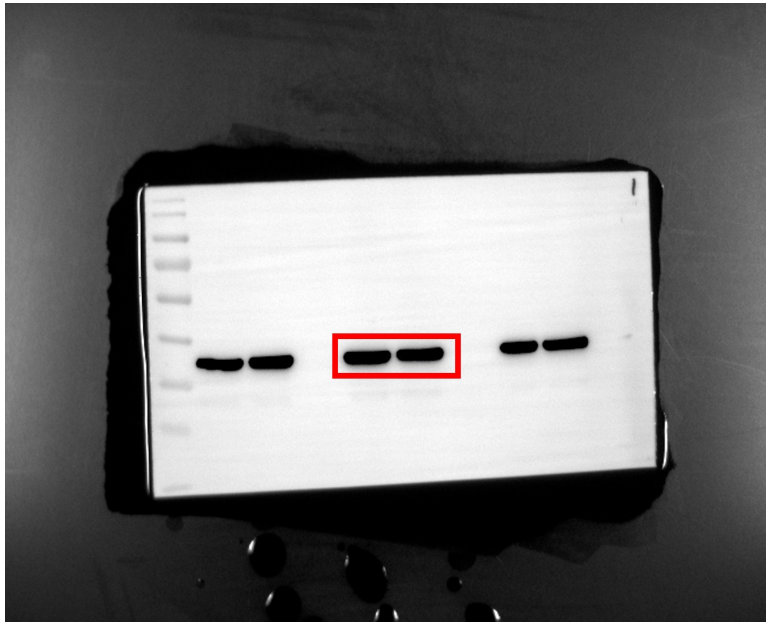

Fig5C-GAPDH-1

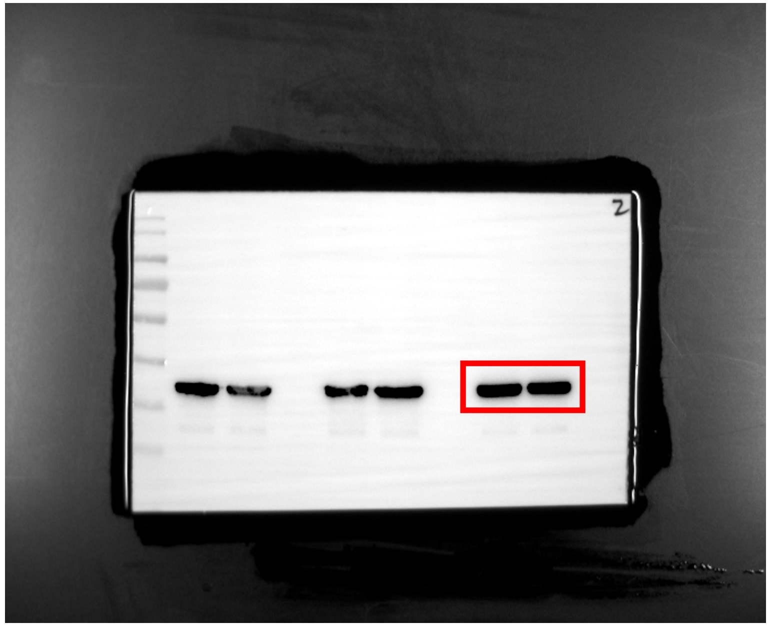

Fig5C-GAPDH-2

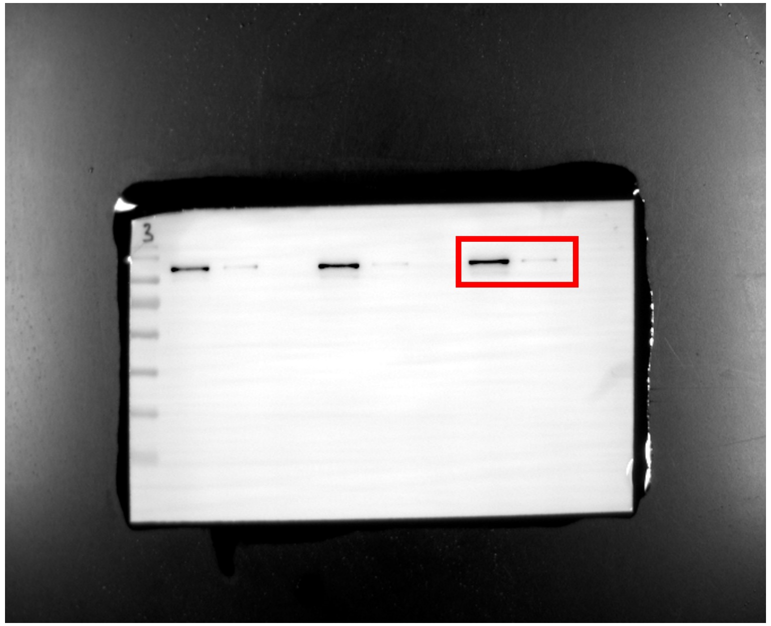

Fig5C-DLG1-3

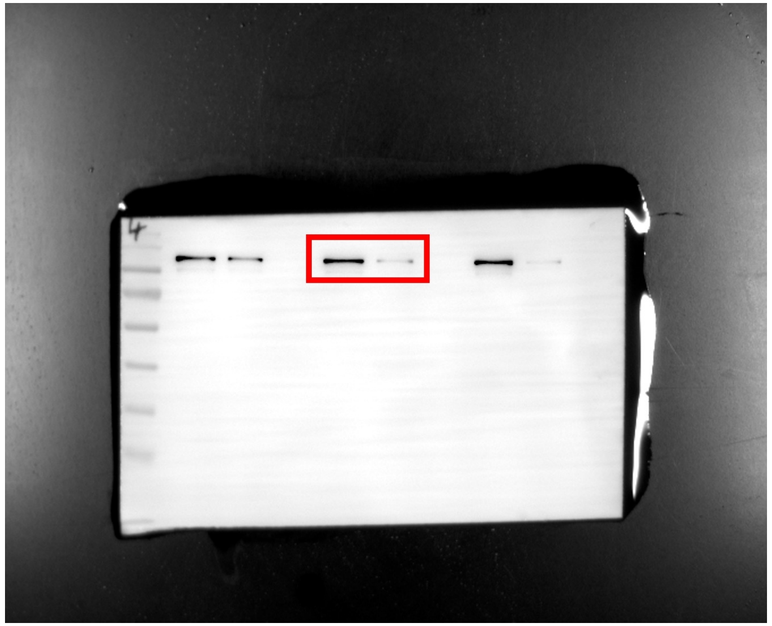

Fig5C-DLG1-4

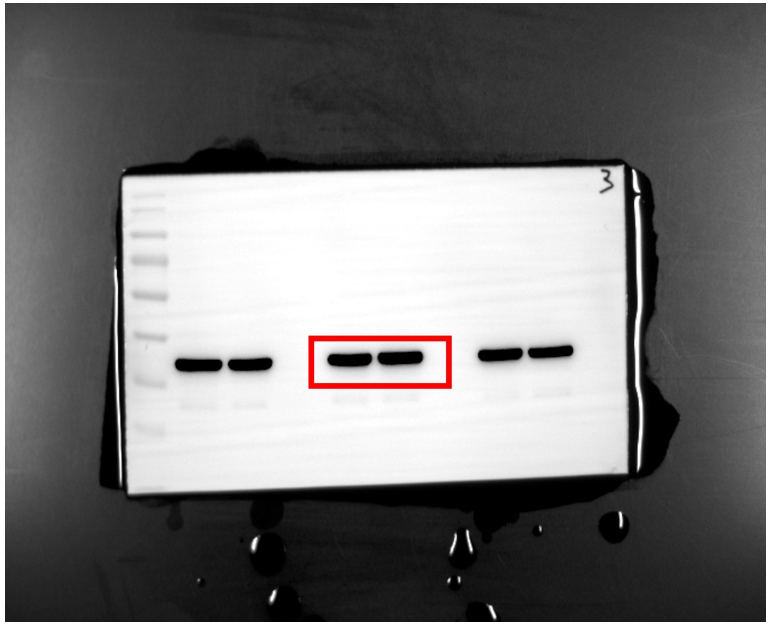

Fig5C-GAPDH-3

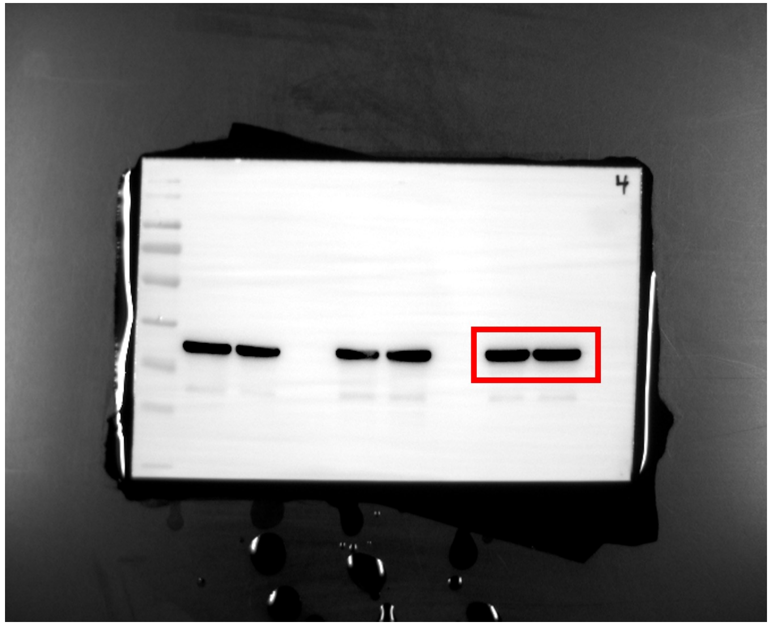

Fig5C-GAPDH-4

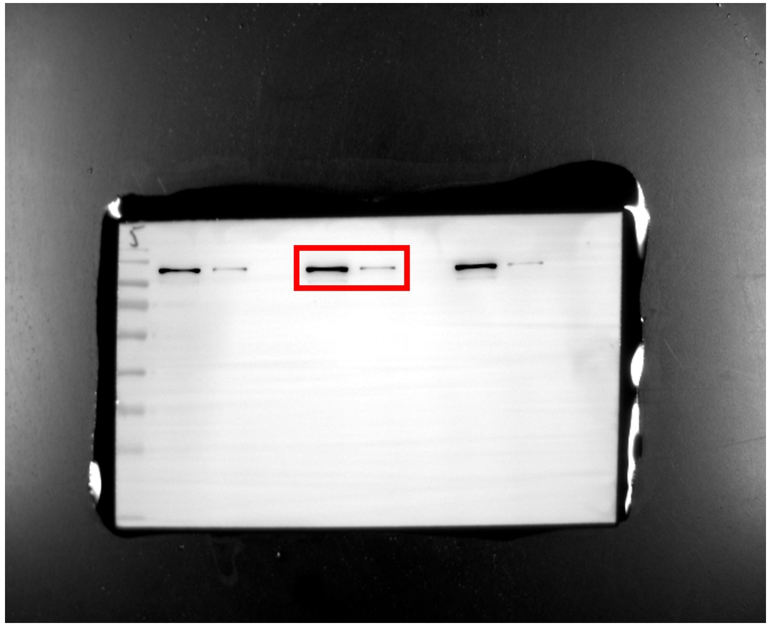

Fig5C-DLG1-5

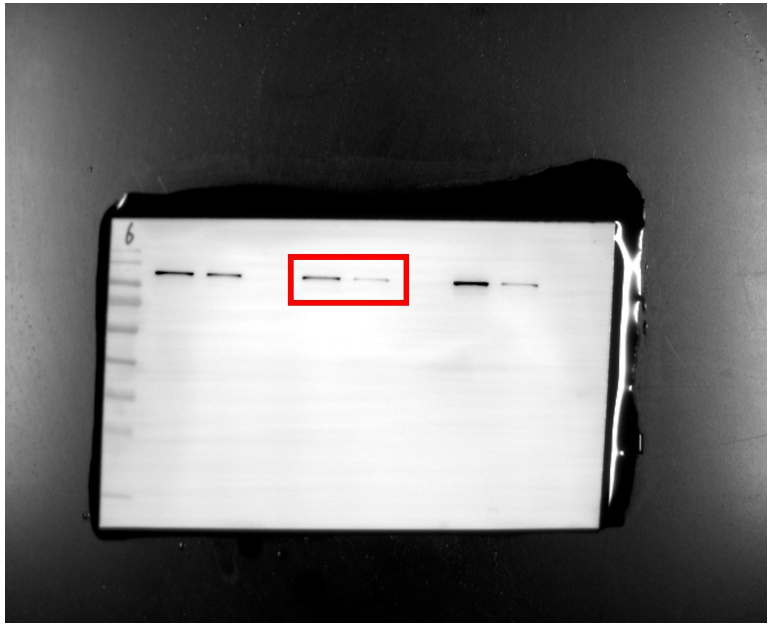

Fig5C-DLG1-6

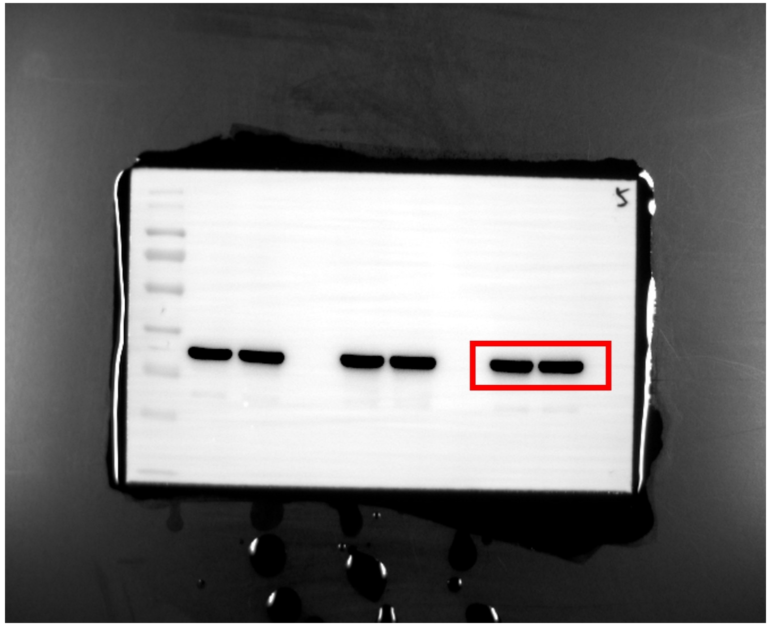

Fig5C-GAPDH-5

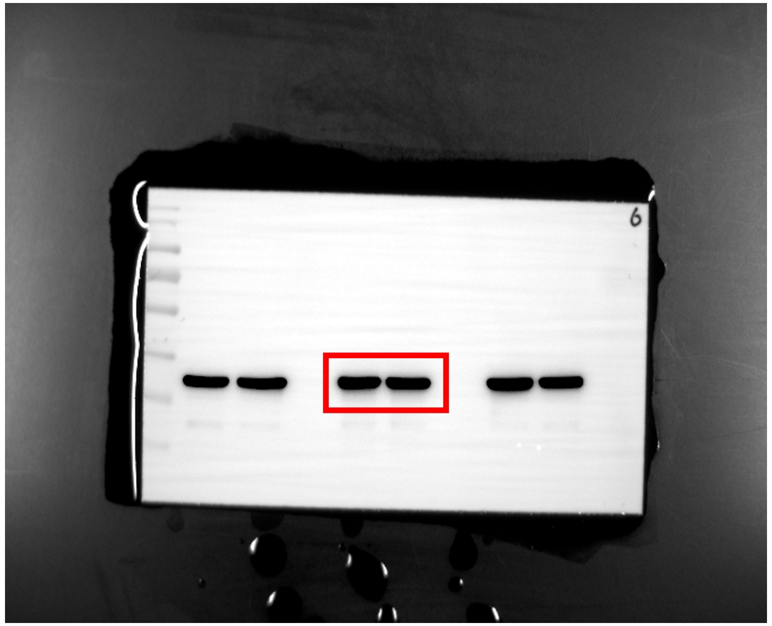

Fig5C-GAPDH-6

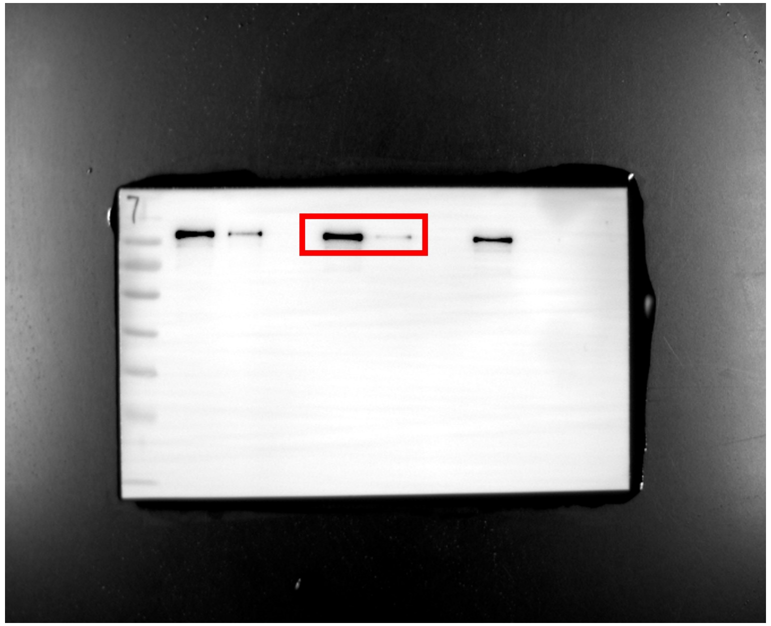

Fig5C-DLG1-7

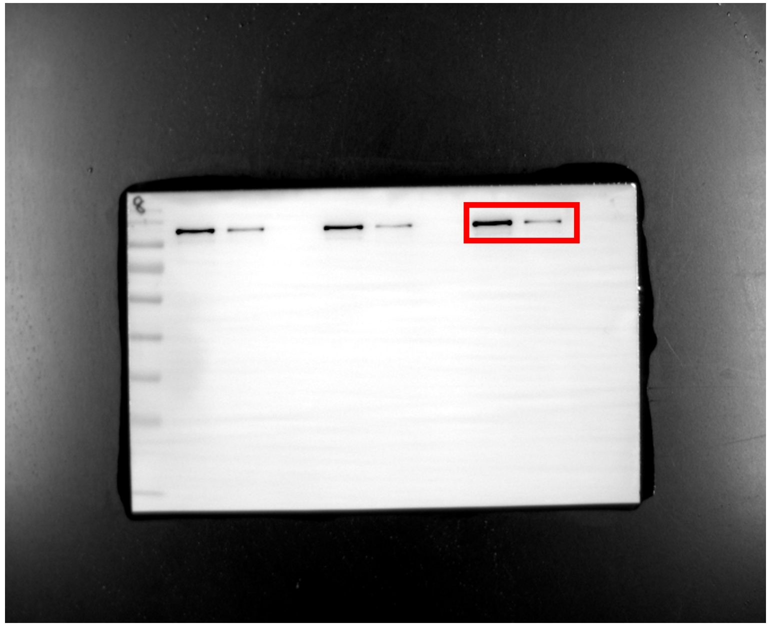

Fig5C-DLG1-8

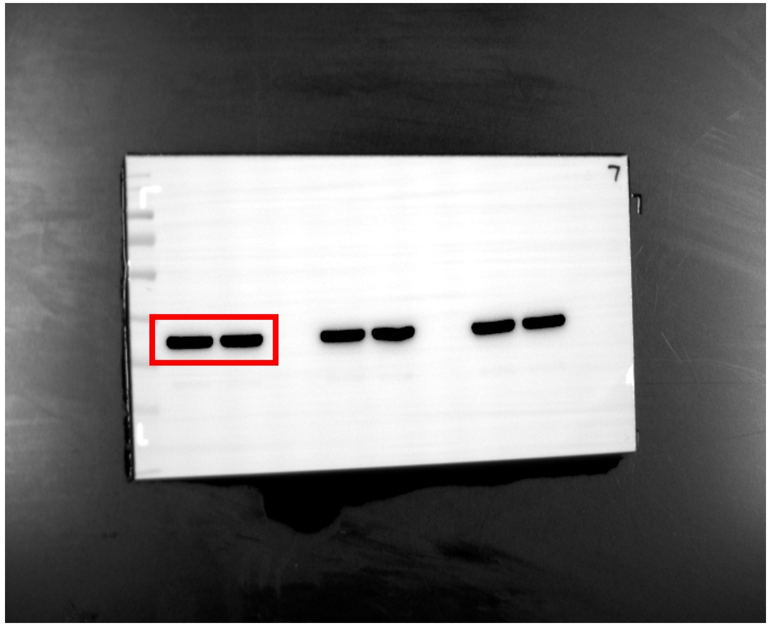

Fig5C-GAPDH-7

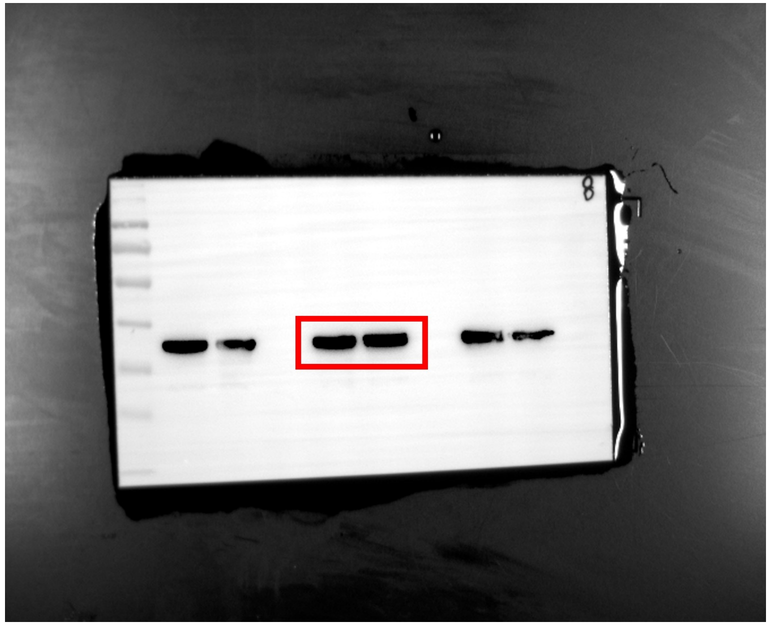

Fig5C-GAPDH-8

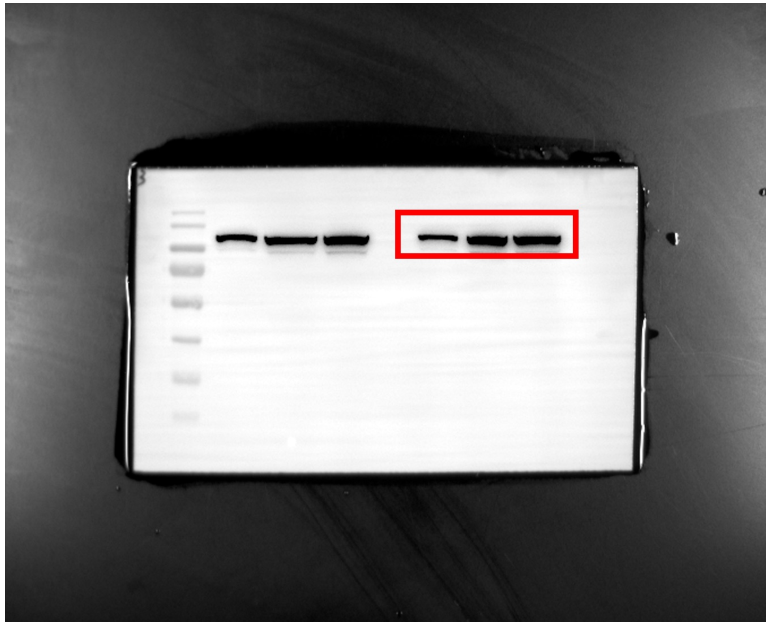

F5I-DLG1-BxPC-3

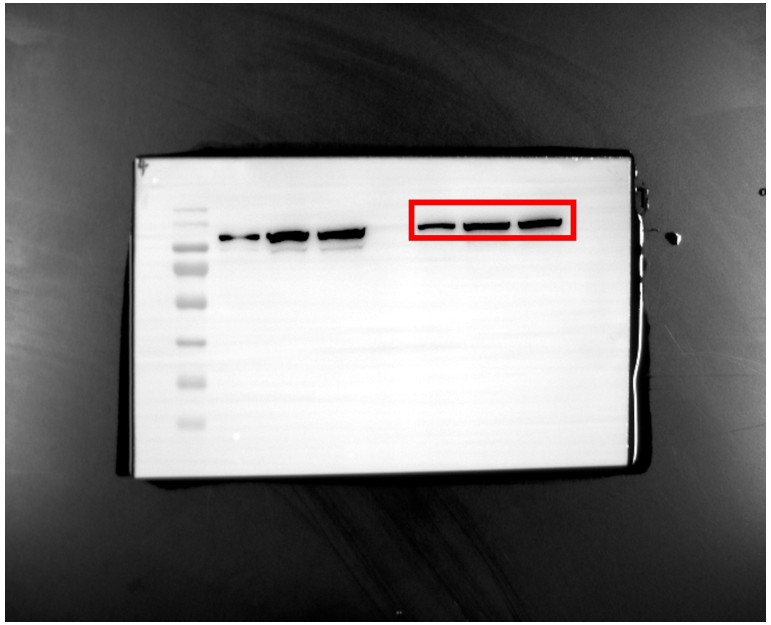

F5I-DLG1-CFPAC-1

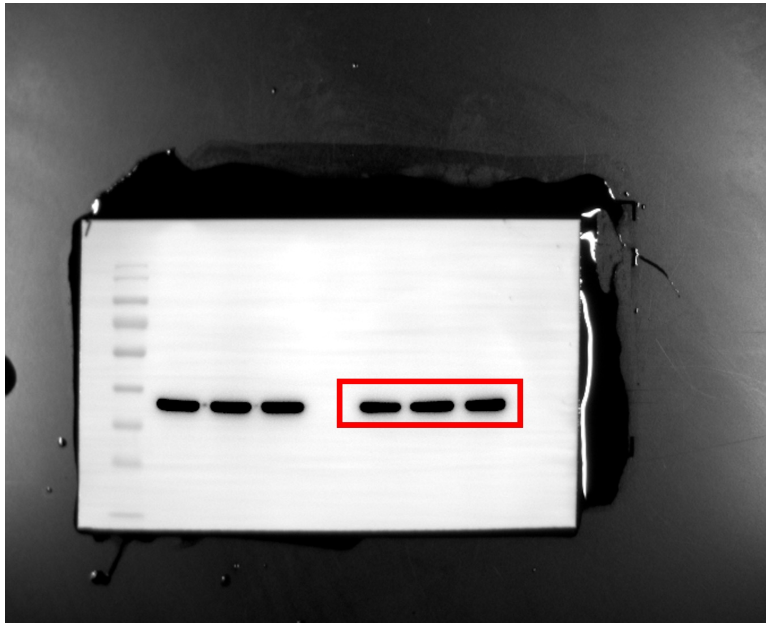

F5I-GAPDH-BxPC-3

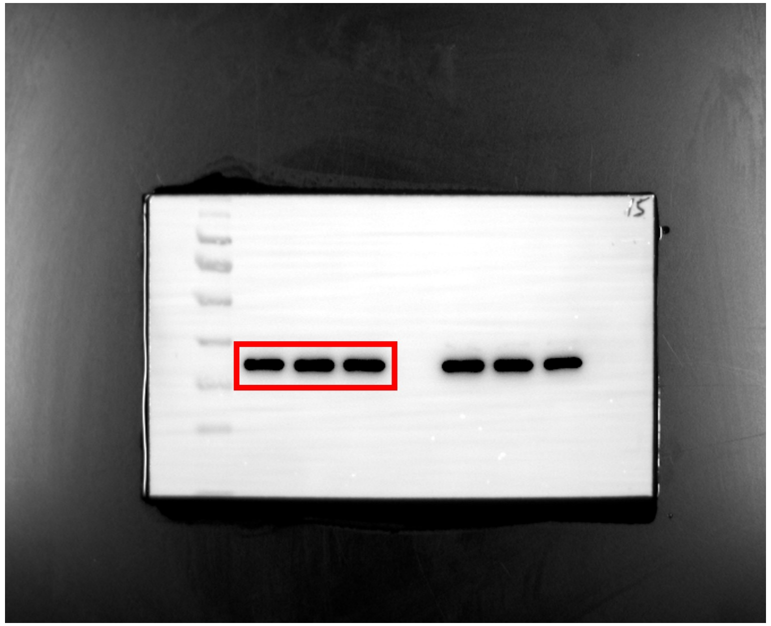

F5I-GAPDH-CFPAC-1

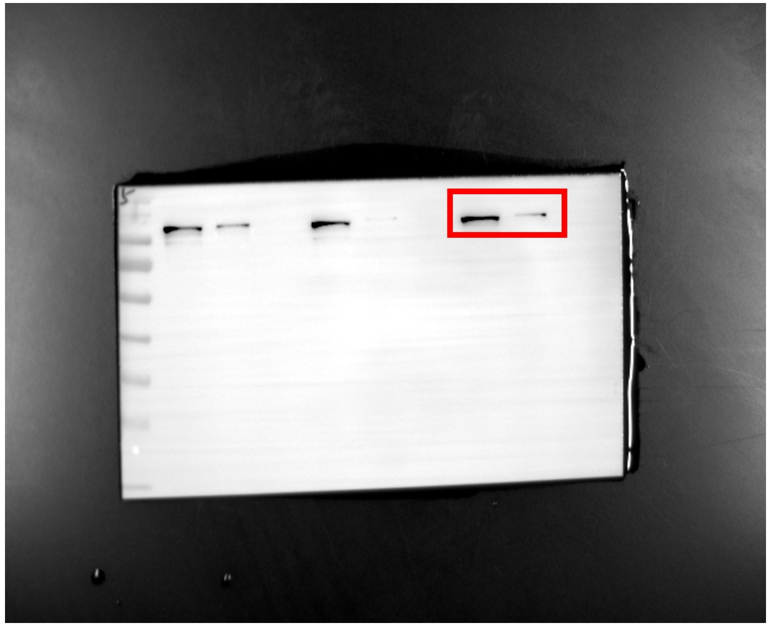

F5I-DLG1-Capan-1

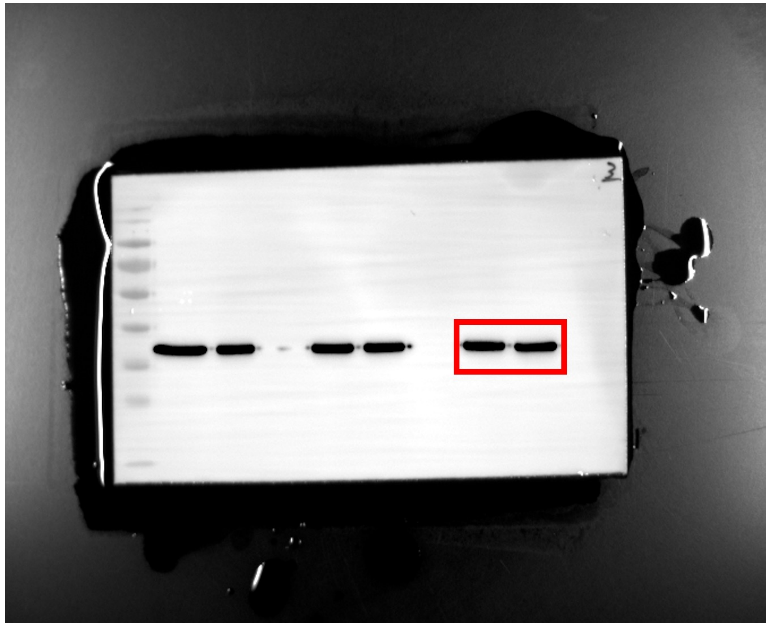

F5I-GAPDH-Capan-1

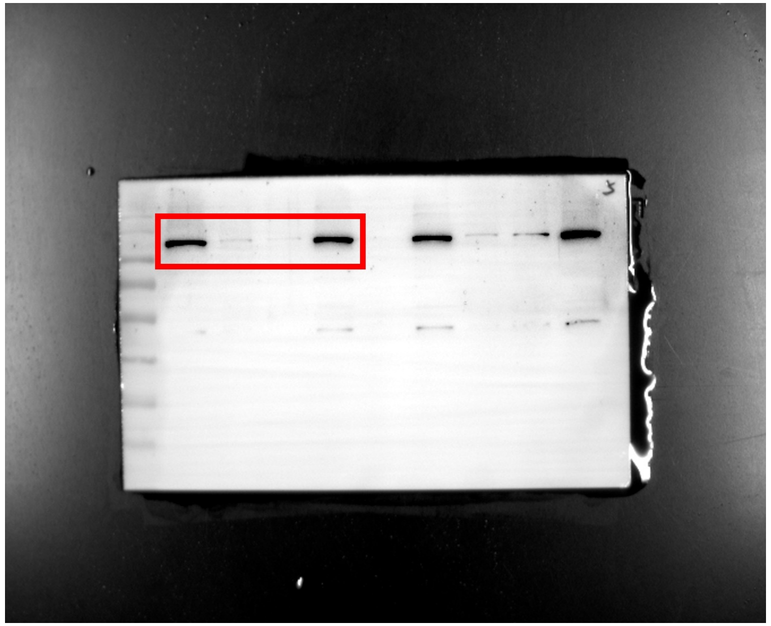

F5O-CD133

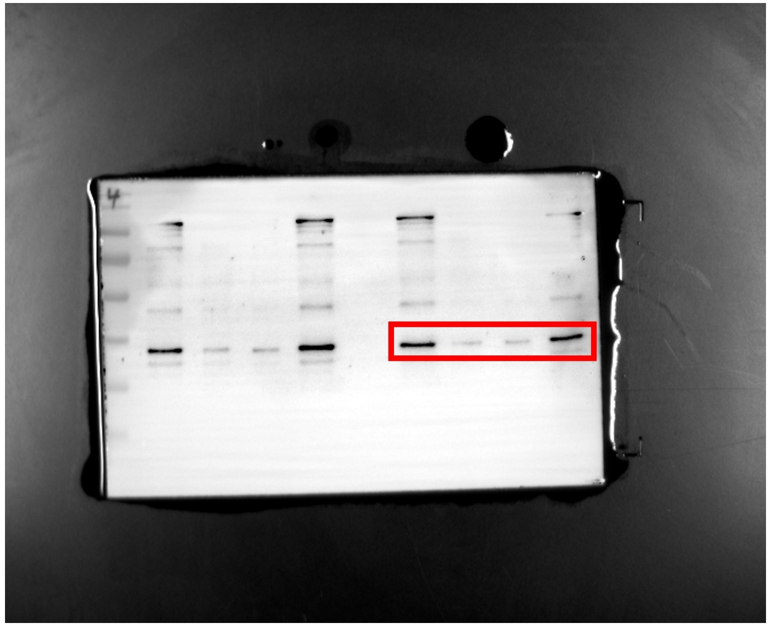

F5O-EpCAM

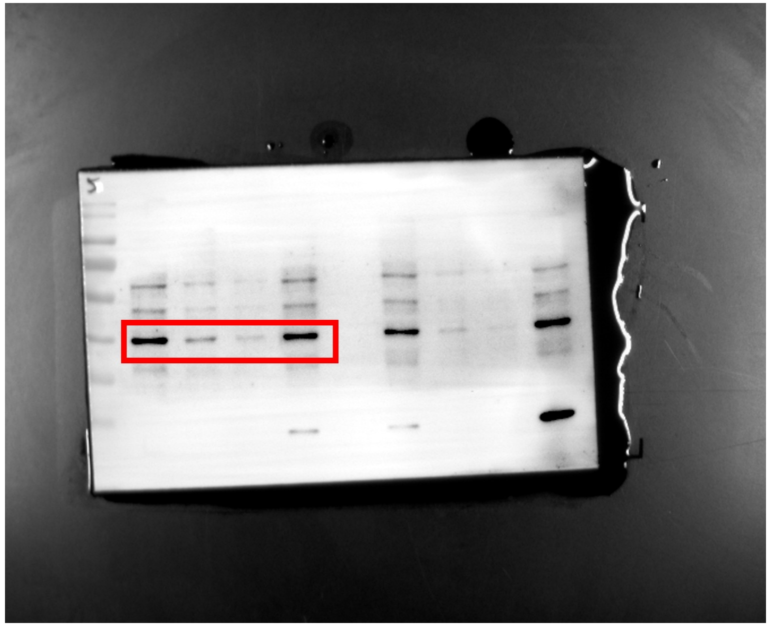

F5O-Nanog

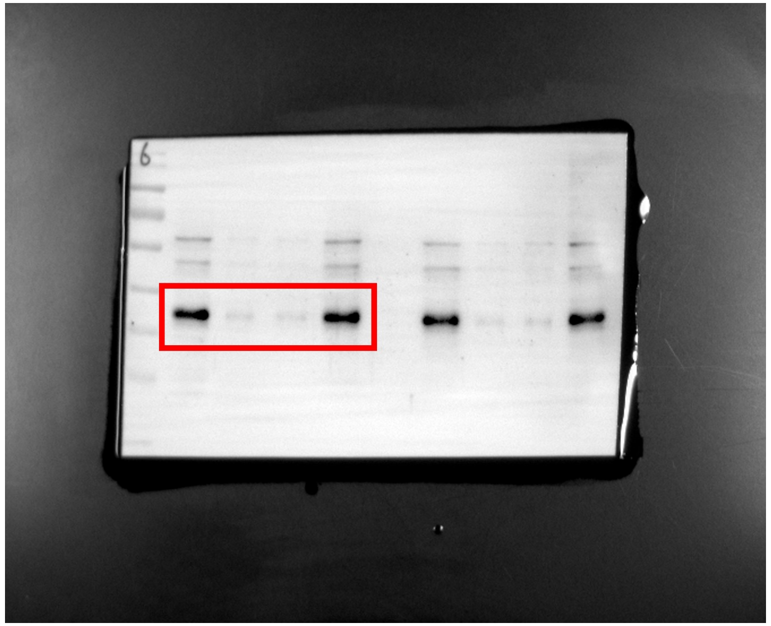

F5O-SOX2

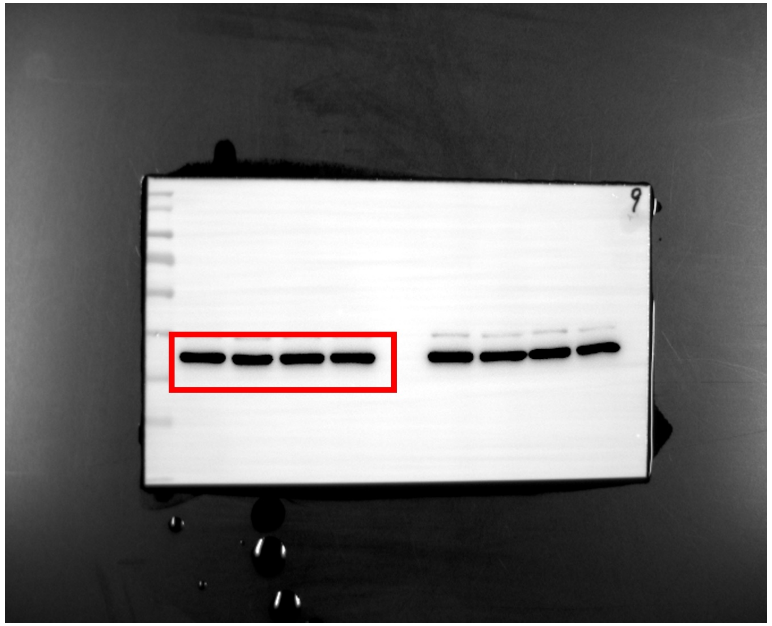

F5O-GAPDH

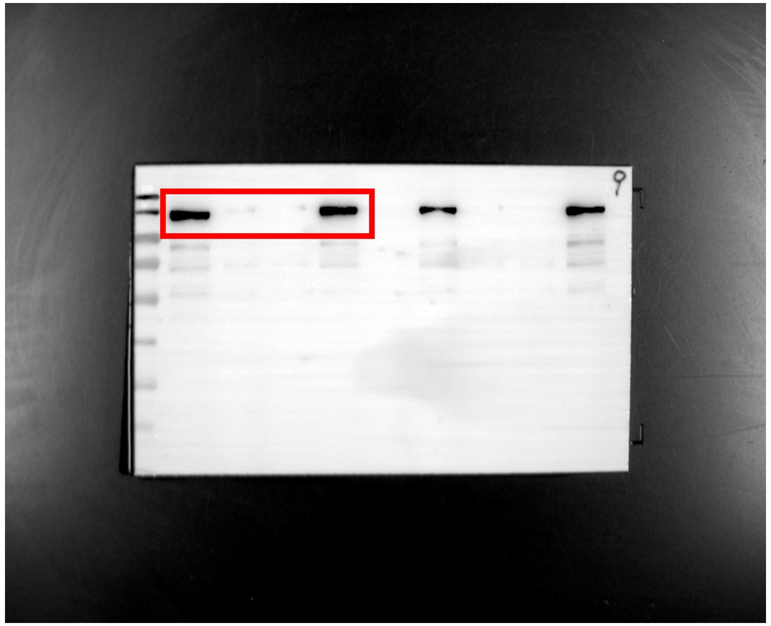
F5P-SREBP1

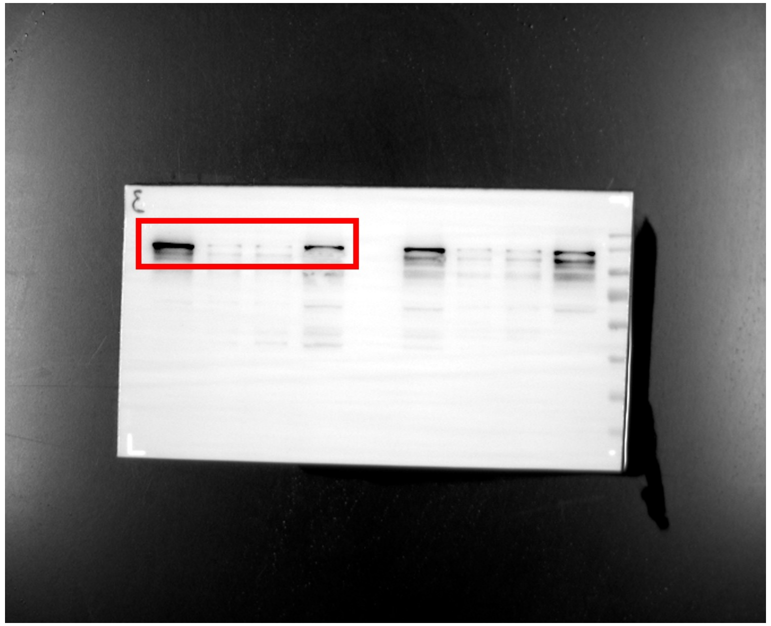
F5P-ACLY

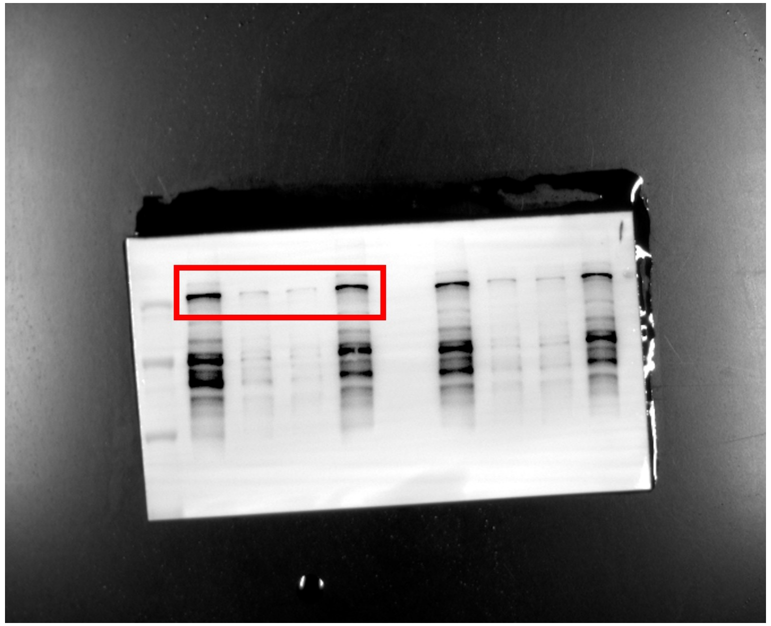
F5P-FASN

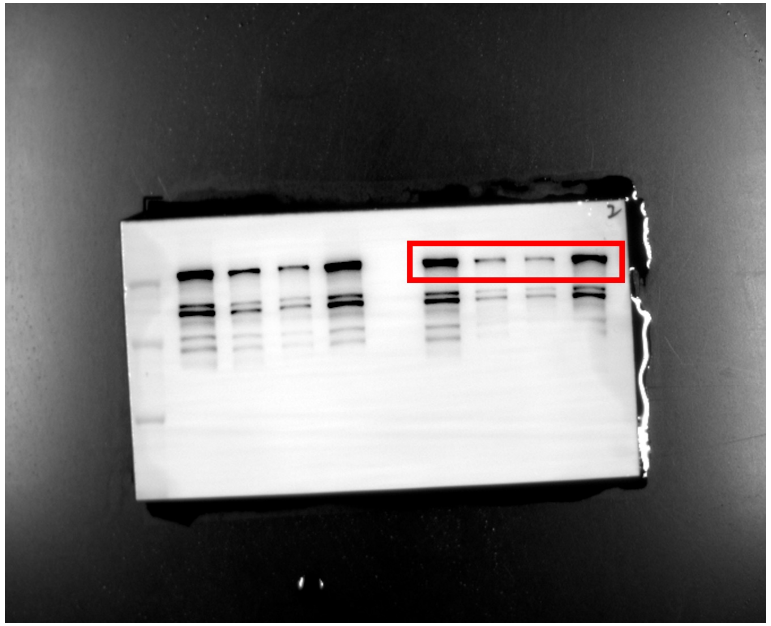
F5P-ACC

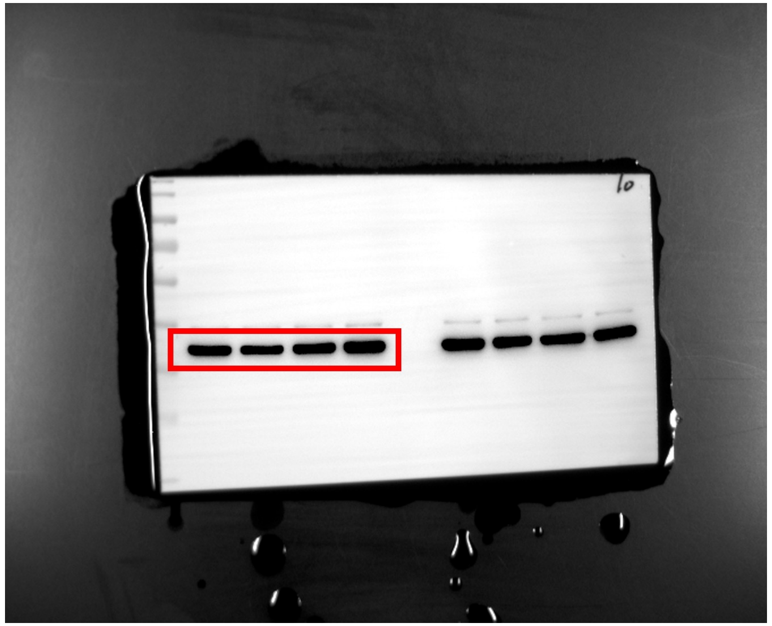
F5P-GAPDH

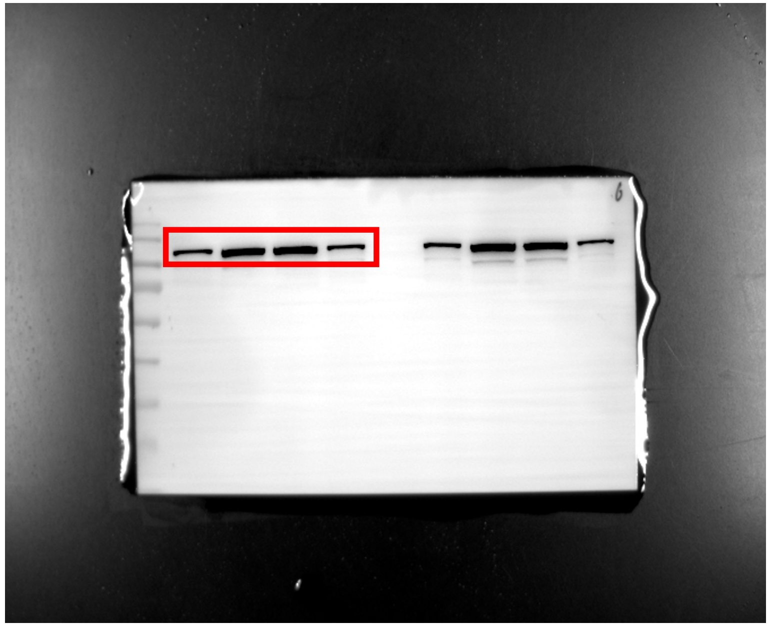

F5Q-DLG1

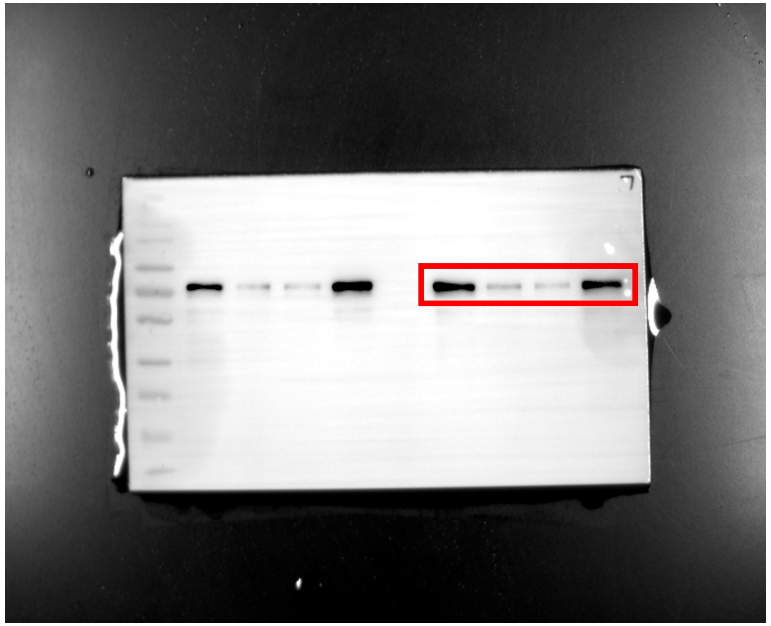

F5Q-Nuc-YAP

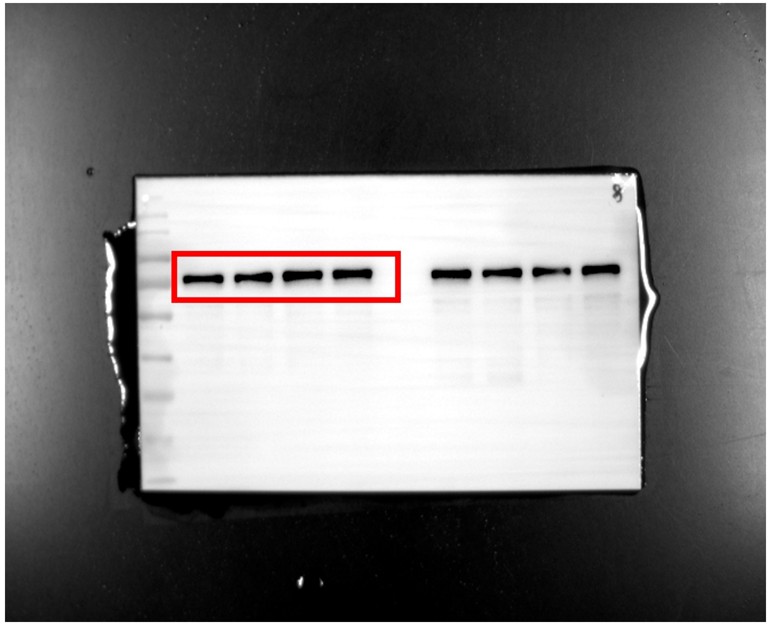

F5Q-Cyt-YAP

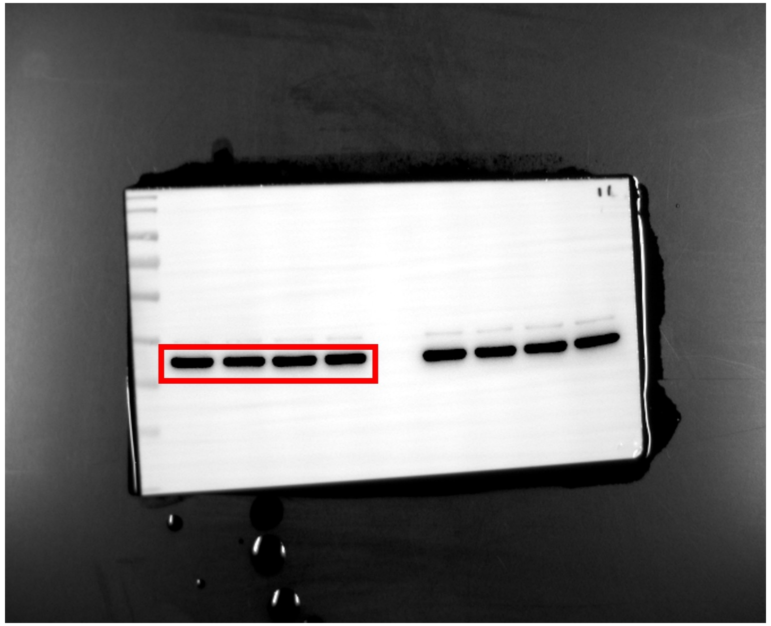

F5Q-GAPDH

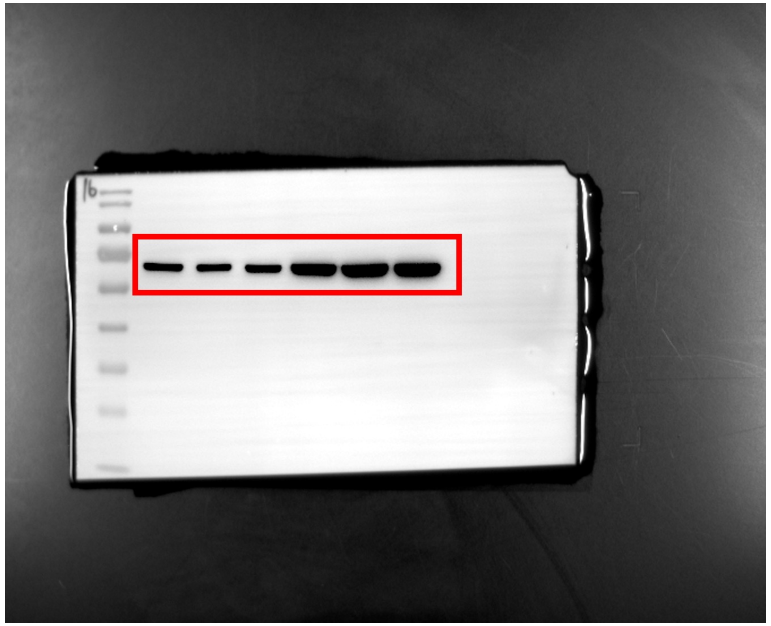

F7A-FTO-P2

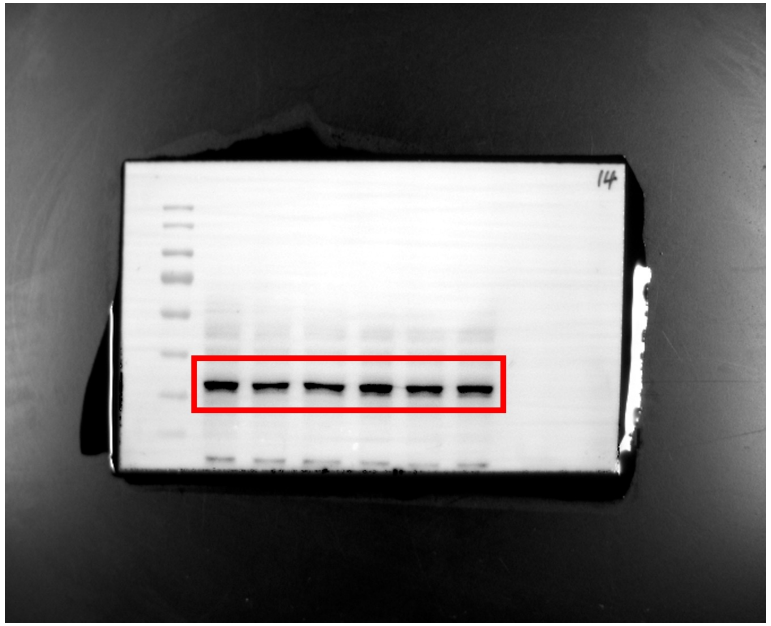

F7A-ALKBH5-P2

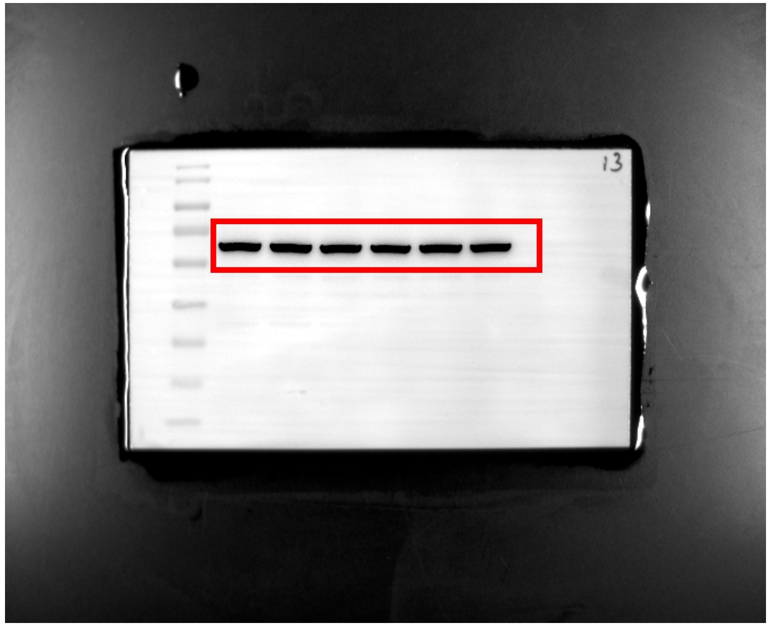

F7A-METTL3-P2

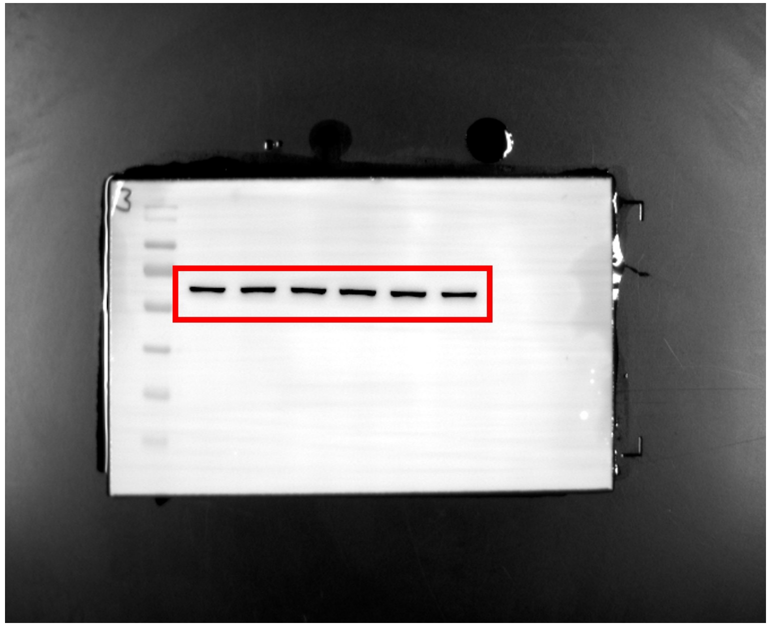

F7A-METTL14-P2

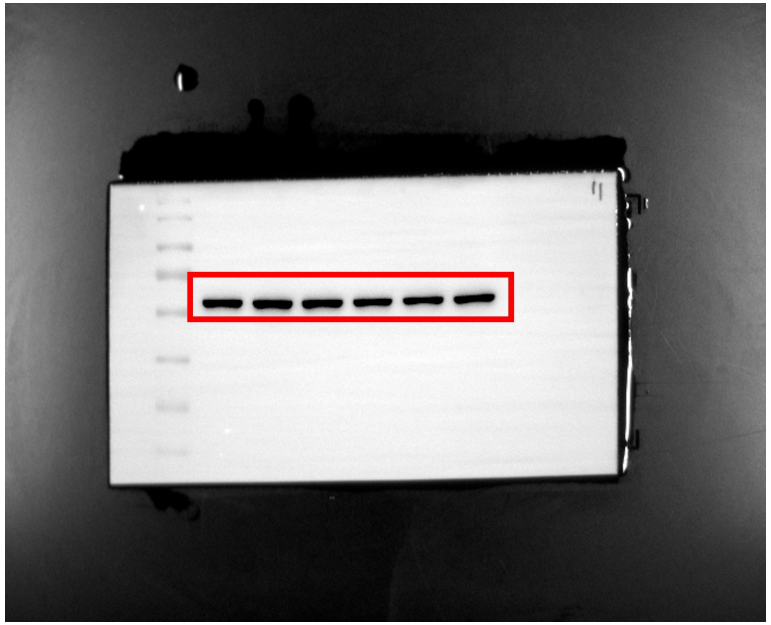

F7A-WTAP-P2

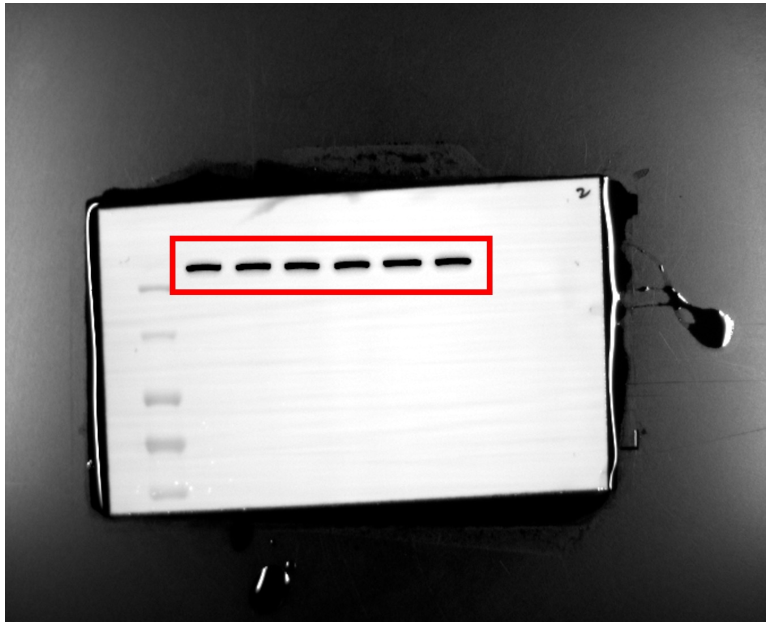

F7A-VIPMA-P2

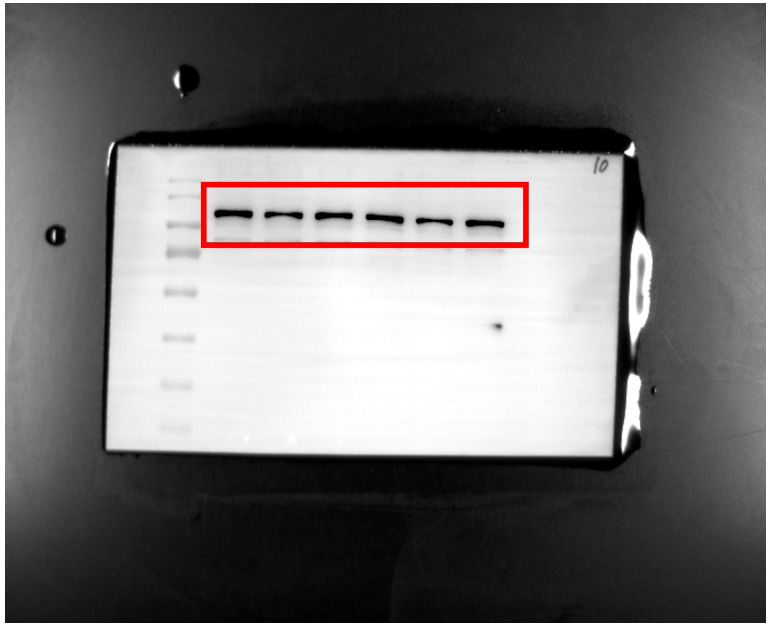

F7A-RBM15-P2

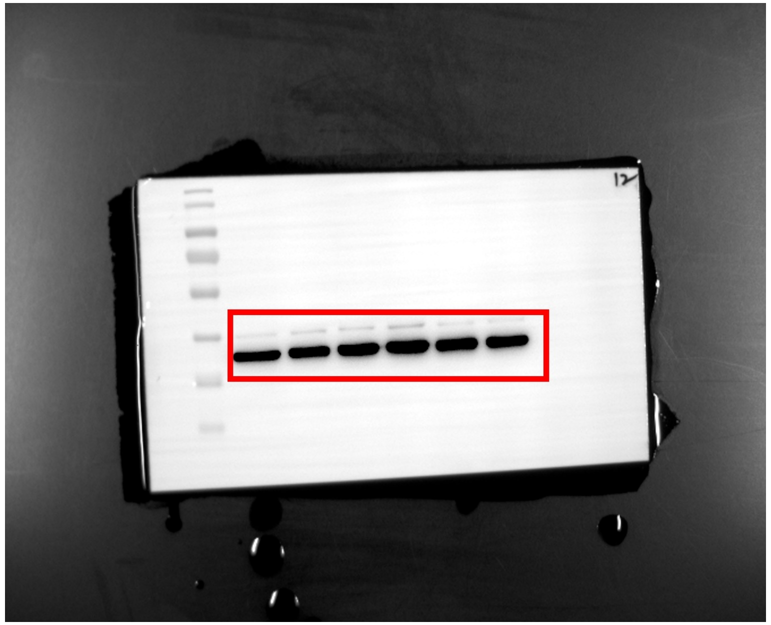

F7A-GAPDH-P2

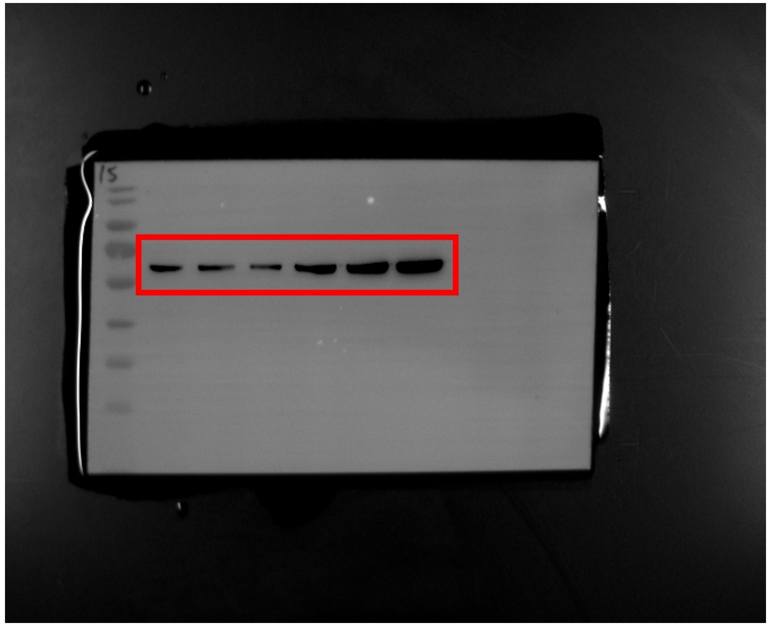

F7A-FTO-P3

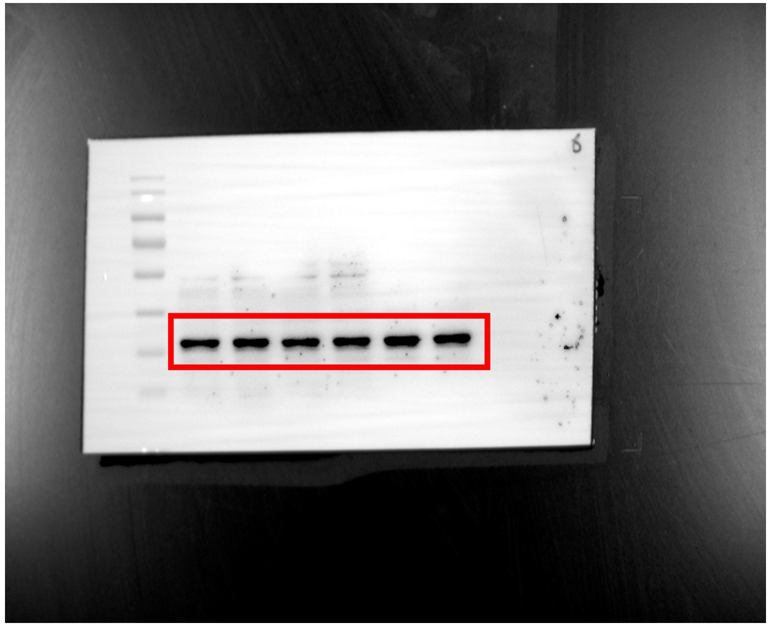

F7A-ALKBH5-P3

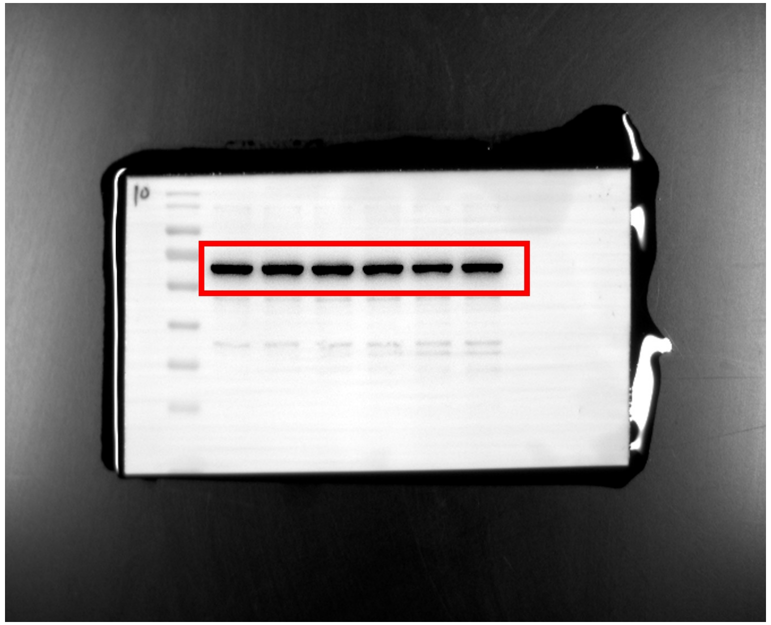

F7A-METTL3-P3

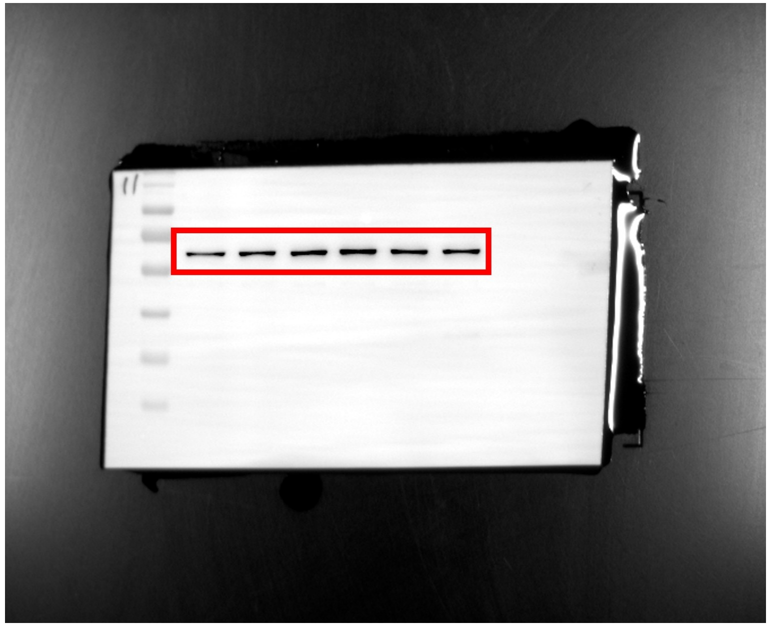

F7A-METTL14-P3

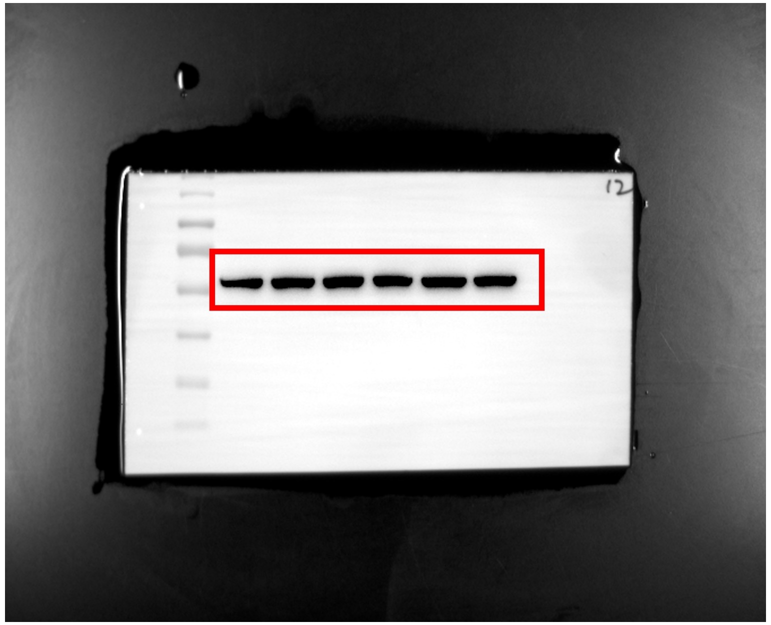

F7A-WTAP-P3

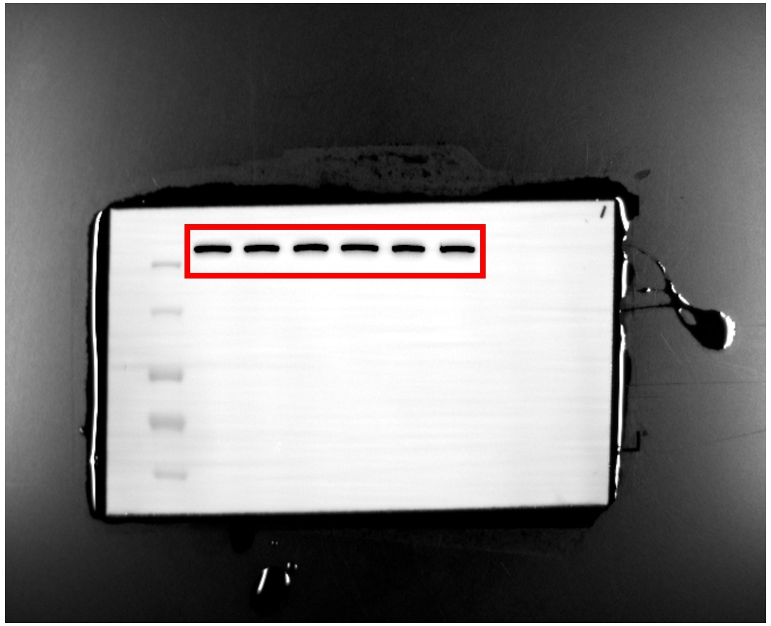

F7A-VIPMA-P3

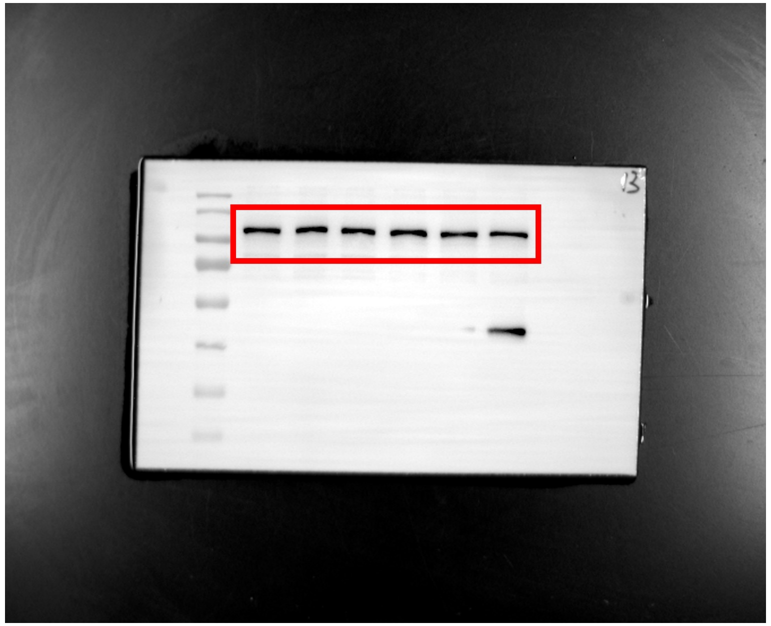

F7A-RBM15-P3

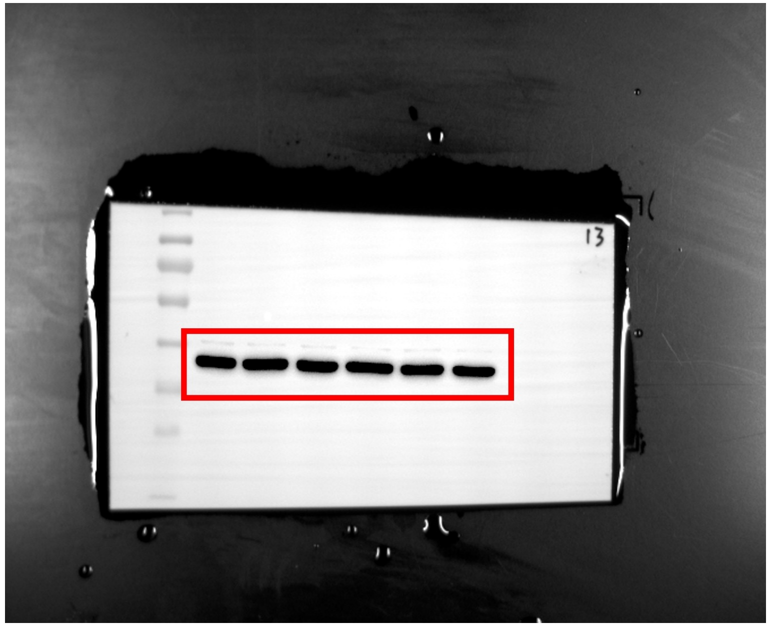

F7A-GAPDH-P3

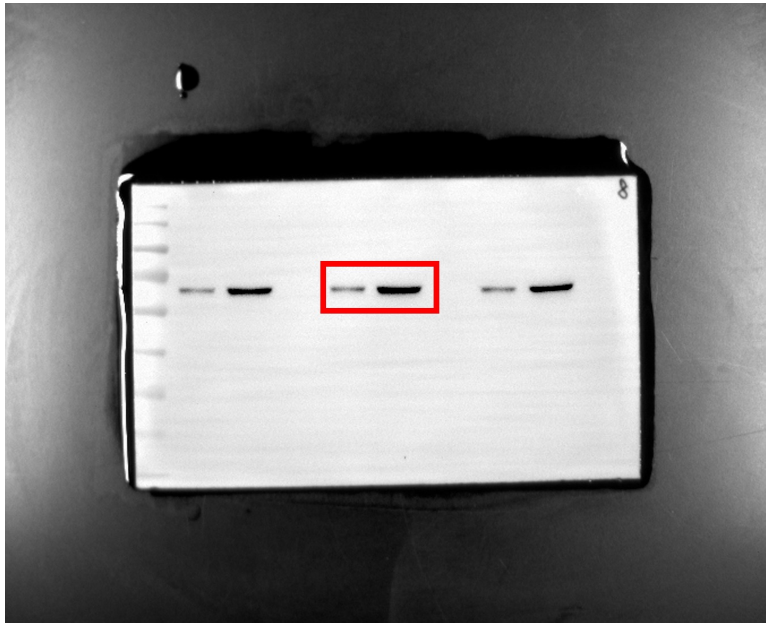

F7G-FTO

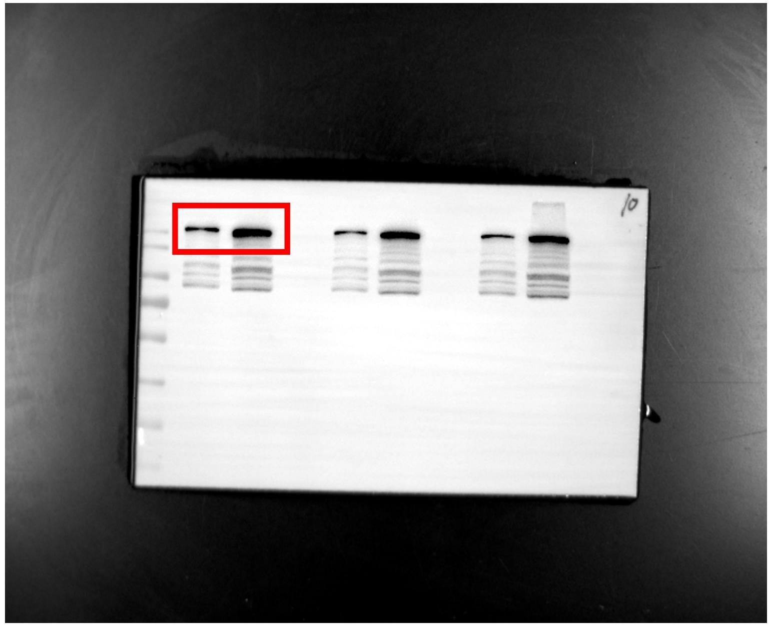

F7G-KDM5B

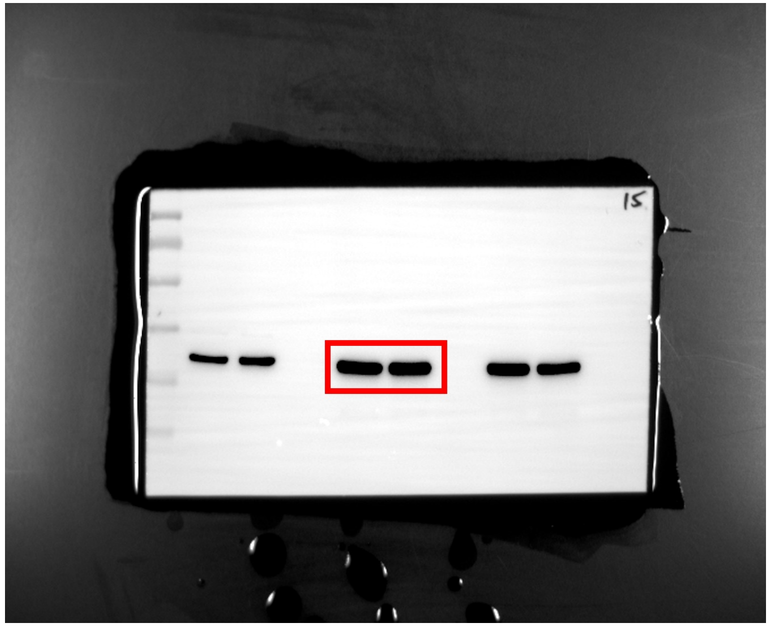

F7G-GAPDH

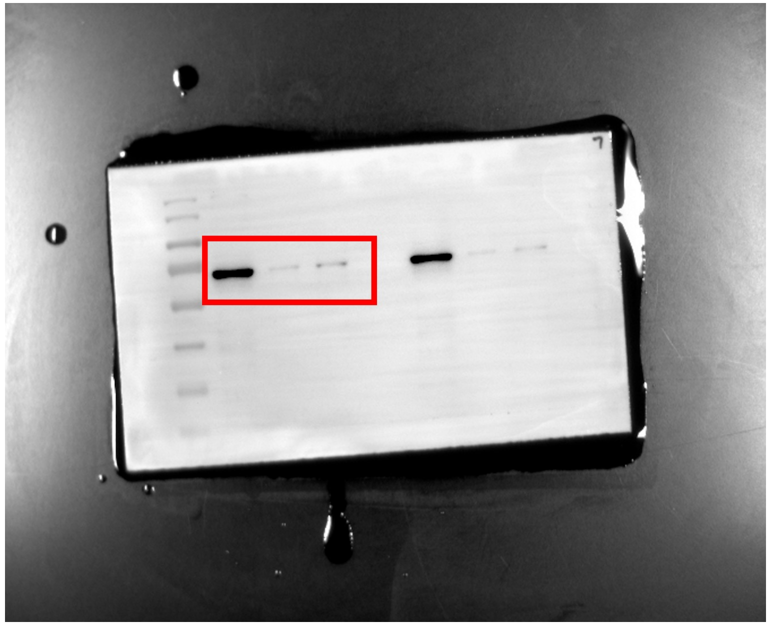

F7H-FTO

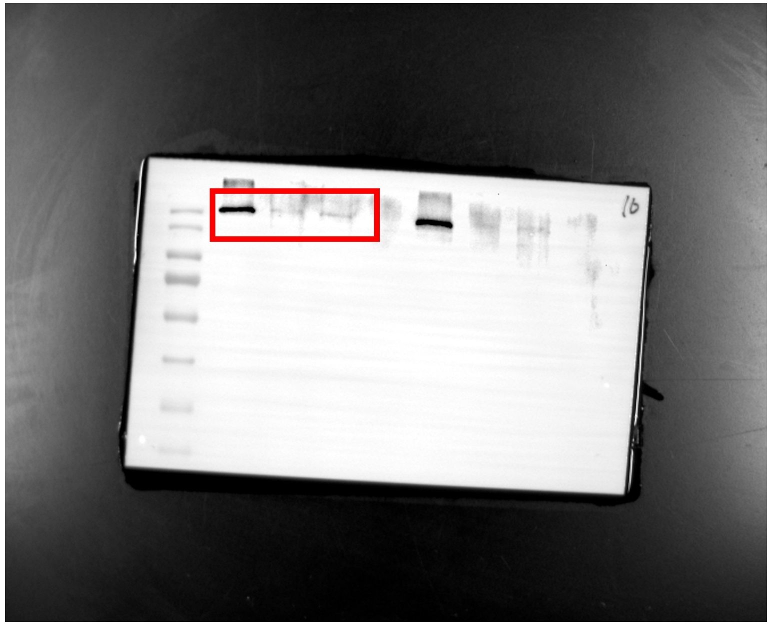

F7H-KDM5B

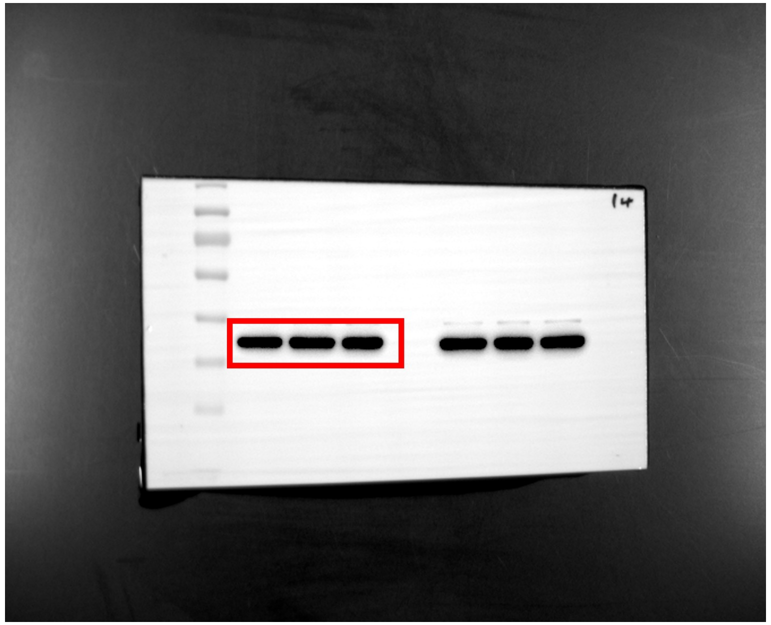

F7H-GAPDH

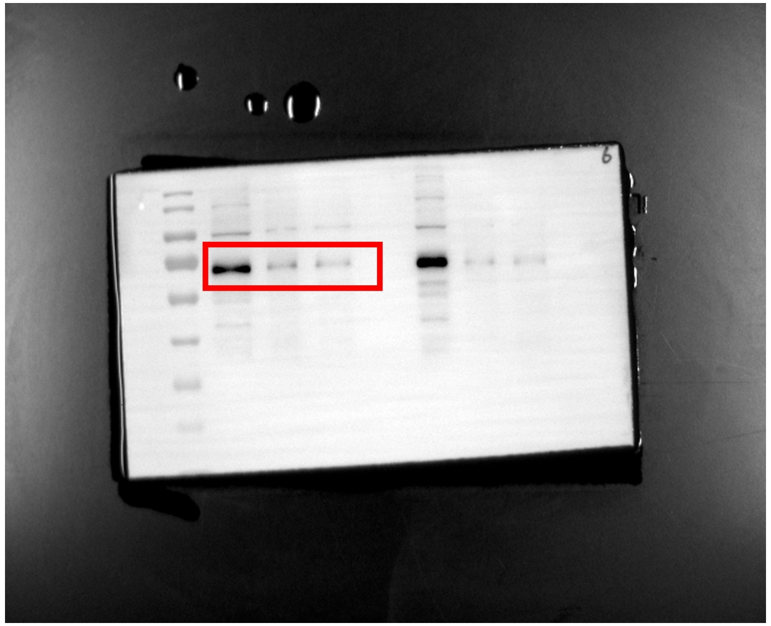

F7L-YTHDF2

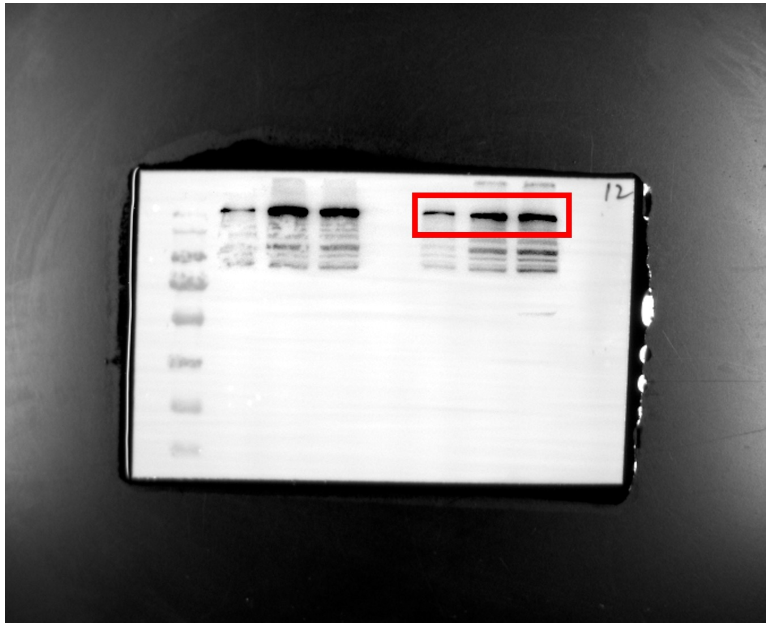

F7L-KDM5B

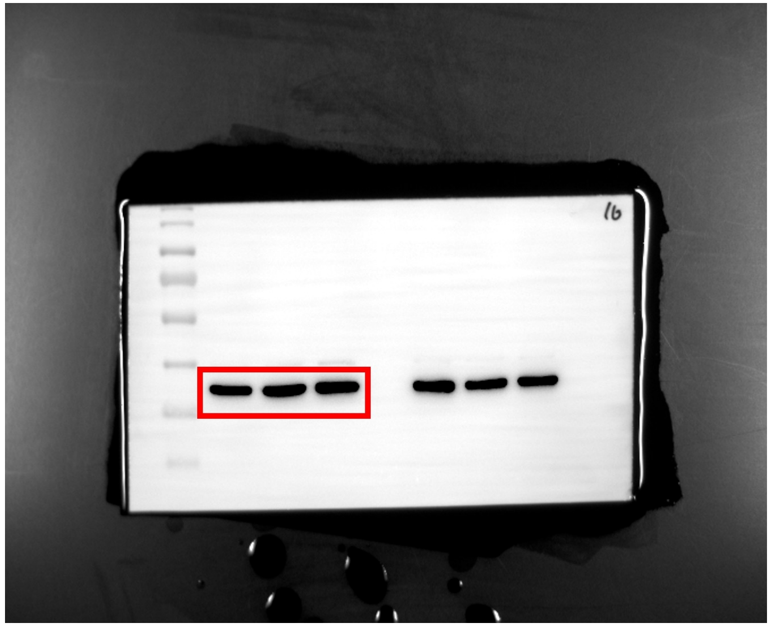

F7L-GAPDH

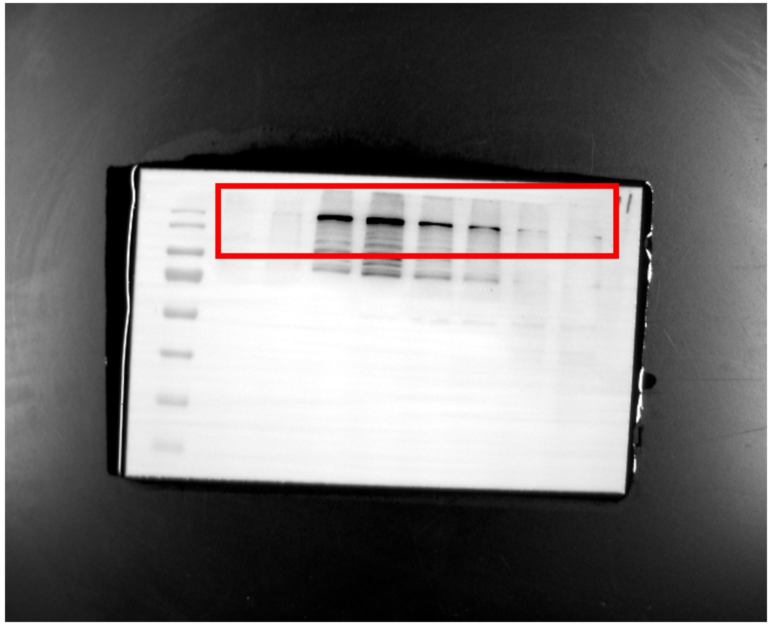

S2A-KDM5B

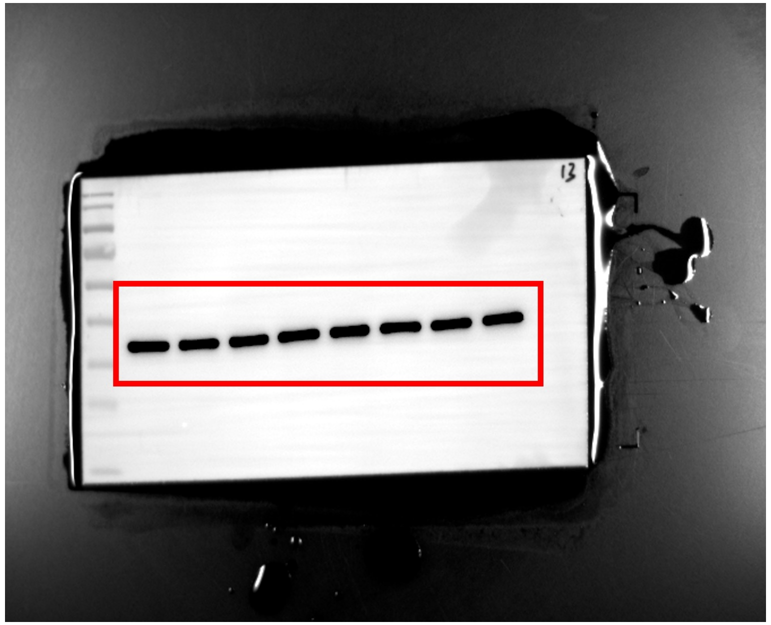

S2A-GAPDH

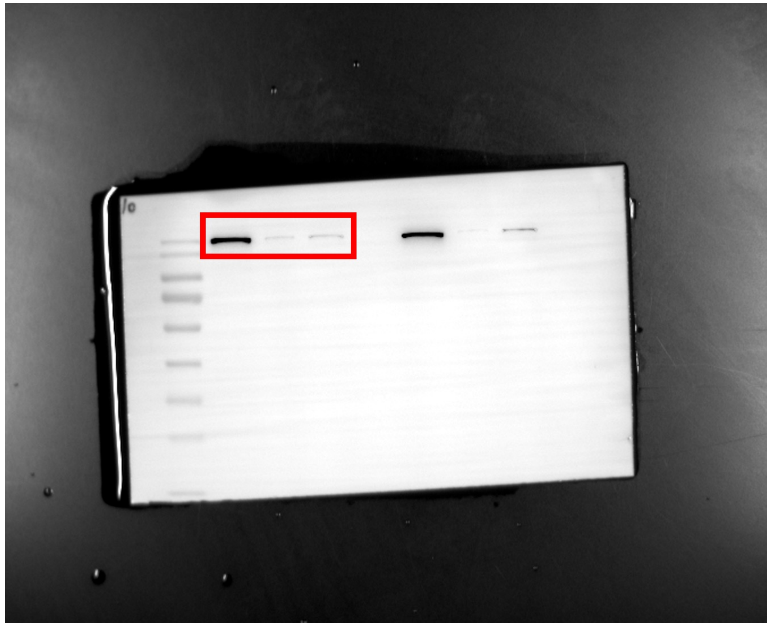

S2B-KDM5B-BxPC-3

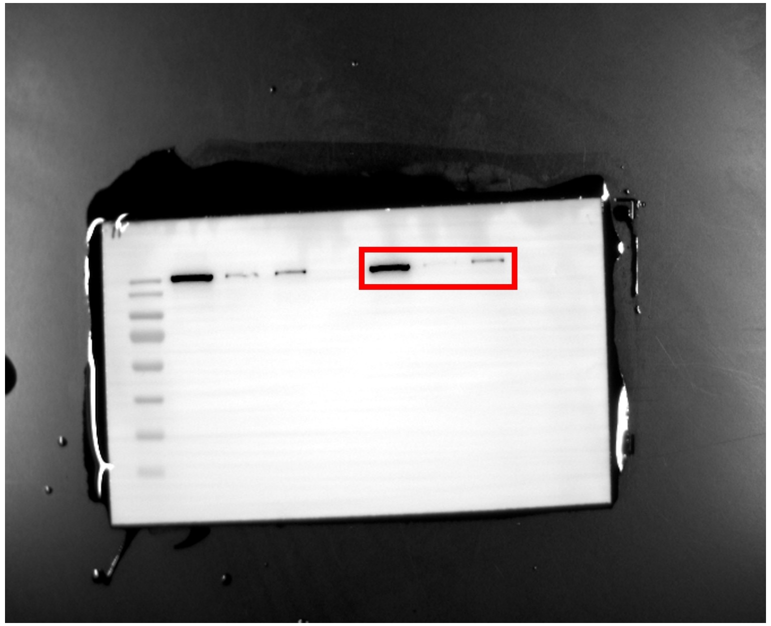

S2B-KDM5B-CFPAC-1

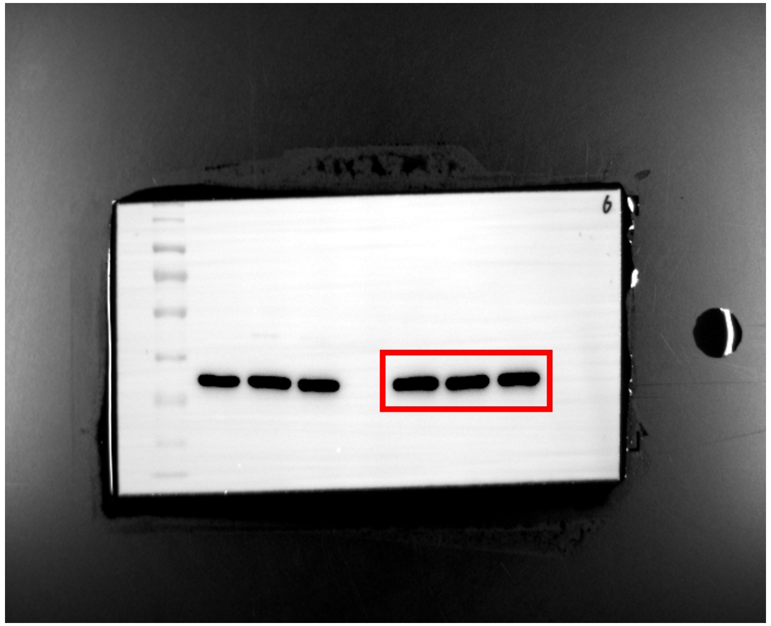

S2B-GAPDH-BxPC-3

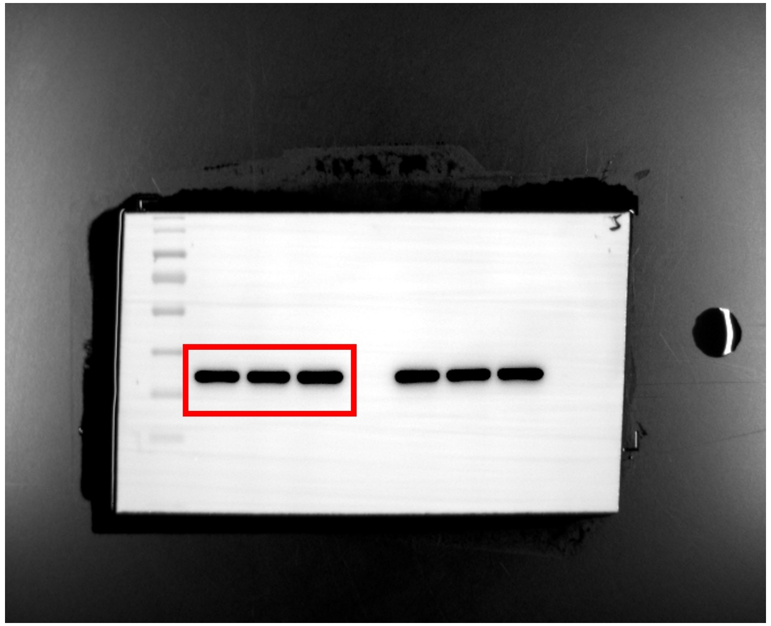

S2B-GAPDH-CFPAC-1

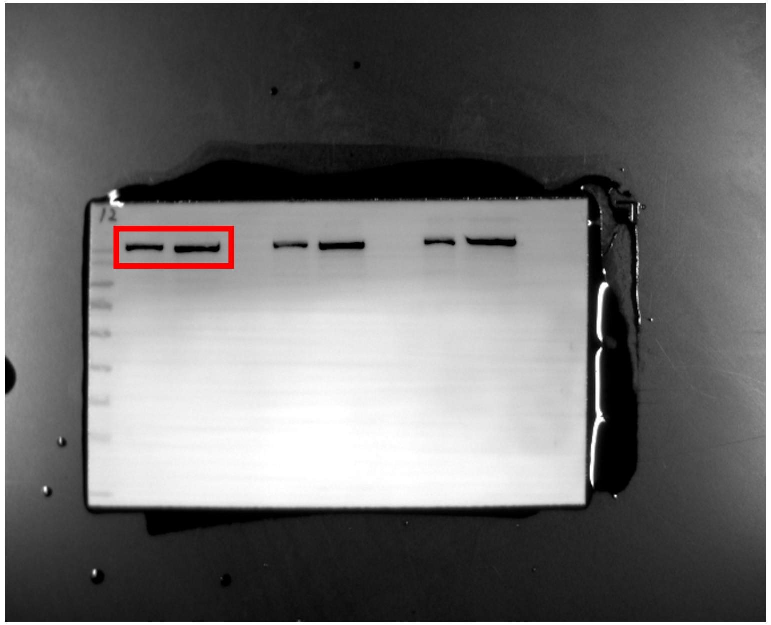

S2D-KDM5B-Capan-1

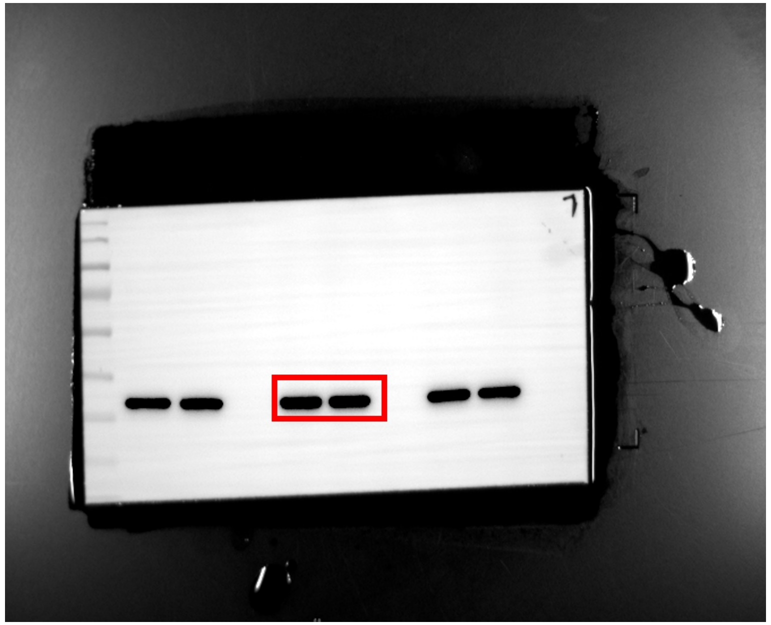

S2D-GAPDH-Capan-1

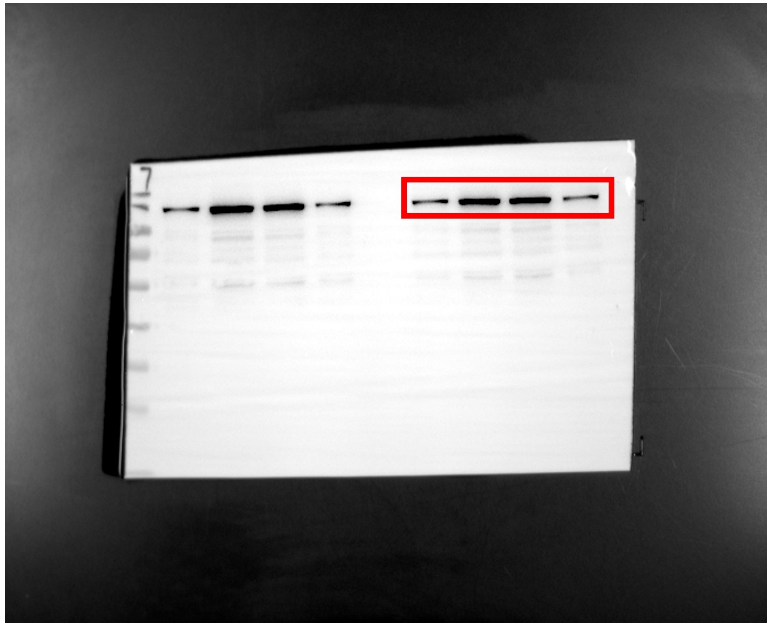
S5I-SREBP1

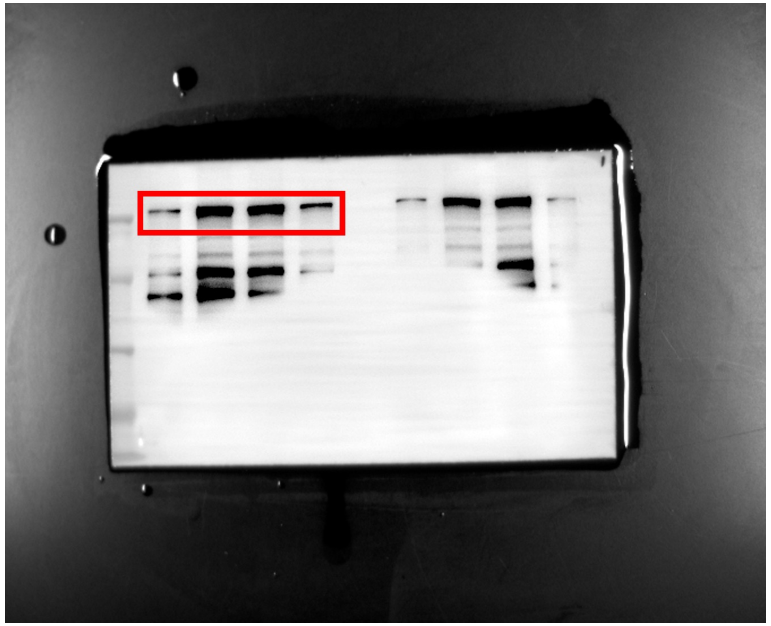
S5I-FASN

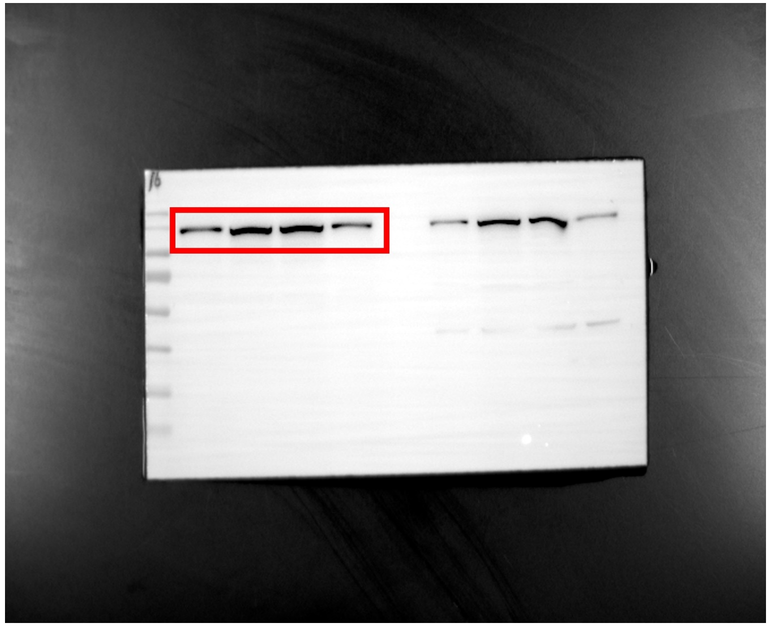
S5I-ACLY

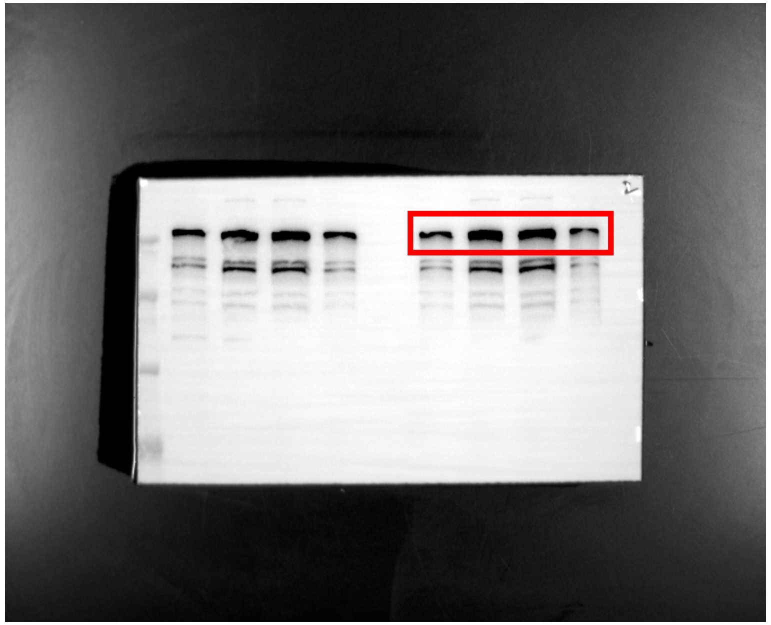
S5I-ACC

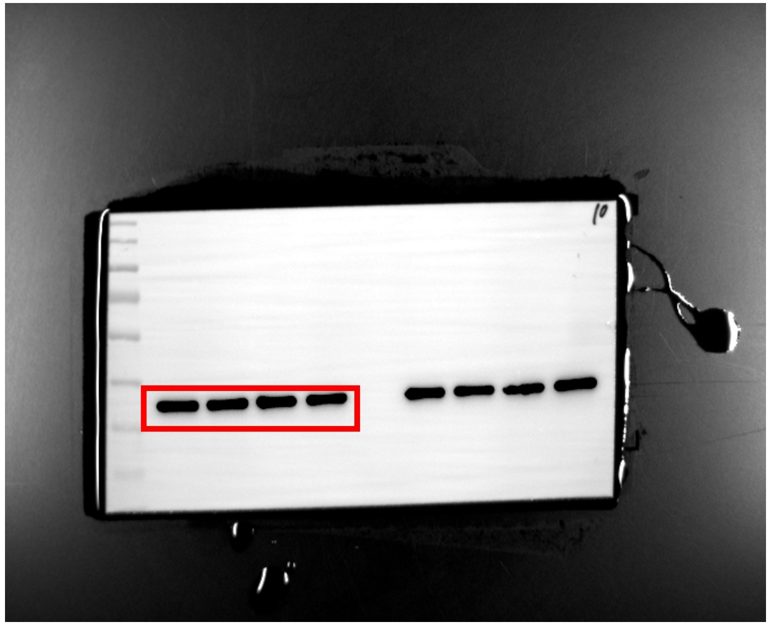
S5I-GAPDH

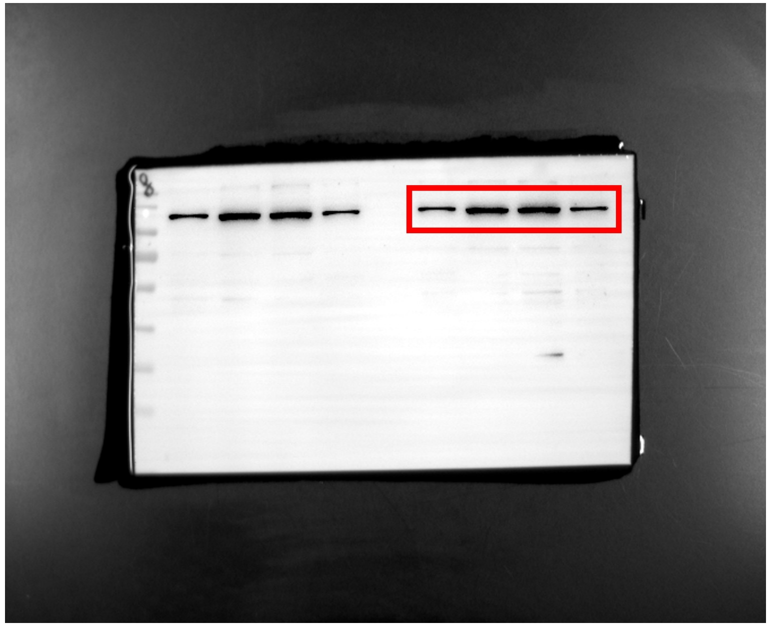

S5K-CD133

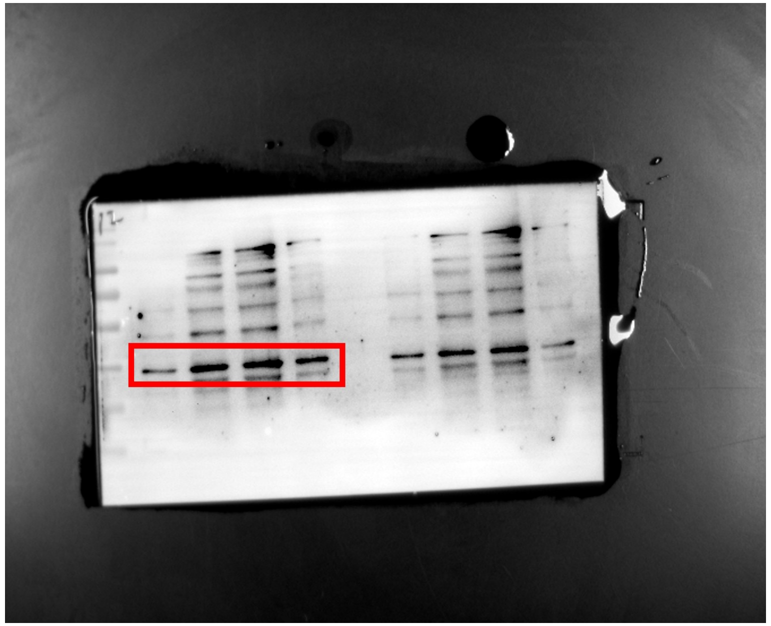

S5K-EpCAM

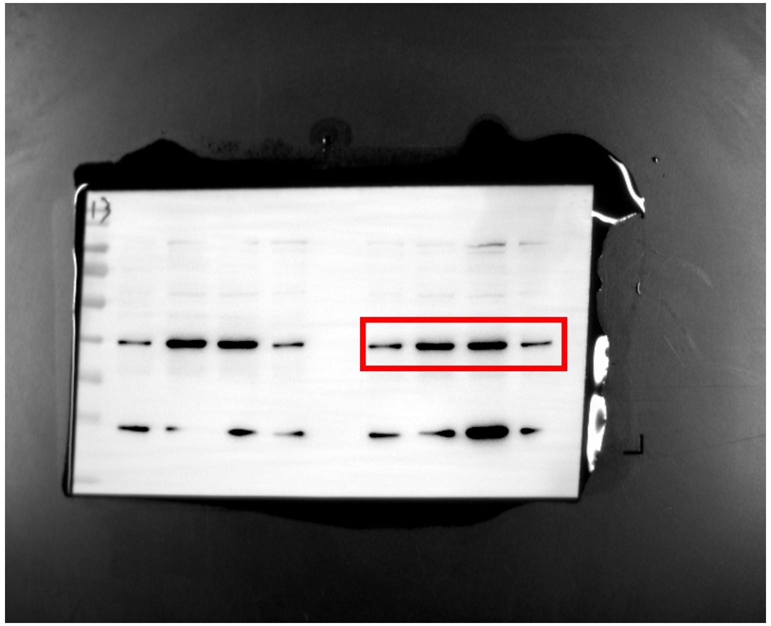

S5K-Nanog

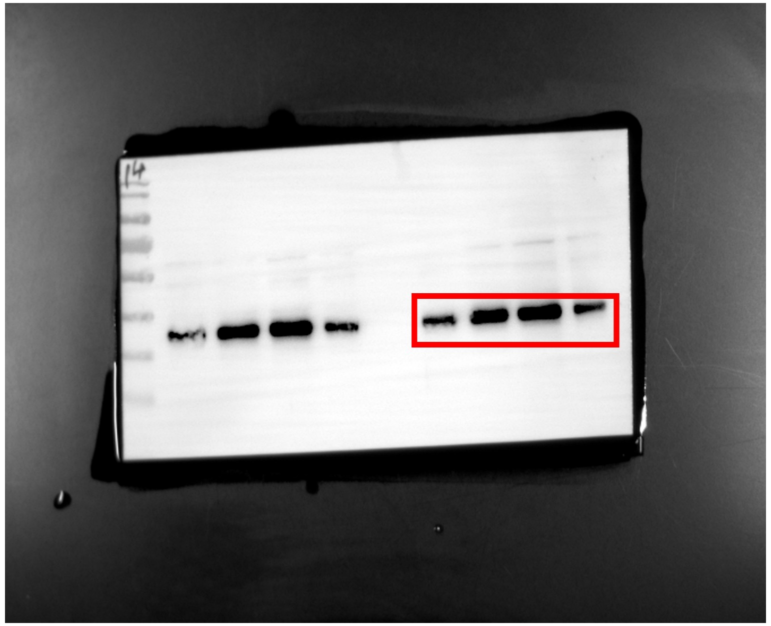

S5K-SOX2

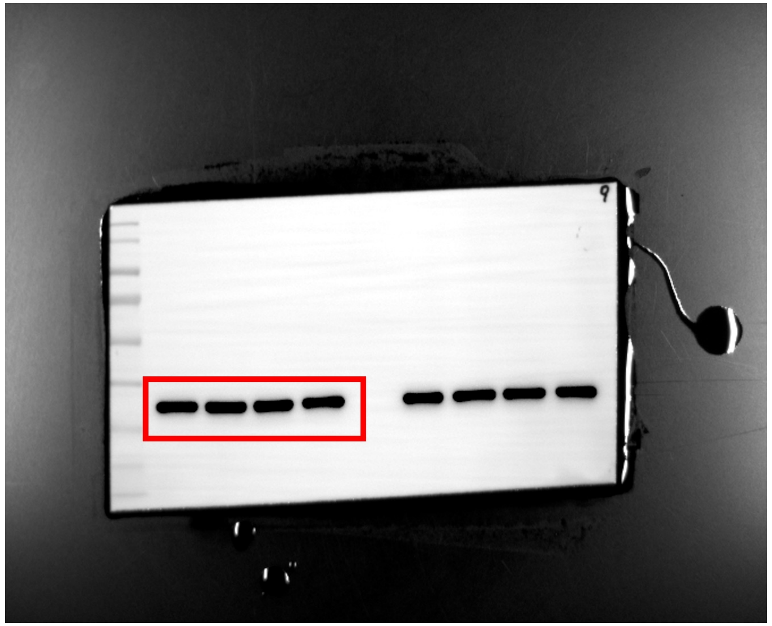

S5K-GAPDH

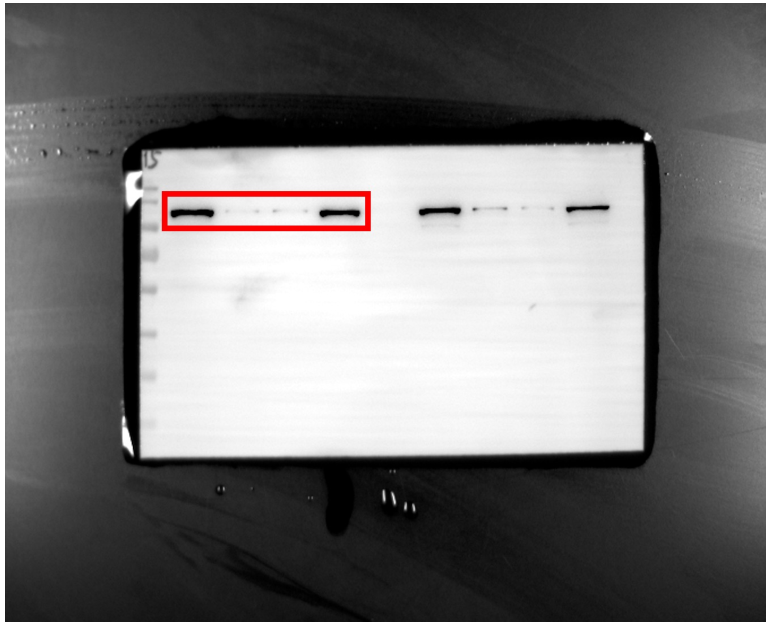

S5L-DLG1

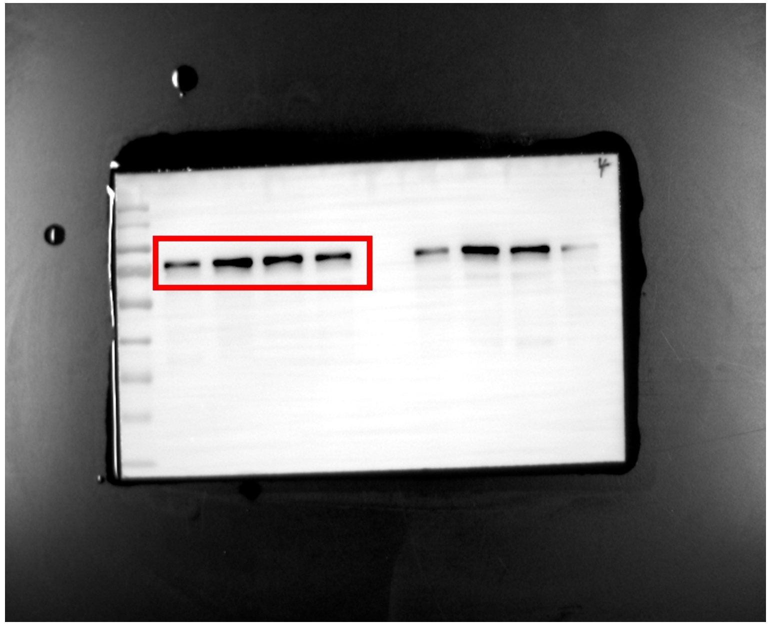

S5L-Nuc-YAP

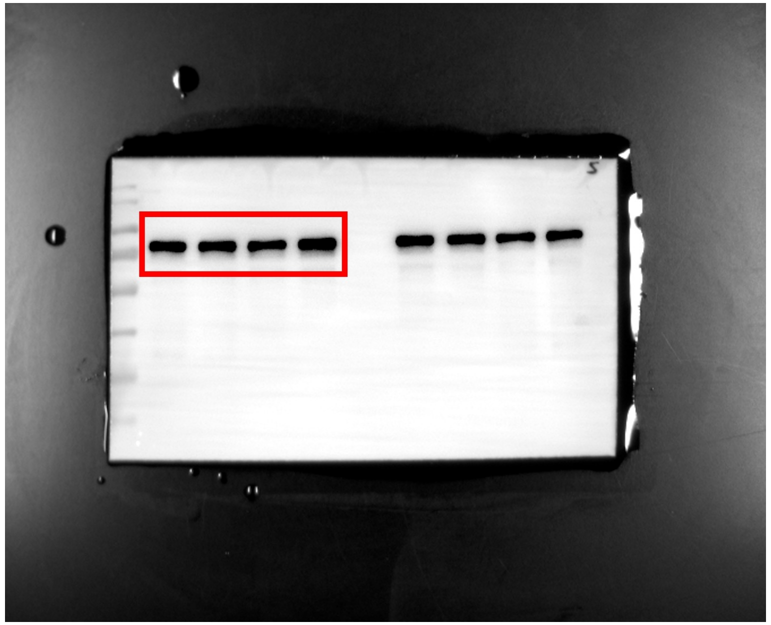

S5L-Cyt-YAP

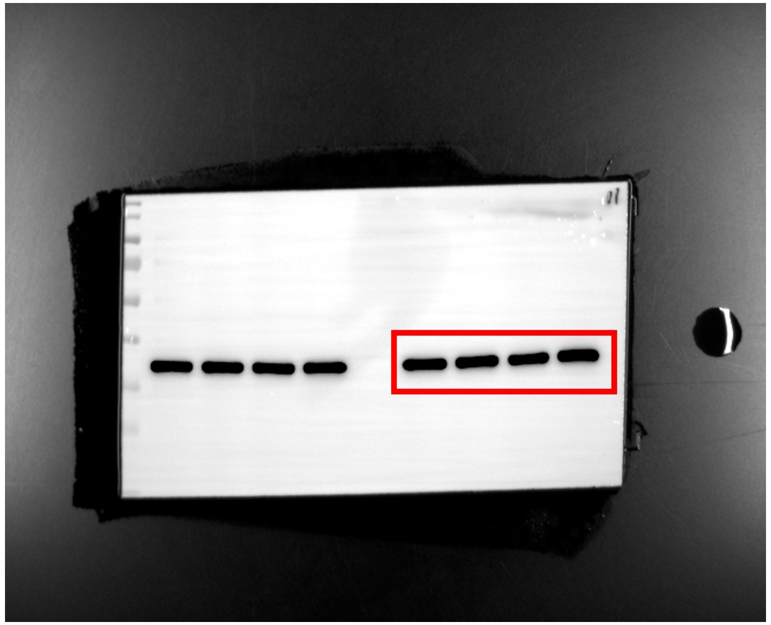

S5L-GAPDH
